# Identification and development of novel salt-responsive candidate gene based SSRs (cg-SSRs) and *MIR* gene based SSRs (mir-SSRs) in bread wheat (*Triticum aestivum*)

**DOI:** 10.1038/s41598-021-81698-3

**Published:** 2021-01-26

**Authors:** Geetika Mehta, Senthilkumar K. Muthusamy, G. P. Singh, Pradeep Sharma

**Affiliations:** 1 Division of Crop Improvement, ICAR-Indian Institute of Wheat and Barley Research, Agrasain Marg, PO BOX-158, Karnal, Haryana 132 001 India; 2 Division of Crop Improvement, ICAR-Central Tuber Crops Research Institute, Thiruvananthapuram, India

**Keywords:** Plant breeding, Abiotic

## Abstract

Salt stress adversely affects the global wheat production and productivity. To improve salinity tolerance of crops, identification of robust molecular markers is highly imperative for development of salt-tolerant cultivars to mimic yield losses under saline conditions. In this study, we mined 171 salt-responsive genes (including 10 miRNAs) from bread wheat genome using the sequence information of functionally validated salt-responsive rice genes. Salt-stress, tissue and developmental stage-specific expression analysis of RNA-seq datasets revealed the constitutive as well as the inductive response of salt-responsive genes in different tissues of wheat. Fifty-four genotypes were phenotyped for salt stress tolerance. The stress tolerance index of the genotypes ranged from 0.30 to 3.18. In order to understand the genetic diversity, candidate gene based SSRs (cg-SSRs) and *MIR* gene based SSRs (miR-SSRs) were mined from 171 members of salt-responsive genes of wheat and validated among the contrasting panels of 54 tolerant as well as susceptible wheat genotypes. Among 53 SSR markers screened, 10 cg-SSRs and 8 miR-SSRs were found to be polymorphic. Polymorphic information content between the wheat genotypes ranged from 0.07 to 0.67, indicating the extant of wide genetic variation among the salt tolerant and susceptible genotypes at the DNA level. The genetic diversity analysis based on the allelic data grouped the wheat genotypes into three separate clusters of which single group encompassing most of the salt susceptible genotypes and two of them containing salt tolerance and moderately salt tolerance wheat genotypes were in congruence with penotypic data. Our study showed that both salt-responsive genes and miRNAs based SSRs were more diverse and can be effectively used for diversity analysis. This study reports the first extensive survey on genome-wide analysis, identification, development and validation of salt-responsive cg-SSRs and miR-SSRs in wheat. The information generated in the present study on genetic divergence among genotypes having a differential response to salt will help in the selection of suitable lines as parents for developing salt tolerant cultivars in wheat.

## Introduction

Wheat being staple food, its production is important for global food security. To meet out the food demand, the production of wheat needs to be increased up to 90–120% by 2050. Globally, 430 M ha of land is affected by salinity and an area of around 5.8 × 10^6^ km^2^ of sodic soils have potential to develop into saline soils through the process of transient salinity^1–3^. These saline soils need to be utilized for food grain production to meet out the food demand of the growing population^4^. High yielding wheat cultivars grown under salt-affected soils were affected by physiological drought stress and ion toxicity, which resulted in yield reduction^5–7^. Salt stress also influences soil function, soil microbiome, pest infestation, etc. It is highly imperative to breed salt-resistant high yielding wheat cultivars that have the ability to thrive in saline soils^8^. Introgression of salt-stress responsive genes through marker-assisted and genomic selection has a potential impact on increasing the salt stress tolerance ability of high yielding bread wheat cultivars^5,9^. Moreover, availability of high quality draft sequence of wheat genome and other wheat genomic resources hastens the process of gene identification through comparative genomics approach and molecular marker development^10–12^. Therefore, functional characterization of genes and development of salt stress-responsive gene-based markers would be useful in breeding programs for improving salt tolerance in wheat^7,13,14^.

Plants employ special mechanisms including osmotic tolerance, ion exclusion tissue tolerance, redox equilibrium and others to combat salt stress^6,15^. Maintenance of low salt concentration in the cytoplasm was considered as a key component for plant cellular-tolerance towards salt stress^5,16^. Plants evolve a osmotic mechanism to compartmentalize the excess Na^+^ and Cl^−^ in vacuoles to maintain optimum salt concentrations in cytoplasm and organelles^6,17,18^. In *Arabidopsis*, mutants that failed in maintaining low cytosolic salt concentration were highly sensitive to salt stress^19^. Several salt-stress responsive genes with diverse cellular functions and miRNAs have been functionally validated for salt-stress tolerance in many crop plants including *Arabidopsis* and rice^20–25^. The Na^+^ ion exclusion capacity of bread wheat is higher than durum wheat, which increases the salt tolerance capacity of bread wheat than durum wheat. Introgression of *TmNax2* locus from *T. monococcum* to durum wheat increased the salt tolerance ability of durum wheat and also increased the yield up to 25% greater than non-introgressed lines grown in saline soil^26^. High-affinity K^+^ transporters (HKTs) play important role in regulation of Na^+^ concentration in wheat^6,7^. Over-expression of *TaNAC29* in *Arabidopsis* increased the salt-tolerance ability of transgenic *Arabidopsis* lines^27^. TaSRO1, a poly (ADP ribose) polymerase (PARP) domain containing protein regulate the reactive oxygen species homeostasis in wheat^28^. Over-expression of *TaAOC1,* a allene oxide cyclase functioning in the α-linolenic acid metabolism pathway, in both wheat and *Arabidopsis* increased the salt-tolerance ability of the transgenic lines^29^. Similiarly, transgenic wheat lines overexpressiong TaCHP, a zinc finger protein display enhanced salt tolerance ability than the wild type salinity-sensitive cultivar Jinan 177^30^. Transgenic Arabidopsis lines over-expressing the *TaNIP* wheat gene encoding for aquaporin protein showed increased tolerance for salt stress than wild type plants^31^. Overexpression of *TaOPR1*, a 12-oxo-phytodienoic acid reductases increase the salinity tolerance in transgenic wheat lines^32^. ClpATPase and HSP chaperones expression were increased under salt stress in bread wheat^12,33^. Over-expression of wheat *TaSTRG* in rice increased the salt-tolerance ability of transgenic rice lines^34^. Both, HKT gene based Na^+^ exclusion pathway and the SRO gene mediated ROS homeostasis mechanism play major role in imparting salinity tolerance in wheat^6,7^. Conversely, several genes with diverse functions were known to regulate the salt stress mechanism in plants, minimal efforts have been made to identify their orthologs in wheat. Thus, the availability of high quality genomic resources in wheat eases the process of the identification of salt stress responsive genes^10,11,14^. Hence, in this study, we have used the genomic information of the functionally validated genes from wheat and other crop plants including rice and identified their salt stress respective putative orthologs in wheat. Salt-stress, tissue and developmental stage specific expression pattern were studied using the normalized wheat RNAseq datasets. Salt-stress responsive cgSSR and miR-SSR markers were developed and screened in a set of 54 wheat genotypes differing in salt tolerance.

## Results

### Phenotypic screening of the wheat germplasm for salt tolerance

**Figure 1:**
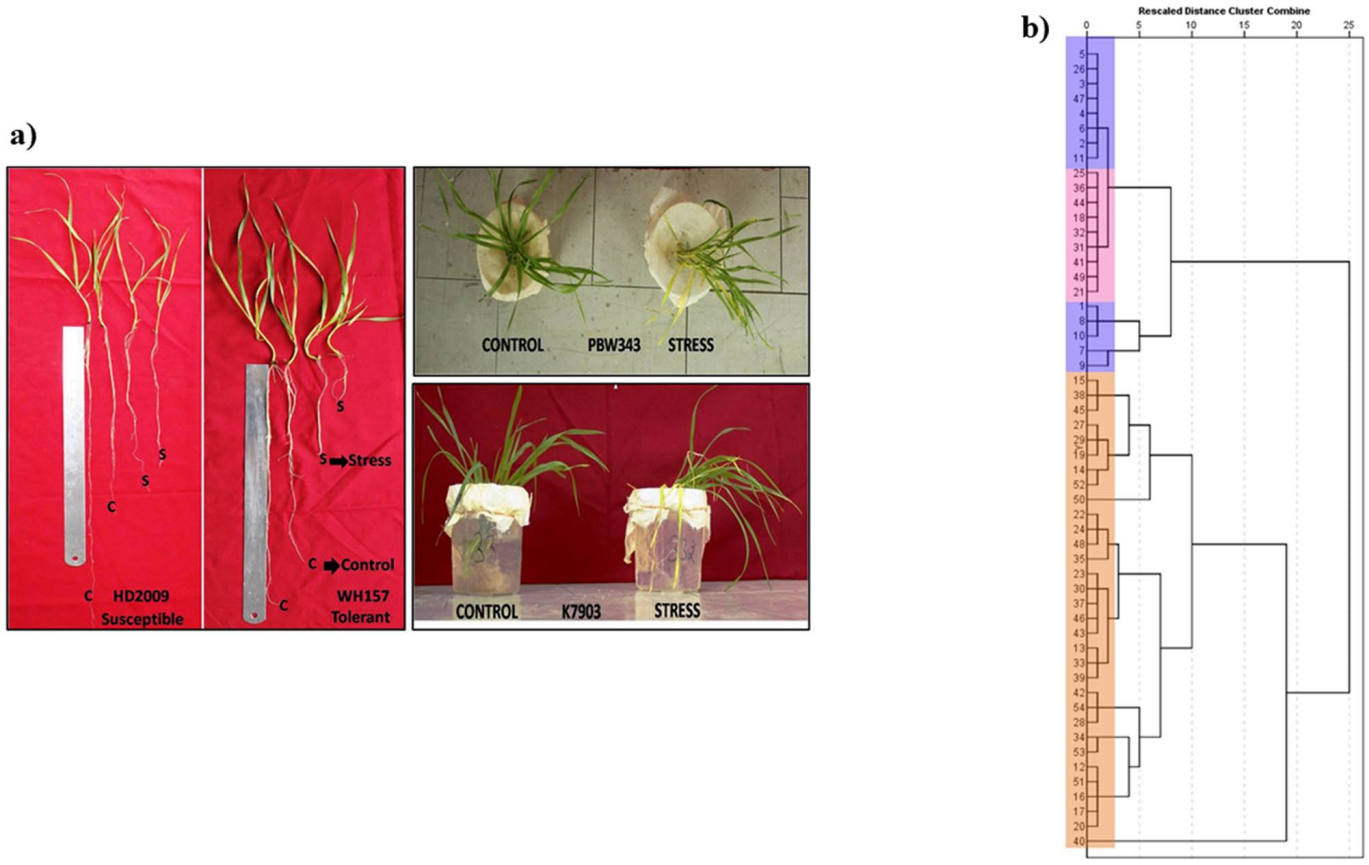
The phenotypic evaluation for salinity tolerance. (**A**) Effect of salt treatment on 54 diverse wheat genotypes under hydroponics conditions, (**B**) dengrogram showing phenotypic variability relationship between 54 wheat genotypes based upon phenotypic data. The cluster is made on the basis of all positive correlation of these traits i.e. total dry weight (TDW), chlorophyll content (SPAD) and sodium content in roots (NA) with salt tolerance index (STI). This dendrogram divides into two major cluster i.e. cluster I and cluster II. Cluster I contains total of 22 genotypes and all the genotypes comes under tolerant and moderately tolerant group. Cluster II contains all susceptible genotypes.

In many plant species, salt tolerant genotypes evolved genetic mechanisms to avoid/exclude the accumulation of Na^+^ and Cl^−^ salts in the cytoplasm and also have ability to maintain high K^+^/Na^+^ ratio in the cytoplasm^7,15,35–37^. In this study, the salt tolerance abilities of the 54 diverse wheat genotypes were estimated by studying the ability of genotypes to avoid/exclude the accumulation of Na^+^ and K^+^ salts, K^+^/Na^+^ ratio maintenance and stress tolerance index (STI) under salt stress and control conditions^35,38^ (Fig. 1a,b; Supplementary Table S1a and Supplementary Table S1b). Among the 54 genotypes studied, the stress tolerance index value ranges from 0.30 to 3.18, the maximum value was observed in the highly salt tolerant genotype KRL99 and the minimum in salt susceptible genotype HD4530 (Supplementary Table S1a). Interestingly, the genotypes having STI more than 2 comes under the category of most tolerant genotype i.e. KRL99, KRL35, KRL-3-4, KHARCHIA LOCAL, WH157, KHARCHIA65, KRL19 and KRL 1-4 displayed more than 2 STI and 27 genotypes having STI less than 1.00 comes under the category of salt susceptible (Supplementary Table S1a). The K^+^/Na^+^ ratio in shoot under salt stress ranged from 3.9 to 32.44 with the maximum value in PBW343 and the minimum in HI1500 whereas in root the value ranged from 14.3 to 57.8 with the maximum value in RAJ4037 and the minimum in KRL213 (Supplementary Table S1b). To determine the salt tolerance index, a total of 54 genotypes were screened in two conditions (i.e. in control and stress for number of traits. On this basis, 54 genotypes were divided into three categories (i.e. tolerant, moderately tolerant and susceptible). The highest positive correlation of STI is found with SPAD values; therefore the tolerant genotypes had SPAD values more than 38.00. The moderately tolerant genotypes had SPAD values ranging between 34.00 and 37.00 and the susceptible ones had values less than 34.00. According to the Pearson correlation analysis, SPAD (r = 0.82), total dry weight (r = 0.99) and sodium content (r = 0.46) also showed positive significant correlation with salt tolerance index.

### Identification of salt stress responsive and *MIR*-based genes in wheat

**Figure 2:**
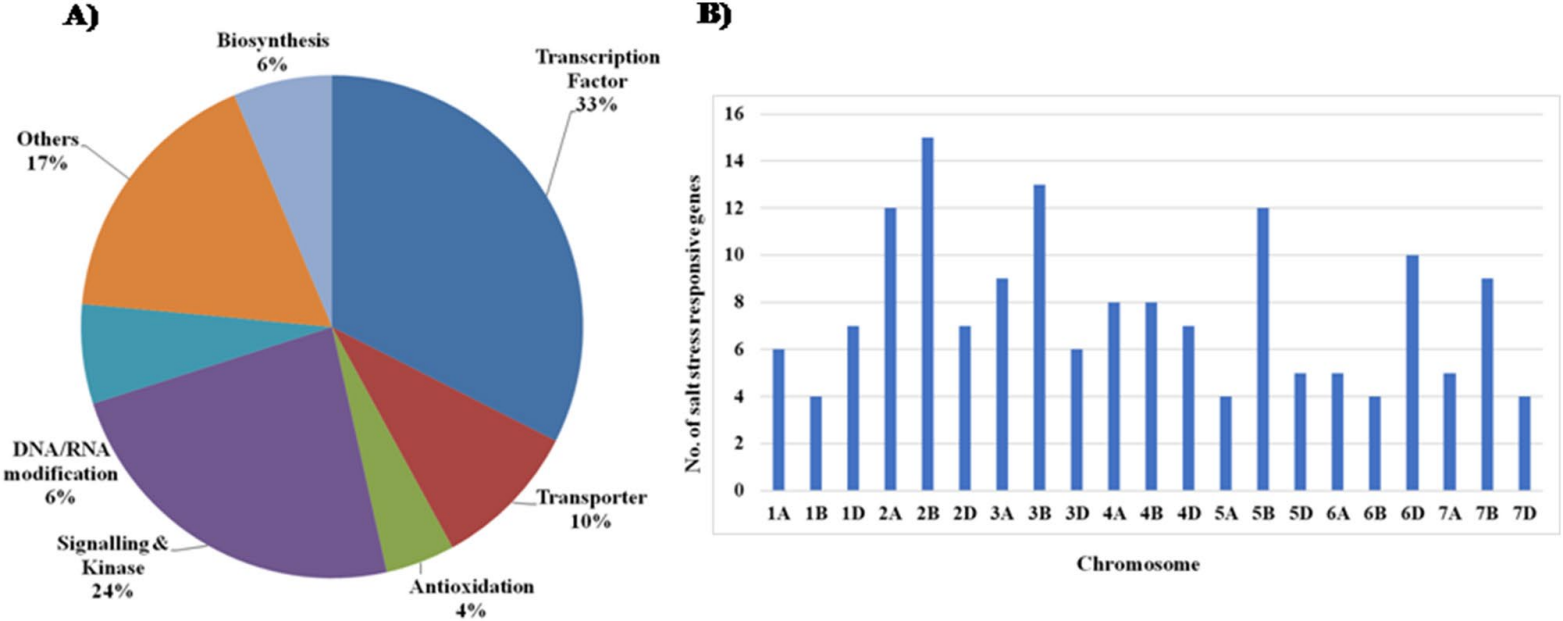
Frequency and distribution of salt stress responsive genes in wheat (**A**) Functional classes of identified salt responsive genes in wheat (**B**) Chromosomal distribution of salt stress responsive genes in wheat. Details of chromosomal location and scaffold regions of salt stress responsive genes were given in Supplementary Table S4.

Salt- responsive genes have been well characterized in crop plants^22,23,39,40^ as shown in Supplementary Table S2 and Supplementary Table S3. Additionally, 70 genes were functionally characterized for salt-responsiveness in wheat (Supplementary Table S2). Using the protein sequences of the functionally validated salt-stress responsive genes, a BlastP search was performed against the wheat proteome database^11^ to identify the respective salt-responsive wheat orthologs (Supplementary Table S2 and Supplementary Table S4). Our analysis led to the identification of 171 salt-responsive candidate genes in wheat (Fig. 2, Supplementary Table S4 and Table 1). In order to gain an insight into the various functions that Tacg-SSR containing salt stress responsive candidate genes were grouped into six functional groups. Among the groups, 33%, 24%, 10%, 6%, 6% and 4% of the total salt stress responsive genes belongs to transcription factors, signaling and kinase, transporter, biosynthesis, DNA/RNA modification and antioxidation, respectively (Fig. 2A and Supplementary Table S4). The chromosomal location and scaffold details of the identified salt stress responsive genes were retrieved from the wheat genome database^11^. Our analysis showed that salt responsive genes are widely distributed in all the 21 chromosomes of bread wheat, with the maximum of 15 genes located in 2B, followed by 13 genes in 3B, Twelve genes were located in each of two chromosomes, 2A and 5B. Ten genes were located in 6D while nine genes were located in each of two chromosomes, 3A and 7B. Eight genes were located in each of two chromosomes, 4A and 4B. Seven genes were located in each of three chromosomes, 1D, 2D and 4D. Six genes were located in each of two chromosomes, 1A and 3D, while five genes were located in each of three chromosomes, 5D, 6A and 7A. Four genes were located in each of four chromosomes, 1B, 5A, 6B and 7D (Fig. 2B and Supplementary Table S4). The chromosomal location of one gene was not assigned in the wheat genome database (Supplementary Table S4). The cellular localization of the salt-responsive genes was predicted using WoLF PSORT^41^ and TargetP 1.1 server^42^ (Supplementary Table S4). The nucleotide sequences of the salt-stress responsive *MIR* genes were used in a query against the wheat small RNA database that identified the respective salt-responsive wheat orthologs (Table 1 and Supplementary Table S2). The initial analysis further led to the identification of 24 *MIR* genes, 14 duplicated *MIR* genes sequences were removed subsequently using Clustal Omega tool^43^. Finally, we identified 10 salt stress-responsive *MIR* genes in wheat (Table 1). Around 988 genes were under the regulation of 10 *MIR* genes. The details of the target transcripts of *MIR* genes are given in Supplementary Table S5.

**Table 1:** Salt stress responsive *MIR* genes in Wheat.

Gene	Ensembl plants gene ID	Chromosomal location	Chromosome survey sequence
*Tamir160a*	EPlTAET00000001376	5D	5D:138155564:138155659
*Tamir169a*	EPlTAET00000007608	5D	IWGSC_CSS_5DL_scaff_4542798:2336:2470
*Tamir169e*	EPlTAET00000000922	5D	5D:107380642:107380789
*Tamir169k*	EPlTAET00000008126	5B	5B:190176930:190177077
*Tamir169m*	EPlTAET00000007781	5D	5D:107380659:107380784
*Tamir169n*	EPlTAET00000000696	7B	7B:111090146:111090267
*Tamir171a*	EPlTAET00000007580	6B	IWGSC_CSS_6BS_scaff_3031431:1976:2081
*Tamir171b*	EPlTAET00000003067	1A	IWGSC_CSS_1AL_scaff_3909165:20040:20143
*Tamir172a*	EPlTAET00000008820	1A	1A:226362546:226362702
*Tamir172d*	EPlTAET00000000231	6A	6A:203905904:203906014

### Spatio-temporal expression analysis of salt responsive genes in wheat

**Figure 3:**
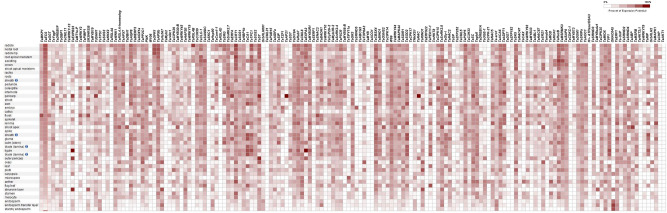
RNA-seq based tissue-specific expression profiles of salt stress responsive genes in wheat. (**A**) Expression profile. (**B**) Developmental stage-specific expression profile. The expression datasets were normalized and presented as heat maps using meta-analysis tool. The intensity of color represents the percent expression profile of the genes as described in the legend bar.

**Figure 4:**
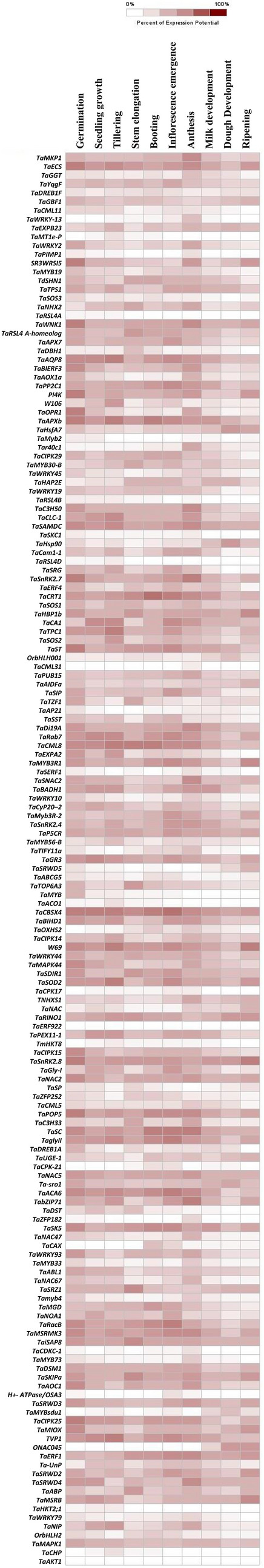
RNA-seq based developmental-specific expression profiles of salt stress responsive genes in wheat. The expression datasets were normalized and presented as heat maps using meta-analysis tool. The intensity of color represents the percent expression profile of the genes as described in the legend bar.

Tissue-specific and stage-specific expression pattern of identified 161 wheat salt-responsive genes were studied using the normalized wheat mRNA-seq expression datasets developed by the International Wheat Genome Sequencing Consortium (IWGSC) through the Genevestigator database^44,45^. In total, the expression of salt responsive genes were studied in the 43 tissues and ten developmental stages (Figs. 3, 4). Our analysis showed that all 161 salt stress responsive genes (except *TaAKT1*) were expressed in at least one tissue/development stage (Figs. 3, 4). In particular, the genes like *TaMKP1, TaECS, TaGGT, TaYqgF, TaGBF1, TaCML11, TaEXPB23, TaWRKY2, SR3WRSI5, TaMYB19, TdSHN1, TaTPS1, TaNHX2, TaWNK1, TaRSL4 A-homeolog, TaAPX7, TaAQP8, TaBIERF3, TaAOX1a, TaPP2C1, PI4K, TaAPXb, TaHsfA7, TaCIPK29, TaMYB30-B, TaHAP2E, TaWRKY19, TaC3H50, TaCLC-1, TaSAMDC, TaSRG, TaSnRK2.7, TaERF4, TaCRT1, TaSOS1, TaHBP1b, TaTPC1, TaSOS2, TaST, TaPUB15, TaAIDFa, TaSIP, TaDi19A, TaRab7, TaCML8, TaMYB3R1, TaSNAC2, TaBADH1, TaMyb3R-2, TaSnRK2.4, TaP5CR, TaCBSX4, TaCIPK14, W69, TaWRKY44, TaMAPK44, TaSDIR1, TaSOD2, TNHXS1, TaNAC, TaRINO1, TaPEX11-1, TaCIPK15, TaSnRK2.8, TaNAC2, TaCML5, TaPOP5, TaSC, TaglyII, TaNAC5, Ta-sro1, TaACA6, TabZIP71, TaSK5, TaNAC47, TaWRKY93, TaABL1, TaSRZ1, Tamyb4, TaMGD, TaNOA1, TaRacB, TaMSRMK3, TaiSAP8, TaDSM1, TaSKIPa, TaAOC1, TaSRWD3, TaCIPK25, TaMIOX, TVP1, TaERF1, Ta-UnP, TaSRWD2, TaABP, TaMSRB, TaWRKY79, TaNIP* and *TaMAPK1* were expressed in most of the tissues and in all ten developmental stages, as well (Figs. 3, 4). Nevertheless, there were significant differences in their level of expression (Figs. 3, 4). Expression of *TaEXPB23, TaHsfA7, TaRINO1 and TaMYBsdu1* were higher in the aleurone layer. The expression of *TaGBF1, TaHsp90, TaTZF1* and *TaSRWD5* were higher in embryo tissue whereas *ONAC045* and *Ta-sro1* expression were higher in endosperm tissue. The expression of *TabZIP71* and *TVP1* genes were higher in internodes. *SR3WRSI5*, *TaWNK1* and *TaCIPK15* expression were higher in coleoptile tissues. The expression of *TaEXPB23, TaST, TdSHN1, TaERF4, TaAP21 and TaNAC* were higher in pericarp tissues. *TaWRKY-13*, *TaC3H33*, *TabZIP71* and *TaTPS1* expression were higher in spikelet tissue. *TaSnRK2.8, TaSK5, TaSRZ1* and *TaABP* (Fig. 3) were higher in root tissues. The expression of *TaECS, SR3WRSI5, TaWNK1, PI4K, TaOPR1, TaAPXb, TaSnRK2.7,TaST, TaPOP5, TaSK5, TaRacB, TaMSRMK3, TaAOC1* and *TaCIPK25* were increased during germination stage (Fig. 4). *TVP1, TaACA6, TaglyII, TaCBSX4, W69, TaSOD2, TaCML8, TaTPC1, TaAPXb and TaAQP8* expression were higher at tillering stage (Fig. 4). The expression of *TaSK5, TaACA6, TaSC, TaglyII, TaPOP5, TaCBSX4, TaCML8, TaCRT1* and *TaAPXb* were higher in booting stage (Fig. 4). *TabZIP71, TaSC, TaSnRK2.7, TaWNK1, TaMKP1* and *TaECS* expression were higher at anthesis stage (Fig. 4). The expression of *TaECS,TaSnRK2.8, W69, TaMYB3R1, PI4K and SR3WRSI5* were higher in ripening stage (Fig. 4).

### Expression analysis of salt stress responsive genes in wheat

**Figure 5:**
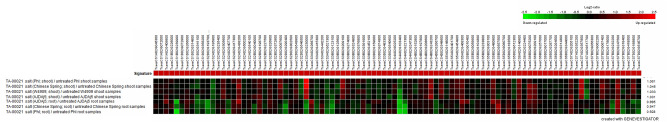
RNA-seq based salt stress-specific expression profiles of salt stress responsive genes in wheat. The expression datasets were normalized and presented as heat maps using meta-analysis tool. The intensity of color represents the percent expression profile of the genes as described in the legend bar.

The salt stress-responsive expression patterns of the identified genes were studied using normalized wheat genome array datasets^33,44,46^ Out of 171 genes, 96 genes have the genome array expression information under salt stress conditions (Fig. 5). Shoot and root-tissue specific expression of the identified genes during tillering stage were studied using the normalized salt-stress specific expression datasets developed by Mott and Wang^46^ through the Genevestigator tool (Fig. 5). The expression of 11 genes viz., *TaAOX1a, TaHAP2E, TaNAC2, TaNAC5, TabZIP71, TaWRKY93, TaNAC67, TaSRZ1, TaMYBsdu1, TaMSRB* and *TaCLC-1* were upregulated, whereas the expression of seven genes viz., *TaTPS1, TaWNK1, TaST, TaPUB15, TaBADH1, TaABCG5* and *ONAC045* were downregulated both in shoot and root tissues during tillering stage under salt stress (Fig. 5). Ten genes viz., *TaAPX7, TaCam1-1, TaSST, TaSOD2, TaPEX11-1, TaGly-I, TaMIOX, Ta-UnP, TaNIP* and *TaERF4* were upregulated, whereas the expression of two genes (viz., *TaCRT1* and *TaTZF1*) were both down-regulated in shoot tissues during the tillering stage under salt stress (Fig. 5). In root tissues, five genes viz., *TaGBF1, TaBIERF3, TaTPC1, OrbHLH001* and *Ta-sro1* were up-regulated whereas, the expression of thirteen genes viz., *TaMT1e-P, TaWRKY2, TaSOS3, TaRSL4D, TaAQP8, TaOPR1, TaSRG, TaSIP, TaACO1, TaRINO1, TaABP, TaHKT2;1* and *TaCHP* were down-regulated during the tillering stage under salt stress (Fig. 5). Interestingly, the expression of *TaCA1* was up-regulated in shoot tissues and down-regulated in the root tissue during tillering stage under salt stress (Fig. 5).

### qPCR expression analysis of salt responsive genes

**Figure 6:**
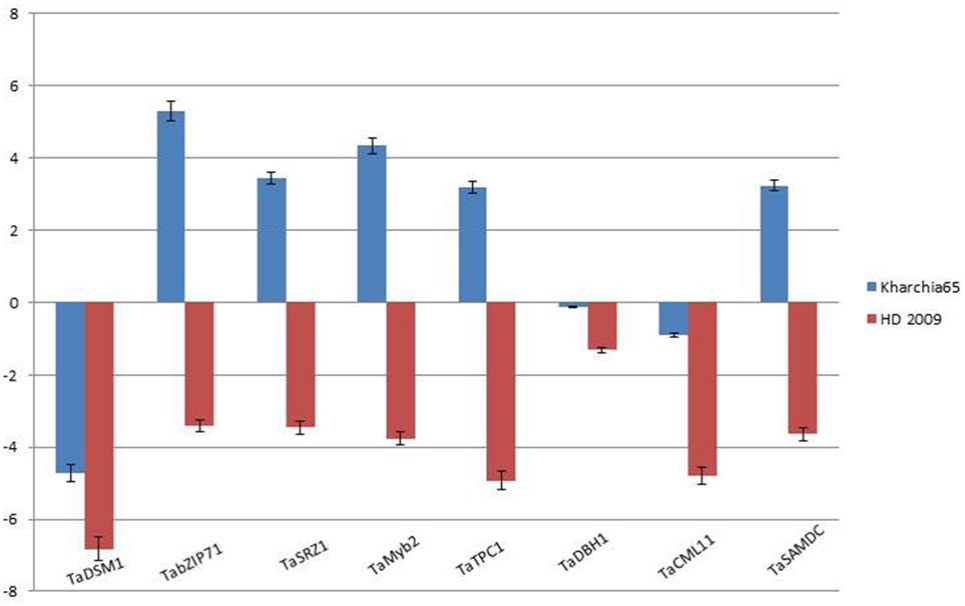
Expression analysis of salt responsive genes through qPCR.

The magnitude of expression of eight randomly selected salt-stress responsive genes were studied in the root tissues of fourteen days old seedling under salt stress. *TaDSM1* and *TaCML11* expression was downregulated in both the genotypes (Fig. 6). Interestingly, the expression of five genes viz., *TabZIP71, TaSRZ1, TaMyb2, TaTPC1* and *TaSAMDC* were upregulated in salt-tolerant genotype Kharchia65, whereas, all the five genes were downregulated in the salt-susceptible genotype HD 2009 (Fig. 6). The expression pattern of selected salt-stress responsive genes is largely in correspondence with the array datasets (Fig. 6). The primer details used for the expression studies is given in Supplementary Table S6.

### Identification and validation of salt-responsive cg-SSRs and mir-SSRs in wheat

**Figure 7:**
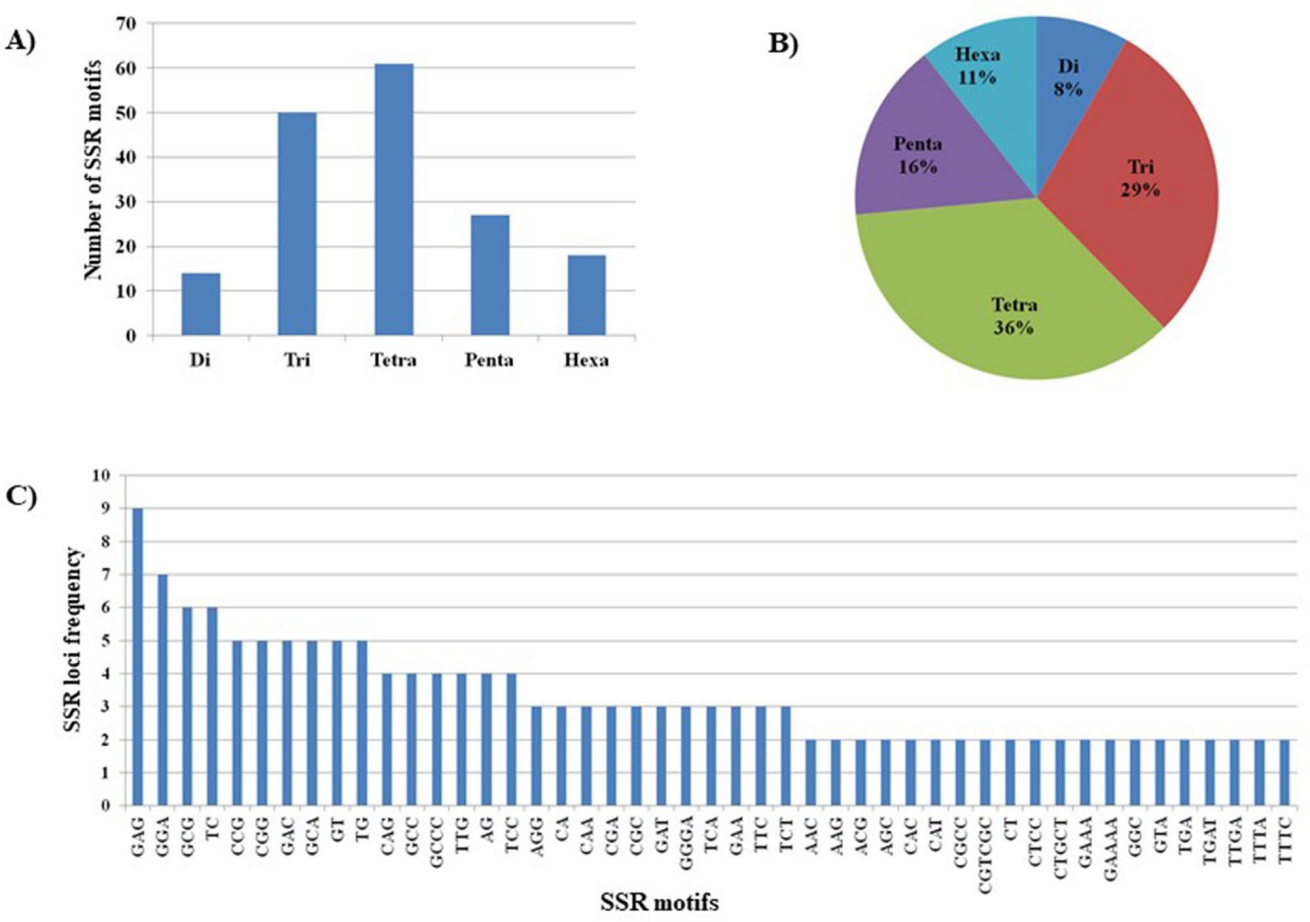
Frequency and distribution of salt stress responsive cg-SSRs and MIR-SSRs in wheat. (**A**) Number of different SSR motifs found, (**B**) Percentage of different SSR loci present in salt stress responsive genes (**C**) SSR loci frequency in salt stress responsive genes (only ≥ 2 times repeated SSR loci shown in the figure, For completed list refer Supplementary Table 3.

Salt stress-responsive candidate gene-based SSRs (cg-SSR) and *MIR* gene based SSRs (miR-SSRs) were mined from the identified wheat salt stress responsive genes using the BatchPrimer3 tool^47,48^. Out of 171 genes (including 10 *MIR* genes) screened, 115 genes yielded a total of 264 SSR loci (69%) (Fig. 7A,B, Supplementary Table S2, Supplementary Table S3 and Supplementary Table S7). List of salt—responsive genes harboring SSR motifs with their respective ensemble plants LOC ID, their functionality, motif repeatability and SSRs location were detailed in Supplementary Table S7. In rice, tri-nucleotide repeats were the largest motif found in salt-responsive genes^22^. In our study, the tetra-nucleotide repeats were the largest motif, comprising about 36% followed by the tri-nucleotide repeat motifs comprising 29% whereas, di-, penta- and hexa-nucleotides formed small share having 8%, 16% and 11% respectively (Fig. 7A,B). The number of repetitions of a motif varied from 3 to 19, among which, a motif with four reiterations (a motif repeated four times) were higher in frequency followed by three, five, six and seven repetition (Fig. 7C; Supplementary Table S7). The salt-responsive SSRs were present on all the 21 chromosomes of wheat (Supplementary Table S7). Conversely, the distribution of SSRs was not equal among the chromosomes, For example, the maximum frequency (14%) of salt stress-responsive gene-based SSR loci were found on chromosome 2B, whereas the least (0.1%) was found on chromosome 1B (Supplementary Table S7). Chromosome 1A, 3B, 4A, 4B, 5B, 5D, 6A, 6D and 7A were found to contain more than 5% SSR marker loci (Supplementary Table S7). Out of 264 SSR loci, we randomly selected 53 SSR loci, including 10 *MIR* gene based SSRs for wet lab validation (Supplementary Table S7).

### Allele scoring and data analysis

**Figure 8:**
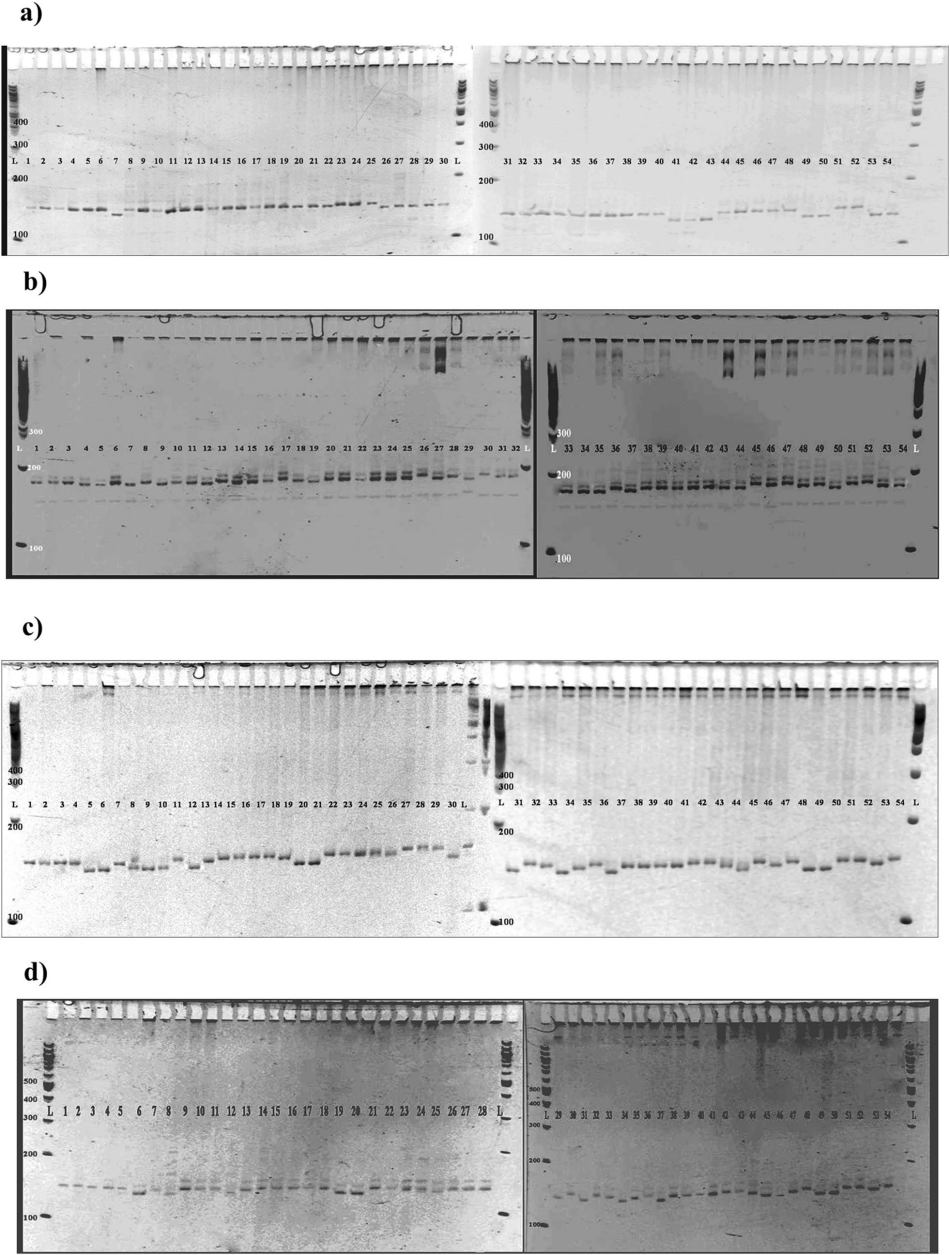
Amplification of genomic DNA from different wheat genotypes with (**A**) Tacg-SSR11, (**B**) TamiR-160a, (**C**) TamiR-169k and (**D**) TamiR-169l. L represents 100 bp ladder.

To test the amplification and polymorphism of the identified genic-SSRs, 53 SSRs chosen randomly were synthesized for validation in a set of 54 wheat genotypes. Based on a polymorphism survey, 18 polymorphic SSR (10 cg-SSRs and 8 miR-SSRs; Table 2) were identified and used for generating polymorphism profiles of the selected panel of wheat genotypes. These primers generated a total of 49 alleles across the wheat genotypes analysed (Table 2). The multiallelic SSR markers were run on 6% PAGE gel electrophoresis. The banding pattern of Tacg-SSR21, TamiR160a-SSR, TamiR169k-SSR and TamiR169l-SSR in 54 diverse wheat genotypes as shown in Fig. 8A–D, respectively. The lowest and highest amplicon size was 143 bp and 238 bp amplified with Tacg-SSR37 and Tacg-SSR17 markers, respectively (Table 2). The PIC value denotes the allelic diversity among the 54 genotypes. The miR-SSRs (TamiR-172d-SSR) exhibited the lowest PIC value (0.07) while the highest PIC value (0.67) obtained with the Tacg-SSR19 (Table 2) with the mean value of 0.44. Out of these 18 polymorphic SSRs, Tacg-SSR18 and TamiR160a-SSR displayed highest variation (5 alleles/marker), followed by TamiR169m a-SSR (4 alleles/marker) whereas Tacg-SSR1, Tacg-SSR11, Tacg-SSR21, Tacg-SSR23, Tacg-SSR24, Tacg-SSR37, TamiR 169i-SSR, TamiR172a-SSR, TamiR 171a-SSR and TamiR 172d-SSR displayed 2 allele/marker on PAGE gel electrophoresis (Table 2).

**Table 2:** Details of polymorphic Tacg-SSRs and TamiR-SSRs used in this study.

S. no.	Marker	Primer sequence	Expected alleles	Observed alleles	No of alleles	TM	PIC
1	Tacg-SSR1	F 5′GAAGATGAAGAGTCGGATGA 3′ R 5′GTACTCCTCCTTGCTGATCTC 3′	186	181–186	2	60	0.302
2	Tacg-SSR8	F 5′GTCTCGTCTCCCCGCCTCA 3′ R 5′ACAGTTCCGACACCACAA 3′	187	160–190	3	60	0.535
3	Tacg-SSR11	F 5′GCTGTAGTTGTGCTCCTTTTA 3′ R 5′ACAAGGCATAGCTCATACTCC 3′	146	150–155	2	60	0.253
4	Tacg-SSR17	F 5′AACAACCTCCAACGCTCT 3′ R 5′GTCGAAGGACCGGAAGAT 3′	238	230–400	3	62	0.655
5	Tacg-SSR18	F 5′CCAACTTGTTTGGGACTAAAG 3′ R 5′AAACGGCACCTCTACATAACT 3′	151	140–490	5	61.6	0.412
6	Tacg-SSR19	F 5′TACCCCAAAGTGAGTTCTACA 3′ R 5′CACCAGCTTTAGGTGCAT 3′	284	280–300	3	60	0.666
7	Tacg-SSR21	F 5′CTAGCTTCGTTTGTCTGTTGT 3′ R 5′GATGTAGTTGGCGAGGAG 3′	148	136–150	2	61.6	0.370
8	Tacg-SSR23	F 5′GGTCTCTAACCATGTATCGTG 3′ R 5′AAACTAGTAAGCATGCACTCG 3′	172	169–190	2	56	0.499
9	Tacg-SSR24	F 5′GCATCAGGTTCTGTATCAATC 3′ R 5′ATCAGGAGCCAGTAGAAAATC 3′	184	190–210	2	56	0.50
10	Tacg-SSR37	F 5′AGCATTGACCCCAAATATC 3′ R 5′TCGAAAGGGTATAGGCTTAGT 3′	143	150–200	2	62	0.491
11	TamiR 160a-SSR	F 5′GAGGTGAAAACAATGGGATA 3′ R 5′CCAGGAATCTAAAGCAACC 3′	174	160–189	5	61.6	0.662
12	TamiR 169i-SSR	F 5′ACTCCTACAAAACATGCAGAG 3′ R 5′GTGACTCTTATCGTTCATGCT 3′	151	175–195	2	61.6	0.499
13	TamiR 172a-SSR	F 5′ATGTATAGGACAAAGGGAAGC 3′ R 5′ATCAAGATTCACATCCATCC 3′	160	150–180	2	61.6	0.444
14	TamiR 169k-SSR	F 5′GTGTGTGTGGAGAGAGAGAGA 3′ R 5′TATATCCACAGGCAAGTCATC 3′	149	152–163	3	60	0.652
15	TamiR 169m-SSR	F 5′TATATCCACAGGCAAGTCATC 3′ R 5′TGCCATGTAGAGAGAGAGAGA 3′	152	150–300	4	62.6	0.558
16	TamiR 171a-SSR	F 5′CGACGAGCAGTGAGATATAAG 3′ R 5′GTCCGTCGTAAACCTAACATA 3′	171	169–174	2	60	0.460
17	TamiR 172d-SSR	F 5′TATTAAGTGCCTCTGCCAGT 3′ R 5′GAGATTATTGTGGTACGTGGA 3′	158	160–190	2	56	0.071
18	TamiR 169l-SSR	F 5′TCTCACTAGACCCCTCTCTTC 3′ R 5′GTTTGAGGTGCTACAAATGG 3′	145	137–145	3	56	0.650

### Analysis of genetic diversity

**Figure 9:**
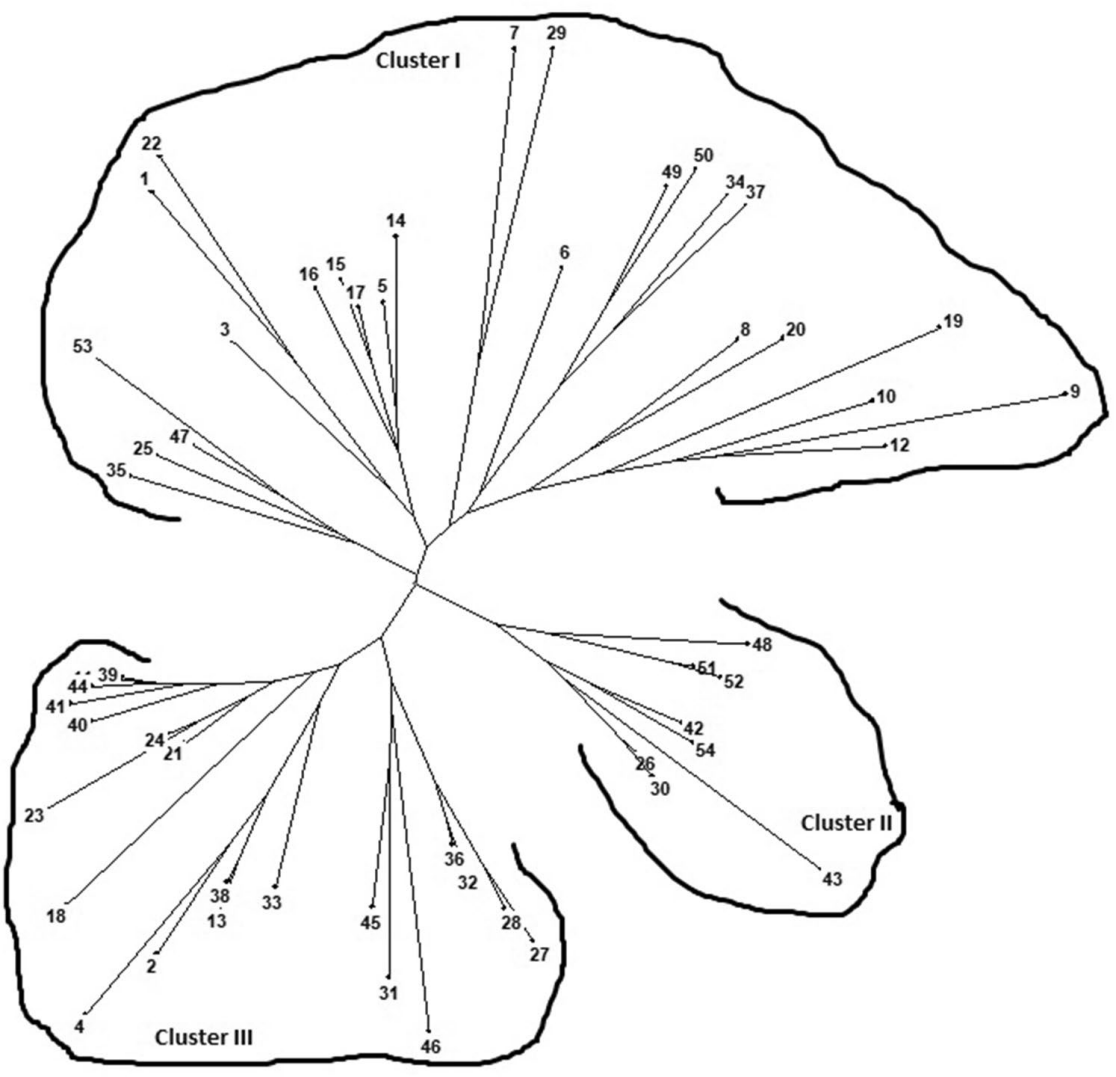
Dendrogram generated from UPGMA analysis based on salt stress responsive cg-SSRs and MIR-SSRs segregation data of 54 wheat genotypes. The details of the genotypes given in the Supplementary Table 1.

The binary data generated from 18 polymorphic SSR markers profiling of 54 contrasting wheat genotypes were used to study the genetic diversity by dissimilarity analysis and factorial analysis through cluster analysis (Table 2). The binary data deduced from their respective SSRs profiles divided all the 54 wheat genotypes into three major clusters (Fig. 9). The salt tolerant wheat genotypes were found to be more diverse than salt susceptible genotypes. The majority of salt tolerant wheat genotypes/genetic stocks were grouped into a single cluster (Cluster I) including KRL210, KRL213, KRL99, KRL-3-4, KRL35, WH157, KRL-1-4 and Kharchia65, which indicated the influence of pedigree and source on clustering pattern (Fig. 9) A majority of the lines derived from Kharchia65 local landrace have been utilized for salt wheat breeding programs in India. The smallest cluster (cluster II) contained salt susceptible genotypes HS240, RAJ3765, RAJ4210, PBW590, RAJ4079 and RAJ4037 except for HD2967 and WH1080 which are moderately tolerant to salinity. Cluster III consisted of twenty wheat genotypes representing moderately salt tolerance. These included NW1014, UP2338, PBW550, PBW343, GW322, DBW17, DBW90, HD2285, HD2932, NIAW34, DW1, K7903, HUW468, UP2382, VL616, MP4010, HUW510 and HD2808. In our study, genetic diversity analysis using salt stress-responsive gene-based cg-SSRs and miR-SSRs clearly distinguished the wheat genotypes into different groups based on their sensitivity to salinity (Fig. 9).

## Discussion

Salt stress is one of the serious abiotic stress that limits the crop yield, hence it is highly imperative to understand the molecular mechanisms of salt stress tolerance which would aid efforts to support/architect plant salt tolerance. It exerts a inimical effect on wheat by affecting crucial metabolic process including the process of photosynthesis, protein synthesis and lipid metabolism^6,7,49,50^. The effect of climate change will exacerbate the severity of salt stress in the future^51,52^. High yielding salt resistance cultivars need to be bred and introduced in the salt affected marginal lands to meet out the demand of the growing population^52,53^. Identification of salt stress-responsive genes based on SSRs will aid in breeding of salt stress tolerant high yielding wheat cultivars^3,7,8,54,55^. Although several studies reported the characterization of salinity tolerance using SSRs^56,57^, this work epitomized the genome identification of both salt-stress responsive gene-based cgSSRs and miR-SSR markers and its role in genetic diversity with respect to salinity tolerance in wheat.

Salt tolerance is a complex response involving a diverse set of genes having wide functional roles, i.e. transcription factors, transporters, ion channels, signalling and kinase, DNA/RNA modification, etc.^16,21,58^. Several genes have been functionally validated for salt tolerance in plants (Supplementary Table S2 and Supplementary Table S3). Minimal efforts have been made to identify their orthologs in wheat. In this study, 171 (including 10 *MIR* genes) salt-stress responsive wheat orthologs were identified using the sequence information of functionally validated rice salt-stress responsive genes (Supplementary Table S2, Table 1, Supplementary Table S3 and Supplementary Table S4). The identified wheat salt stress-responsive genes had diverse functional roles and were grouped into six functional groups, viz transcription factors, signaling and kinase, transporter, biosynthesis, DNA/RNA modification and antioxidation, representing 33%, 24%, 10%, 6%, 6% and 4%, respectively, owing to their diverse functional significance in wheat (Fig. 2A and Supplementary Table S4). The RNA-seq and array-based expression analysis showed spatiotemporal transcriptional regulation and salt stress responsive expression, indicating their diverse role in plant growth and development (Figs. 3, 4, 5, 6).

Recent studies had shown the presence of microsatellite motifs in salt-stress responsive candidate genes in rice^22,59^ and *MIR* genes^23,60^. However, very little information on experimental evidence for the occurrence of SSRs in miRNA precursors is available. The advantages of these markers over other marker types are high reproducibility and sufficient polymorphism, which have caused plant researchers to develop these marker systems^23^. Moreover, these markers (cg-SSR and miR-SSR) can be utilized finding marker trait association in genetic mapping studies. In rice, 180 cg-SSRs motifs were identified from 106 salt stress-responsive genes and genetic diversity analysis using 19 SSRs motifs differentiated the contrasting rice genotypes into tolerant and susceptible based on their sensitivity to salinity^22^. Chen et al.^60^ mined SSRs from ~ 9000 premature miRNAs representing 87 species and showed the presence of mono-nucleotide, di-nucleotide tri-, tetra-, penta- and hexa- nucleotide repeats in *MIR* genes. Taking into account the potential significant of gene based cg-SSRs and miR-SSRs, we mined SSRs in the identified salt-stress responsive genes (including *MIR* genes) of wheat and analyzed their allelic banding pattern on the differential response of the genotypes to salt stress. Out of 168 genes screened, 118 genes yielded a total of 264 SSR loci representing 69% of salt stress responsive genes (Fig. 7A,B and Supplementary Table S7). Tetra-nucleotide repeats were the most abundant repeats followed by the tri-nucleotide repeats as compared to di-, penta- and hexa-nucleotide repeats (Fig. 7A,B and Supplementary Table S7). In rice, 18 cg-SSRs displayed polymorphism and their banding pattern differentiated the contrasting rice genotypes into tolerant and susceptible group based on their sensitivity to salinity^23^. Similarly, miR172b-SSR, a *MIR* gene based SSR marker, was able to differentiate rice germplasm into a tolerant and susceptible group based on their sensitivity to salinity^23^. In our study, 10 cg-SSRs and 8 miR-SSRs (out of 10 miR-SSRs) were shown to be polymorphic and able to differentiate the contrasting wheat genotypes having differential responses to salt and the availability of significant genetic diversity among salt tolerant and susceptible genotypes (Table 2 and Figs. 8A–D, Fig. 9).

Ma et al.^61^ revealed 24.6% polymorphism in the developed genomic-SSR in common buckwheat was lower than the genomic-SSR polymorphism (26.7%) reported by the Konishi et al.^62^. In our study, the PIC value ranged from 0.07 to 0.62 and the dendrogram generated using the binary score of these allelic data grouped the 54 contrasting wheat genotypes for salt tolerance into three distinct clusters, of which one single cluster contained all salt susceptible genotypes and two of them contained tolerant wheat genotypes (Fig. 9). The salt tolerant wheat genotypes were found to be more diverse than salt susceptible genotypes. The smallest cluster (cluster II) also contained salt susceptible genotypes HS240, HD2967, RAJ3765, RAJ4210, PBW590, RAJ4037, and RAJ4079 except for HD2967 and WH1080, which are moderately salt tolerance. Cluster III consists of twenty wheat genotypes representing moderate salt tolerance. This includes NW1014, UP2338, PBW550, PBW343, GW322, DBW17, DBW90, HD2285, HD2932, NIAW34, DW1, K7903, HUW468, UP2382, VL616, MP4010, HUW510 and HD2808. The majority of salt tolerant wheat genotypes/genetic stocks were grouped into a single cluster (Cluster I) KRL210, KRL213, KRL99, KRL-3-4, KRL35, WH157, KRL-1-4 and Kharchia65 indicated the effect of pedigree and the source on grouping pattern (Fig. 9). In our study, phenotypic data and genetic diversity analysis using salt stress-responsive gene-based cg-SSRs and miR-SSRs clearly distinguished the wheat genotypes into different groups based on salt sensitivity (Fig. 9). Thus, the 18 gene-based SSRs developed in this study may be used to distinguish wheat genotypes based on their susceptibility to salt stress (Table 2 and Fig. 9).

## Conclusion

This study conducted an extensive genome-wide analysis, identified salt responsive-genes, and mined and validated gene-based cg-SSR and miR-SSRs, proving these methods as remarkable tools to distinguish wheat genotypes based on sensitivity to salt stress. Spatio-temporal expression regulation of salt-stress responsive genes indicated a distinct role of these genes in wheat growth and development. The cg-SSRs and miR-SSRs developed here can provide unique genomic resources for a wheat breeding program with delivering novel alleles to develop high yielding salt tolerance varieties/breeding lines. Hence, these markers have high potential of linkage and can be explored for gene pyramiding in wheat breeding programme for salt tolerance trait.

## Materials and methods

### Phenotypic screening of the wheat germplasm for salt tolerance

A diverse set of 54 wheat (*Triticum aestivum*) genotypes differing in salt tolerance ability was collected from the germplasm unit of ICAR-Indian Institute of Wheat and Barley Research (IIWBR), Karnal (Supplementary Tables S1a). These genotypes were phenotyped for salt tolerance in two conditions, i.e. control and stress for salt tolerance at the seedling stage^35,38^. The wheat seeds were surface sterilized with HgCl_2_ (0.1%) and grown in hydroponic conditions with full strength Hoagland solution. After 14 days, NaCl treatment was given for 7 days to 14 days old seedling in two conditions, i.e. control (2 mM) and stress (117 mM) in photoperiod of 14 h light and 10 h dark at 25 ± 2 °C. The Hogland solution was changed at every 2nd day to reduce the chance of contamination. The chlorophyll content was measured by chlorophyll meter (SPAD-502 Plus, Konica Minolta) and chlorophyll flourescence (Fv/Fm) was measured by chlorophyll fluorescence meter (Model OS30P+, opti-sciences, Inc. USA) from the mid rib of the 3rd leaf at 3rd day of salt treatment and last day of the experiment. For Na^+^ and K^+^ estimation, the salt stress treated plant root and shoot samples were collected from each genotype. The tissues were oven-dried at 65 °C for 5 days. From the dried samples of root and shoot, 70 mg of each sample were taken and digested in diacid having HNO_3_:HCLO_4_ (9:4). The Na^+^ and K^+^ concentration were measured from root and shoot samples by using microprocessor flame photometer^35^. The salt stress tolerance index was also calculated as described by Fernandez^38,63^.

$${\text{Stress}}\;{\text{tolerance}}\;{\text{index}} - {\text{Yp}}*{\text{Ys}}/\overline{{\text{Y}}}{\rm p}^{2}$$

whereas Yp, performance of genotypes under normal condition. Ys, performance of genotypes under stress condition. $\overline{Y}p^{2}$, mean performance of all genotypes under normal condition.

### Identification of salt stress responsive genes in wheat

Several genes have been functionally validated for salt stress tolerance in rice and wheat (Supplementary Table S2). We downloaded the protein sequences of the functionally validated salt stress responsive genes from rice genome TIGR and NCBI databases (Supplementary Table S2)^33,64^. These protein sequences were used as a query against the wheat proteome database to identify the respective wheat orthologs^11^. The chromosomal location and scaffold details of the genes were retrieved from the wheat genome database^11^. The online tools, WoLF PSORT^41^ and TargetP 1.1 serve^42^ were used to predict the sub-cellular localization of the identified salt-responsive wheat proteins.

### Identification of salt responsive *MIR* genes and their gene targets in wheat

Many miRNAs genes were functionally validated for salt stress tolerance (Supplementary Table S3). We have used known miRNAs as a query against the wheat genome and miRBase databases and identified the salt-responsive *TaMIR* gene family members for further analysis^12,24^. The duplicated *TaMIR* sequences were removed by Clustal Omega^43^. The matured *miRNA* sequences targeting the salt-responsive genes were predicted using psRNATarget server with default parameters^65,66^.

### Digital expression analysis

Tissue and developmental stage-specific expression pattern of identified wheat salt-responsive genes were studied by analysing the normalized IWGSC high quality mRNA-seq expression datasets of wheat^11^ using Genevestigator and exVIP database^12,44,67^. Wheat genome array datasets developed by Mott and Wang^46^ was used to study the salt-stress specific expression using the Genevestigator tool^44^.

### qPCR expression analysis of salt responsive genes

To validate the array expression eight salt-responsive DEGs were randomly selected. The seeds of two bread wheat varieties Kharchia65 and HD 2009 were surface sterilized with HgCl_2_ (0.1%) and grown in hydroponic conditions with full strength Hoagland solution. After fourteen days, the NaCl treatment (117 mM) was given to the wheat seedlings for 3 h and samples were collected for expression analysis. Total RNA was extracted from the root tissues using the RNA isolation kit (Qiagen). DNA was removed using the RNase-free DNase and RNA was purified using Qiagen RNeasy column. The cDNA was synthesised through the Superscript® III First Strand cDNA Synthesis kit as per manufacturer’s instructions. The qPCR was done in a reaction volume of 10 μl containing 3 μl of cDNA, 5 μl of 2 × SYBR Green Master Mix (Thermo Scientific) and 1 μl each of forward and reverse primer as per the manufacturers instructions. *Actin* gene was used as intenal reference gene for normalization. Expression analysis were performed using three biological replicates and relative expression level was computed using comparative 2^−ΔΔCt^ method^68,69^. The details of primers used in the qRT-PCR study was given in Supplementary Table S6.

### SSR mining and primer designing

The identified salt stress-responsive genes genomic sequences were used to mine candidate gene-based (cg-SSRs) and miRNA SSRs (miR-SSRs) in wheat. The BatchPrimer3 tool was used for mining SSRs. The primers were designed using the BatchPrimer3 tool from the flanking sequences of the identified microsatellite repeats^47,48^. We included di-, tri-, tetra-, peta- and hexa- nucleotide repeats; repetition of motifs less than three times were excluded. The following parameters were used for primer design: primer length 18–24 bp, melting temperature (Tm) 58–62 °C, amplicon size 100–250 bp and GC content 45–60%. Details of microsatellite repeats, SSR primers, melting temperature (Tm), amplicons size are given in Supplementary Table S7.

### Plant materials and genomic DNA extraction

A diverse panel of 54 *T. aestivum* cultivars with contrasting salt-tolerance were used in this study. These genotypes were grown at an optimum temperature of 22 ± 2 °C under a 16 h/8 h light/dark cycle in plant tissue culture room. Details of wheat genotypes are given in Supplementary Table S1a. Fresh leaf sample was collected from 54 different wheat genotypes at the seedling stage. DNA was isolated by CTAB method with little modification^70^. Leaf tissue (~ 1 g) was ground to fine powder using liquid nitrogen. Fine powder was transferred to a 50 ml Falcon tube containing 5 ml of pre-warmed CTAB DNA extraction buffer (2.0% CTAB (w/v); 0.2 M Tris-Cl, pH 8; 0.02 M of EDTA, pH 8; 1.4 M NaCl). Samples were incubated at 65 °C for 1 h. An equal volume of chloroform-isoamyl alcohol (24:1) was added to each sample at room temperature. The solution was mixed by inverting the tubes and centrifuged at 12,000 rpm for 10 min at room temperature. The aqueous phase was removed and transfered to an Eppendorf tube, 2/3rd volume of chilled isopropanol was then added. Genomic DNA was precipitated by centrifugation at 12,000 rpm for 15 min at 4 °C. Pellet was air dried and dissolved in 500 µl autoclaved double distilled H_2_O. RNase-A was added to each sample to remove RNA contamination. The quality and concentration of gDNA were checked using 0.8% agarose gel electrophoresis (Bio-Rad, USA).

### PCR amplification and PAGE

A total of 53 SSRs comprising 43 cg-SSRs and 10 miR-SSRs was designed to observe the polymorphism patterns in 54 genotypes of wheat (Supplementary Table S1a and Supplementary Table S7). All the SSR primers were amplified with gDNA isolated from 54 different genotypes of wheat as a template using a C1000 Thermal Cycler (Bio-Rad, USA). Each 25 µl PCR reaction contained 100 ng gDNA as a template, 1 × Taq DNA polymerase buffer, 2 mM MgCl_2_, 0.2 mM dNTPs mix, 1 U of Taq DNA polymerase, and 0.5 pM each of the forward and reverse primers (New England Biolabs, UK Ltd.). The PCR was optimized at an initial denaturation step of 95 °C (4 min), followed by 35 cycles of 94 °C (30 s), 55 °C (30 s), 72 °C (30 s) and a final extension step of 72 °C (10 min). The SSR PCR products were resolved in a vertical 6% non denaturing polyacrylamide gel electrophoresis (PAGE) system with 1X TAE (Tris acetate EDTA) buffer (pH 8.0). The gel was stained with ethidium bromide solution and visualized in a gel documentation system (C.B.S Scientific Co., Del Mar, CA). The composition of polyacrylamide gel included: acrylamide:bisacrylamide (29:1) (% w/v), ammonium persulfate (10% w/v) and TEMED. The electrophoresis unit, glass plates, combs and spacers, gel-sealing tape, micropipette, petroleum jelly and syringe were cleaned with 70% ethanol. The fragment size of each locus was determined by a 100 bp standard size marker (NEB). Results were confirmed by two replicate assays.

### Data analysis

The cg-SSRs and miR-SSRs based marker profiles amongst the 54 genotypes of wheat were scored for the presence (1) or absence (0) of the amplicons and a binary matrix was generated. Polymorphism information content (PIC) value of each primer pairs was calculated with the help of the formula: PIC = 1 − ∑pi^2^, where pi is equal to the frequency of the ith allele of a particular locus^71^. Genetic distance among the genotypes based on allelic data and Euclidean distance matrix were estimated using neighbour joining method in DARwin software v5.0.158^72^.

## Supplementary Information

Supplementary Table S1.

Supplementary Table S2–3.

Supplementary Table S4.

Supplementary Table S5.

Supplementary Table S6.

Supplementary Table S7.
