# Supplementary material for: Identification and development of novel salt-responsive candidate gene based SSRs (cg-SSRs) and MIR gene based SSRs (mir-SSRs) in bread wheat (Triticum aestivum)
Source: Sci Rep. 2021 Jan 26;11:2210. doi: 10.1038/s41598-021-81698-3 (PMC7838269; doi:10.1038/s41598-021-81698-3)
Supplement: Supplementary file 1 — Supplementary Table S1. [file 41598_2021_81698_MOESM1_ESM.docx]

**Identification and development of novel salt-responsive candidate gene based SSRs (cg-SSRs) and *MIR* gene based SSRs (mir-SSRs) in bread wheat (*Triticum aestivum*)**

Geetika Mehta^1#^, Senthilkumar K Muthusamy^1, 2 #^, G. P. Singh^1^, Pradeep Sharma^1,^ *

^1^Division of Crop Improvement, ICAR-Indian Institute of Wheat and Barley Research, Karnal, India

^2^Division of Crop Improvement, ICAR-Central Tuber Crops Research Institute, Thiruvananthapuram, India

^#^Equal contribution

^*^[Pradeep.Sharma@icar.gov.in](mailto:Pradeep.Sharma@icar.gov.in)

**Supplementary Table S1a** List of genotypes, pedigree details and their stress tolerance index

| **S.No.** | **Genotypes** | **Year of Release/**  **Location** | **Pedigree** | **STI** |
| --- | --- | --- | --- | --- |
|  | Kharchia65 | 1970, Durgapura | KHARCHIA LOCAL/EG 953 | 2.23 |
|  | Kharchia local | Rajasthan | Indigenous genotypes | 2.77 |
|  | KRL-1-4 | 1990, CSSRI Karnal | KHARCHIA/WL711 | 2.02 |
|  | KRL19 | 1999, CSSRI Karnal | PBW 255/KRL 1-4 | 2.14 |
|  | KRL210 | 2012, CSSRI Karnal | PBW65/2*PASTOR | 1.72 |
|  | KRL213 | 2011, CSSRI Karnal | CNDO/R143/ENTE/MEXL-2/3/Ae.Sequarrosa9TAUS0/4/WEAVER/5/28KAUZ | 1.99 |
|  | KRL99 | 2007, CSSRI Karnal | KRL-3-4/CIMK2//KRL 1-4 | 3.18 |
|  | KRL-3-4 | 2010, CSSRI Karnal | HD1982/KHARCHIA65 | 3.08 |
|  | KRL35 | 2004, CSSRI Karnal | HD2160/KRL 1-4 | 3.10 |
|  | WH157 | 1978, Hisar | NP876/S308//CNO/8156 | 2.35 |
|  | NW1067 | 2012, Faizabad | TR380-16-30614/CHAT'S' | 1.71 |
|  | HD4530 | 1979, New Delhi | TPT/MOGHK//4/PI/TML//2*TC60/3/ZENATI/BTL//WLLS | 0.30 |
|  | GW1 | 1980,Junagarh | A206/VSM//A206 | 0.40 |
|  | GW3 |  |  | 0.30 |
|  | HD2851 | 2006, New Delhi | CPAN3004/WR426/HW2007 | 1.02 |
|  | HD2009 | 1974, New Delhi | LR64A/NAI60 | 0.46 |
|  | BROOKTON |  |  | 0.67 |
|  | HD2285 | 1984, New Delhi | HD1918/HD1592/3/HD1962//E4870/K65/4/HD2160/5/HD2180 | 1.37 |
|  | C306 | 1965, Hisar | RGN/CSK3 //2*C591/3/C217/N14 //C281 | 0.92 |
|  | CBW38 | 2008, DWR | CNDO/R 143/ENTE/MEXI_2/3/Ae.SQUARROSA (TAUS)/4/WEAVER/5/2*PASTOR | 0.59 |
|  | DBW90 | 2013,DWR | HUW468/WH730 | 1.44 |
|  | DBW14 | 2002, DWR | RAJ3765/PBW343 | 0.94 |
|  | DBW17 | 2006, DWR | CMH79A.95/3*CNO79//RAJ3777 | 0.73 |
|  | GW322 | 2001, Vijapur | GW 173/GW 196 | 1.14 |
|  | HD2687 | 2005, IARI New Delhi | Gene introgressed from Sr31 Lr26  Yr9 Pm8 | 1.41 |
|  | HD2967 | 2011,New Delhi | ALD/CUC//URES/HD216OM/HD2278 | 1.66 |
|  | HD2808 | New Delhi | WH 542/DL 377-8 | 0.51 |
|  | HD2932 | 2007, New Delhi | KAUZ/STAR//HD 2643 | 0.77 |
|  | HI1500 | 2002, Indore | HW2002*2//STREMPALLI/PNC5 | 0.61 |
|  | HS240 | 1989, Shimla | AU /KAL-BB//WOP‟S‟/PAVON‟S‟ | 0.95 |
|  | HUW468 | 1999, Varanasi | CPAN-1962 / TONI // LIRA'S‟ / PRL'S' | 1.34 |
|  | HUW510 | 2001, BHU Varanasi | HD 2278 / HUW 234 // DL 230-16 | 1.37 |
|  | K7903 | 1999, Kanpur | HD 1982/K816 | 0.98 |
|  | MACS6145 | 2002, Pune | C306+LR28 | 0.52 |
|  | MACS2496 | 1991, pune | SELECTION FROM SERI ᾽S᾿ | 0.75 |
|  | MP4010 | 2001, Gwaloir | ANGOSTURA 88 | 1.39 |
|  | NI5439 | 1973, NIPHAD | REMP 80 /3* NP 710 | 0.88 |
|  | NIAW34 | 1995, NIPHAD | CNO79/PRL"S" | 1.34 |
|  | NW1014 | 1997, Faizabad | HAHN „S‟ | 1.21 |
|  | PBW550 | 2007, Ludhiana | WH594/RAJ3856//W485 | 0.41 |
|  | PBW343 | 1995, Ludhiana | ND/VG1944//KAL//BB/3/YACO'S'/4/VEE#5'S' | 1.70 |
|  | PBW590 | 2008,  Ludhiana | WH 594/RAJ3814//W 485 | 0.68 |
|  | RAJ3765 | 1995, Durgapura | HD2402/VL639 | 0.89 |
|  | UP2338 | 1994, Pantnagar | UP 368 /VL 421//UP 262 | 1.21 |
|  | UP2382 | 1998, Pantnagar | CPAN 2004 / HD 2204 | 1.00 |
|  | VL616 | 1986, Almora | SONALIKA/CPAN 1507 | 0.50 |
|  | WH730 | Hisar | CPAN 2092 / Improved Lok-1 | 1.45 |
|  | WH1080 | 2011, Hisar | 21STSAWSN151 | 1.10 |
|  | WH147 | 1978, Hisar | E 4870/C286/C273 /4/S339/PV18 | 1.53 |
|  | WR544 | 2003, Delhi | KALYANSONA/HD1999//HD2204/DW38 | 0.81 |
|  | RAJ4037 | 2003, Durgapura | DL788-2/RAJ3717 | 0.56 |
|  | RAJ4079 | 2010, Durgapura | UP 2363/WH 595 | 0.69 |
|  | RAJ4083 | 2006, Durgapura | PBW 343/UP 2442//WR 258/UP 2425 | 0.81 |
|  | RAJ4210 | Durgapura | HW 2042/L OK-1 412w/x V/HW 2042/Lok-1 | 0.61 |

*CSSRI- Central Soil Salinity Research Institute; BHU-Banaras Hindu University

**Supplementary Table S1b** Sodium and potassium concentration (%) in roots and shoot of 54 wheat genotype treated with different NaCl treatment for 14 days

| **Genotype** | **Element** | **Shoot** | | **Root** | |
| --- | --- | --- | --- | --- | --- |
|  |  | **stress** | **control** | **control** | **stress** |
| Kharchia65 | K | 5.78 | 6.23 | 1.28 | 1.32 |
|  | Na | 0.632 | 0.032 | 0.043 | 0.476 |
|  | K/Na | 9.14 | 192.94 | 29.61 | 2.78 |
| Kharchia local | K | 5.55 | 8.16 | 0.79 | 1.54 |
|  | Na | 0.540 | 0.073 | 0.028 | 0.569 |
|  | K/Na | 10.28 | 111.21 | 28.30 | 2.70 |
| KRL-1-4 | K | 7.22 | 8.09 | 1.68 | 1.31 |
|  | Na | 0.623 | 0.041 | 0.061 | 0.806 |
|  | K/Na | 11.59 | 195.22 | 27.40 | 1.62 |
| KRL19 | K | 8.58 | 7.57 | 1.76 | 1.69 |
|  | Na | 0.433 | 0.030 | 0.048 | 0.522 |
|  | K/Na | 19.80 | 254.35 | 37.01 | 3.24 |
| KRL210 | K | 5.12 | 10.15 | 2.64 | 1.53 |
|  | Na | 0.415 | 0.046 | 0.067 | 0.758 |
|  | K/Na | 12.36 | 219.43 | 39.62 | 2.02 |
| KRL213 | K | 7.17 | 9.77 | 0.96 | 1.12 |
|  | Na | 0.569 | 0.064 | 0.067 | 0.397 |
|  | K/Na | 12.59 | 151.73 | 14.32 | 2.83 |
| KRL99 | K | 7.06 | 8.73 | 1.27 | 2.01 |
|  | Na | 0.538 | 0.074 | 0.082 | 0.760 |
|  | K/Na | 13.13 | 118.49 | 15.56 | 2.64 |
| KRL-3-4 | K | 5.36 | 5.24 | 1.61 | 1.69 |
|  | Na | 0.409 | 0.035 | 0.037 | 0.533 |
|  | K/Na | 13.10 | 151.07 | 43.33 | 3.18 |
| KRL35 | K | 5.87 | 7.28 | 1.43 | 2.00 |
|  | Na | 0.500 | 0.049 | 0.054 | 0.655 |
|  | K/Na | 11.76 | 148.86 | 26.31 | 3.05 |
| WH157 | K | 4.31 | 6.37 | 1.09 | 1.05 |
|  | Na | 0.576 | 0.039 | 0.048 | 0.344 |
|  | K/Na | 7.48 | 162.35 | 22.56 | 3.04 |
| NW1067 | K | 5.43 | 8.06 | 1.91 | 1.51 |
|  | Na | 0.309 | 0.043 | 0.044 | 0.556 |
|  | K/Na | 17.55 | 188.70 | 43.62 | 2.71 |
| HD4530 | K | 2.34 | 6.57 | 3.34 | 0.97 |
|  | Na | 0.544 | 0.055 | 0.074 | 0.201 |
|  | K/Na | 4.30 | 119.31 | 45.00 | 4.83 |
| DW1 | K | 5.75 | 9.13 | 3.34 | 2.06 |
|  | Na | 0.469 | 0.045 | 0.061 | 0.497 |
|  | K/Na | 12.25 | 205.00 | 54.55 | 4.13 |
| DW3 | K | 6.46 | 7.96 | 3.45 | 1.63 |
|  | Na | 0.582 | 0.063 | 0.105 | 0.544 |
|  | K/Na | 11.11 | 125.51 | 32.87 | 3.00 |
| HD2851 | K | 6.35 | 9.15 | 1.63 | 1.66 |
|  | Na | 0.569 | 0.056 | 0.048 | 0.522 |
|  | K/Na | 11.15 | 162.47 | 33.72 | 3.18 |
| HD2009 | K | 6.39 | 9.10 | 2.65 | 2.01 |
|  | Na | 0.429 | 0.047 | 0.059 | 0.578 |
|  | K/Na | 14.87 | 193.42 | 44.78 | 3.48 |
| BROOKTON | K | 6.53 | 8.35 | 1.43 | 1.49 |
|  | Na | 0.611 | 0.035 | 0.035 | 0.419 |
|  | K/Na | 10.69 | 235.71 | 40.93 | 3.56 |
| HD2285 | K | 5.28 | 9.14 | 1.51 | 0.83 |
|  | Na | 0.662 | 0.073 | 0.038 | 0.311 |
|  | K/Na | 7.98 | 126.00 | 40.34 | 2.67 |
| C306 | K | 7.65 | 11.18 | 2.69 | 1.71 |
|  | Na | 0.484 | 0.042 | 0.058 | 0.556 |
|  | K/Na | 15.79 | 265.59 | 46.72 | 3.07 |
| CBW38 | K | 7.86 | 10.51 | 2.48 | 1.42 |
|  | Na | 0.570 | 0.038 | 0.073 | 0.718 |
|  | K/Na | 13.80 | 273.87 | 34.18 | 1.97 |
| DBW90 | K | 5.79 | 9.27 | 1.88 | 1.43 |
|  | Na | 0.297 | 0.032 | 0.061 | 0.443 |
|  | K/Na | 19.47 | 287.06 | 30.71 | 3.22 |
| DBW14 | K | 9.23 | 5.88 | 2.07 | 1.47 |
|  | Na | 0.352 | 0.028 | 0.051 | 0.621 |
|  | K/Na | 26.24 | 206.81 | 40.71 | 2.36 |
| DBW17 | K | 4.46 | 11.28 | 2.07 | 1.09 |
|  | Na | 0.315 | 0.035 | 0.055 | 0.398 |
|  | K/Na | 14.14 | 318.21 | 37.56 | 2.73 |
| GW322 | K | 7.34 | 10.36 | 1.88 | 1.39 |
|  | Na | 0.337 | 0.041 | 0.050 | 0.436 |
|  | K/Na | 21.79 | 253.64 | 37.59 | 3.20 |
| HD2687 | K | 6.11 | 10.43 | 1.80 | 1.84 |
|  | Na | 0.386 | 0.048 | 0.052 | 0.450 |
|  | K/Na | 15.84 | 216.15 | 34.63 | 4.08 |
| HD2967 | K | 9.32 | 8.91 | 1.61 | 1.51 |
|  | Na | 0.377 | 0.034 | 0.048 | 0.436 |
|  | K/Na | 24.74 | 261.82 | 33.42 | 3.47 |
| HD2808 | K | 7.24 | 9.39 | 1.80 | 1.65 |
|  | Na | 0.427 | 0.040 | 0.044 | 0.524 |
|  | K/Na | 16.96 | 233.54 | 41.30 | 3.14 |
| HD2932 | K | 6.97 | 6.02 | 1.96 | 1.46 |
|  | Na | 0.575 | 0.025 | 0.037 | 0.459 |
|  | K/Na | 12.13 | 242.79 | 52.36 | 3.18 |
| HI1500 | K | 1.68 | 6.11 | 1.83 | 1.89 |
|  | Na | 0.429 | 0.032 | 0.062 | 0.550 |
|  | K/Na | 3.90 | 193.93 | 29.59 | 3.43 |
| HS240 | K | 1.97 | 9.46 | 1.78 | 1.48 |
|  | Na | 0.272 | 0.039 | 0.062 | 0.352 |
|  | K/Na | 7.26 | 244.92 | 28.80 | 4.21 |
| HUW468 | K | 8.92 | 10.63 | 1.87 | 1.66 |
|  | Na | 0.342 | 0.045 | 0.062 | 0.427 |
|  | K/Na | 26.11 | 234.40 | 30.10 | 3.89 |
| HUW510 | K | 9.80 | 9.84 | 1.81 | 1.58 |
|  | Na | 0.386 | 0.064 | 0.049 | 0.408 |
|  | K/Na | 25.38 | 154.37 | 36.58 | 3.87 |
| K7903 | K | 5.70 | 9.69 | 1.75 | 0.77 |
|  | Na | 0.456 | 0.038 | 0.049 | 0.290 |
|  | K/Na | 12.50 | 256.72 | 35.97 | 2.67 |
| MACS6145 | K | 5.68 | 9.91 | 3.11 | 1.71 |
|  | Na | 0.866 | 0.052 | 0.091 | 0.547 |
|  | K/Na | 6.56 | 190.47 | 34.33 | 3.13 |
| MACS2496 | K | 7.75 | 9.85 | 2.27 | 1.82 |
|  | Na | 0.285 | 0.037 | 0.057 | 0.364 |
|  | K/Na | 27.20 | 264.76 | 39.62 | 5.00 |
| MP4010 | K | 5.28 | 9.29 | 1.75 | 1.12 |
|  | Na | 0.525 | 0.031 | 0.042 | 0.305 |
|  | K/Na | 10.05 | 295.38 | 41.62 | 3.67 |
| NI5439 | K | 6.12 | 10.99 | 2.33 | 1.17 |
|  | Na | 0.385 | 0.046 | 0.071 | 0.284 |
|  | K/Na | 15.89 | 236.80 | 32.72 | 4.11 |
| NIAW34 | K | 5.55 | 6.23 | 1.97 | 1.38 |
|  | Na | 0.486 | 0.024 | 0.056 | 0.378 |
|  | K/Na | 11.42 | 258.95 | 35.45 | 3.66 |
| NW1014 | K | 9.11 | 11.40 | 1.78 | 1.74 |
|  | Na | 0.387 | 0.053 | 0.045 | 0.490 |
|  | K/Na | 23.53 | 216.71 | 39.45 | 3.54 |
| PBW550 | K | 5.28 | 8.02 | 2.09 | 2.27 |
|  | Na | 0.576 | 0.044 | 0.051 | 0.370 |
|  | K/Na | 9.16 | 181.64 | 41.35 | 6.15 |
| PBW343 | K | 6.42 | 11.05 | 2.05 | 1.18 |
|  | Na | 0.198 | 0.046 | 0.048 | 0.467 |
|  | K/Na | 32.44 | 239.18 | 43.06 | 2.52 |
| PBW590 | K | 4.34 | 11.89 | 1.52 | 1.58 |
|  | Na | 0.439 | 0.054 | 0.028 | 0.318 |
|  | K/Na | 9.89 | 218.37 | 53.41 | 4.98 |
| RAJ3765 | K | 6.45 | 9.73 | 1.89 | 1.52 |
|  | Na | 0.555 | 0.048 | 0.041 | 0.325 |
|  | K/Na | 11.62 | 201.54 | 46.21 | 4.67 |
| UP2338 | K | 8.11 | 12.03 | 2.21 | 1.81 |
|  | Na | 0.343 | 0.053 | 0.049 | 0.363 |
|  | K/Na | 23.66 | 228.46 | 45.15 | 5.00 |
| UP2382 | K | 6.13 | 11.62 | 1.79 | 1.83 |
|  | Na | 0.265 | 0.048 | 0.073 | 0.538 |
|  | K/Na | 23.12 | 243.90 | 24.65 | 3.39 |
| VL616 | K | 8.07 | 10.25 | 2.81 | 3.23 |
|  | Na | 0.401 | 0.027 | 0.068 | 1.137 |
|  | K/Na | 20.13 | 376.74 | 41.35 | 2.84 |
| WH730 | K | 6.87 | 7.31 | 1.78 | 1.92 |
|  | Na | 0.405 | 0.041 | 0.034 | 0.349 |
|  | K/Na | 16.97 | 179.09 | 52.07 | 5.48 |
| WH1080 | K | 5.13 | 6.41 | 1.83 | 1.72 |
|  | Na | 0.329 | 0.036 | 0.034 | 0.392 |
|  | K/Na | 15.58 | 180.00 | 54.11 | 4.38 |
| WH147 | K | 5.43 | 10.94 | 1.85 | 1.93 |
|  | Na | 0.344 | 0.041 | 0.067 | 0.588 |
|  | K/Na | 15.81 | 270.00 | 27.57 | 3.28 |
| WR544 (Pusa gold) | K | 4.81 | 8.89 | 1.88 | 1.17 |
|  | Na | 0.653 | 0.041 | 0.091 | 0.248 |
|  | K/Na | 7.37 | 216.00 | 20.61 | 4.71 |
| RAJ4037 | K | 5.04 | 11.50 | 2.51 | 1.62 |
|  | Na | 0.321 | 0.034 | 0.043 | 0.292 |
|  | K/Na | 15.74 | 334.62 | 57.80 | 5.55 |
| RAJ4079 | K | 4.74 | 6.28 | 1.18 | 1.34 |
|  | Na | 0.362 | 0.062 | 0.042 | 0.242 |
|  | K/Na | 13.07 | 101.09 | 28.10 | 5.53 |
| RAJ4083 | K | 8.42 | 8.26 | 2.63 | 1.68 |
|  | Na | 0.330 | 0.071 | 0.128 | 0.343 |
|  | K/Na | 25.53 | 116.50 | 20.47 | 4.89 |
| RAJ4210 | K | 7.30 | 8.02 | 2.35 | 2.58 |
|  | Na | 0.615 | 0.077 | 0.102 | 0.678 |
|  | K/Na | 11.87 | 103.59 | 23.11 | 3.81 |
