# Supplementary material for: Identification and development of novel salt-responsive candidate gene based SSRs (cg-SSRs) and MIR gene based SSRs (mir-SSRs) in bread wheat (Triticum aestivum)
Source: Sci Rep. 2021 Jan 26;11:2210. doi: 10.1038/s41598-021-81698-3 (PMC7838269; doi:10.1038/s41598-021-81698-3)
Supplement: Supplementary file 2 — Supplementary Table S2–3. [file 41598_2021_81698_MOESM2_ESM.docx]

**Identification and development of novel salt-responsive candidate gene based SSRs (cg-SSRs) and *MIR* gene based SSRs (mir-SSRs) in bread wheat (*Triticum aestivum*)**

Geetika Mehta^1#^, Senthilkumar K Muthusamy^1, 2 #^, G. P. Singh^1^, Pradeep Sharma^1,^ *

^1^Division of Crop Improvement, ICAR-Indian Institute of Wheat and Barley Research, Karnal, India

^2^Division of Crop Improvement, ICAR-Central Tuber Crops Research Institute, Thiruvananthapuram, India ^#^Equal contribution

**Supplementary Table S2** List of functionally validated rice and wheat salt stress-responsive genes with their LOC number and function. TF- transcription factor

| **S. No** | **Gene name** | **Locus ID** | **Function** | **Reference** |
| --- | --- | --- | --- | --- |
| 1 | *OsHKT1* | LOC_Os04g51820.1 | Transporter | (Wang et al., 2015a) |
| 2 | *OsMYB* | LOC_Os09g12770.1 | TF | (Wang et al., 2015a) |
| 3 | *H+- ATPase* | LOC_Os12g44150.1 | Transporter | (Zhang et al., 1999) |
| 4 | *ONAC045* | LOC_Os11g03370 | TF | (Zheng et al., 2009) |
| 5 | *OrbHLH001* | LOC_Os01g70310.1 | TF | (Li et al., 2010b) |
| 6 | *OrbHLH2* | LOC_Os11g32100 | TF | (Zhou et al., 2009) |
| 7 | *OrbHLH2* | LOC_Os11g32100 | TF | (Zhou et al., 2009) |
| 8 | *OsABCG5* | LOC_Os03g17350 | Transporter | (Matsuda et al., 2014) |
| 9 | *OsABP* | LOC_Os06g33520 | Helicase | (Macovei et al., 2012; Macovei and Tuteja, 2012) |
| 10 | *OsACA6* | LOC_Os04g51610 | Ca^2+^ ATPase | (Huda et al., 2013) |
| 11 | *OsAKT1* | LOC_Os01g45990 | Ion Transporter | (Fuchs et al., 2005) |
| 12 | *OsAOX1a* | LOC_Os04g51150.1 | Electron Transporter | (Ohtsu et al. 2002) |
| 13 | *OsAOX1b* | LOC_Os04g51160 | Electron Transporter | (Senadheera et al., 2009) |
| 14 | *OsAP21* | LOC_Os01g10370 | TF | (Jin et al., 2013) |
| 15 | *OsAPX4* | LOC_Os08g43560.1 | Anti oxidation Enzyme | (Guan et al., 2013) |
| 16 | *OsAPX7* | LOC_Os04g35520 | Anti oxidation Enzyme | (Teixeira et al., 2006) |
| 17 | *OsAPXb* | LOC_Os07g49400.2 | Anti oxidation Enzyme | (Lu et al., 2007) |
| 18 | *OsBADH1* | LOC_Os04g39020.1 | Osmoprotection | (Hasthanasombut et al., 2010) |
| 19 | *OsBIERF3* | LOC_Os02g43790 | TF | (Cao et al., 2005) |
| 20 | *OsBIHD1* | LOC_Os03g47740 | TF | (Luo et al., 2005) |
| 21 | *OsbZIP71* | LOC_Os09g13570 | TF | (Liu et al., 2014a) |
| 22 | *OsC3H33* | LOC_Os05g03760 | TF | (Jamil et al., 2010) |
| 23 | *OsC3H37* | LOC_Os05g45020 | TF | (Jamil et al., 2010) |
| 24 | *OsC3H50* | LOC_Os07g38090 | TF | (Jamil et al., 2010) |
| 25 | *OsCA1* | LOC_Os01g45274.1 | Enzyme carbonic anahydrase | (Yu et al., 2007) |
| 26 | *OsCam1-1* | LOC_Os03g20370 | Signalling | (Saeng-ngam et al., 2012) |
| 27 | *OsCAX* | LOC_Os02g04630 | Functional, Ion-channel | (Senadheera et al., 2009) |
| 28 | *OsCBSX4* | LOC_Os03g52690 | Regulatory, Adenosine binding | (Singh et al., 2012) |
| 29 | *OsCDKC* | LOC_Os01g72790.1 | Regulatory, Kinase | (Huang et al., 2008c) |
| 30 | *OsCIPK15* | LOC_Os11g02240 | Signalling | (Xiang et al., 2007) |
| 31 | *OsCLC-1* | LOC_Os02g35190.2 | Functional, Ion transport | (Nakamura et al., 2006) |
| 32 | *OsCML11* | LOC_Os01g32120 | Regulatory, Ca2+-binding | (Chinpongpanich et al., 2012) |
| 33 | *OsCML31* | LOC_Os01g72530.1 | Regulatory, Ca2+-binding | (Xu et al., 2011) |
| 34 | *OsCML5* | LOC_Os12g41110 | Signalling | (Chinpongpanich et al., 2012) |
| 35 | *OsCML8* | LOC_Os10g25010 | Regulatory, Ca2+-binding | (Chinpongpanich et al., 2012) |
| 36 | *OsCOIN* | LOC_Os01g01420.1 | RING finger TF | (Liu et al., 2007b) |
| 37 | *OsCPK17* | LOC_Os07g06740.2 | Signalling, Kinase | (Wan et al., 2007) |
| 38 | *OsCPK21* | LOC_Os08g42740.3 | Signalling, Kinase | (Asano et al., 2011) |
| 39 | *OsCyP20–2* | LOC_Os05g01270 | Functional, Protein folding | (Kim et al., 2012) |
| 40 | *OsDBH1* | LOC_Os04g40970 | DEAD- box, ATP dependent RNA helicase | (Macovei and Tuteja, 2012) |
| 41 | *OsDREB1A* | LOC_Os09g35030 | DNA binding protein | (Huang et al., 2012a) |
| 42 | *OsDREB1F* | LOC_Os01g73770.1 | TF | (Wang et al., 2008) |
| 43 | *OsDSM1* | LOC_Os02g50970 | Signalling, Kinase | (Ning et al., 2010) |
| 44 | *OsDST* | LOC_Os03g57240 | TF, H2O2-homeostasis | (Huang et al., 2009) |
| 45 | *OsECS* | LOC_Os05g03820.3 | Functional, antioxidation | (Choe et al., 2013) |
| 46 | *OsERF92-2* | LOC_Os01g54890 | TF | (Liu et al., 2012) |
| 47 | *OsGGT* | LOC_Os10g40640.1 | Functional, Glycosylation | (Qi et al., 2005) |
| 48 | *OsglyII* | LOC_Os09g34100.1 | Functional, glyoxalate-pathway | (Wani and Gosal, 2011) |
| 49 | *OsGMST1* | LOC_Os02g17500 | Functional, Sugar transporter | (Cao et al., 2011) |
| 50 | *OsGR3* | LOC_Os10g28000 | Functional, Reduces GSSG | (Wu et al., 2013) |
| 51 | *OsHAP2E* | LOC_Os03g29760 | TF | (Alam et al., 2015) |
| 52 | *OsHBP1b* | LOC_Os01g17260 | TF | (Lakra et al., 2015) |
| 53 | *OsHKT1;4* | LOC_Os04g51830 | Na+ transporter | (Hauser and Horie, 2010) |
| 54 | *OsHKT8* | LOC_Os01g20160 | Functional, Ion transport | (Rus et al., 2005) |
| 55 | *OsHsfA7* | LOC_Os01g39020 | TF, Chaperon | (Liu et al., 2013a) |
| 56 | *OsHsfC1b* | LOC_Os01g53220 | TF, chaperon | (Schmidt et al., 2012) |
| 57 | *OsHsp90* | LOC_Os06g50300.1 | Functional, Heat shock |  |
| 58 | *OsiSAP8* | LOC_Os06g41010.1 | Signalling, protein binding | (Kanneganti and Gupta, 2008) |
| 59 | *OsJAZ9* | LOC_Os03g08310 | Transcription regulation | (Wu et al., 2015) |
| 60 | *OsMAPK44* | LOC_Os08g06060.1 | Signalling, Kinase | (Jeong et al., 2006) |
| 61 | *OsMGD* | LOC_Os02g55910.1 | Functional, Lipid Biosynthesis | (Wang et al., 2014c) |
| 62 | *OsMIOX* | LOC_Os06g36560.1 | Balances the concentration of myo-inositol | (Duan et al., 2012) |
| 63 | *OsMSRB* | LOC_Os06g27760 | Functional, antioxidation | (Guo et al., 2009) |
| 64 | *OsMSRMK3* | LOC_Os06g48590.1 | Signalling, Kinase | (Agrawal et al., 2003) |
| 65 | *OSMT1e-P* | LOC_Os11g47809.1 | Cysteine-rich, metal binding | (Kumar et al., 2012) |
| 66 | *OsMyb2* | LOC_Os03g20090 | TF | (Yang et al., 2012) |
| 67 | *OsMyb3R-2* | LOC_Os01g62410 | TF | (Dai et al., 2007) |
| 68 | *Osmyb4* | LOC_Os04g43680.1 | TF | (Liu et al., 2014a) |
| 69 | *OsMYB48-1* | LOC_Os01g74410 | TF | (Xiong et al., 2014) |
| 70 | *OsNAC5* | LOC_Os11g08210 | TF | (Takasaki et al., 2010) |
| 71 | *OsNAC6* | LOC_Os01g66120 | TF | (Nakashima et al., 2007) |
| 72 | *OsNOA1* | LOC_Os02g01440 | Functional, NO synthesis | (Qiao et al., 2009) |
| 73 | *OsOXHS2* | LOC_Os01g03570 | Regulatory, protein-protein interaction | (Qin et al., 2009) |
| 74 | *OsP5CR* | LOC_Os01g71990 | Functional, Osmoprotection | (Sripinyowanich et al., 2013) |
| 75 | *OsPEX11-1* | LOC_Os03g02590 | Functional, peroxisomal biogenesis | (Nayidu et al., 2008) |
| 76 | *OsPOP5* | LOC_Os02g18850 | Serine peptidases | (Tan et al., 2013) |
| 77 | *OsPUB15* | LOC_Os08g01900 | Functional, ubiquitination | (Park et al., 2011) |
| 78 | *Osr40cl* | LOC_Os03g21040.2 | ABA responsive protein | (Moons et al., 1997) |
| 79 | *OsRab7* | LOC_Os05g44050 | Signalling GTP binding | (Nahm et al., 2003) |
| 80 | *OsRacB* | LOC_Os02g02840.1 | Signalling, GTPase | (Luo et al., 2006) |
| 81 | *OsrgMT* | LOC_Os11g47809 | Metallothionein protein | (Jin et al., 2014) |
| 82 | *OsRINO1* | LOC_Os03g09250 | Myoinositol synthesis | (Kusuda et al., 2015) |
| 83 | *OsSAMDC* | LOC_Os04g42090.4 | Functional, polyamine biosynthesis | (Roy and Wu, 2002) |
| 84 | *OsSDIR1* | LOC_Os03g16570.2 | Functional, ubiquitination | (Gao et al., 2011b) |
| 85 | *OsSERF1* | LOC_Os05g34730 | TF, Inhibition of MAPK cascade | (Schmidt et al., 2013) |
| 86 | *OsSIK1* | LOC_Os06g03970 | Signalling, Kinase | (Ouyang et al., 2010) |
| 87 | *OsSKC1* | LOC_Os01g20160.1 | Functional, ion-transporter | (Ren et al., 2005) |
| 88 | *OsSKIPa* | LOC_Os02g52250 | Regulatory, Spliceosome component | (Hou et al., 2009) |
| 89 | *OsSNAC1* | LOC_Os03g60080 | TF | (Saad et al., 2013) |
| 90 | *OsSNAC2* | LOC_Os01g66120 | TF | (Hu et al., 2008) |
| 91 | *OsSOS2* | LOC_Os06g40370 | Signalling, Kinase | (Kumar et al., 2009) |
| 92 | *OsSRWD2* | LOC_Os02g48964 | Regulatory, Chromatin modification,Transcription | (Huang et al., 2008a) |
| 93 | *OsSRWD3* | LOC_Os06g07540 | Regulatory, Chromatin modification,Transcription | (Huang et al., 2008a) |
| 94 | *OsSRWD4* | LOC_Os08g31560 | Regulatory, Chromatin modification,Transcription | (Huang et al., 2008a) |
| 95 | *OsSRWD5* | LOC_Os03g26870 | Regulatory, Chromatin modification,Transcription | (Huang et al., 2008a) |
| 96 | *OsSRZ1* | LOC_Os02g10920.4 | Regulatory, Splicing | (Huang et al., 2008b) |
| 97 | *OsTIFY11a* | LOC_Os03g08310 | Regulatory | (Ye et al., 2009) |
| 98 | *OsTOP6A3* | LOC_Os03g17610.1 | Functional, Topoisomerase | (Jain et al., 2006) |
| 99 | *OsTPC1* | LOC_Os01g48680 | Functional, Ion-channel | (Senadheera et al., 2009) |
| 100 | *OsTPS1* | LOC_Os05g44210 | Functional, Trehalose biosynthesis | (Li et al., 2011) |
| 101 | *OsTZF1* | LOC_Os05g10670 | TF | (Jan et al., 2013) |
| 102 | *OsUGE-1* | LOC_Os05g51670.1 | Functional, nucleotide sugar interconversion | (Liu et al., 2007a) |
| 103 | *OsWNK1* | LOC_Os07g38530.1 | Kinase | (Kumar et al., 2011) |
| 104 | *OsWRKY13* | LOC_Os01g54600 | TF | (Qiu et al., 2007) |
| 105 | *OsWRKY45* | LOC_Os05g25770 | TF | (Tao et al., 2011) |
| 106 | *OsZFP182* | LOC_Os03g60560 | TF | (Huang et al., 2012a) |
| 107 | *OsZFP252* | LOC_Os12g41660 | TF | (Xu et al., 2008a) |
| 108 | *TmHKT8* | DQ646332.1 | Na+ transporter | (James et al., 2011) |
| 109 | *TaSC* | AY956330 | Signaling, transmembrane protein | (Huang et al., 2012c) |
| 110 | *TaNIP* | DQ530420.1 | Aquaporin | (Gao et al., 2010b) |
| 111 | *TaPIMP1* | EF587267.1 | TF | (Liu et al., 2011) |
| 112 | *TaSRG* | DQ672342 | TF | (He et al., 2011) |
| 113 | *TaMYBsdu1* | BT008981 | TF | (Rahaie et al., 2010) |
| 114 | *TaNAC29* | C116E5668.1 | TF | (Huang et al., 2015) |
| 115 | *TaNAC67* | KF646593 | TF | (Mao et al., 2014) |
| 116 | *TaMYB33* | JN584645 | TF | (Qin et al., 2012) |
| 117 | *TaNAC47* | KT345698 | TF | (Zhang et al., 2016b) |
| 118 | *TaABL1* | BJ267580 | TF | (Xu et al., 2014) |
| 119 | *TaWRKY19* | EU665430 | TF | (Niu et al., 2012) |
| 120 | *TaWRKY2* | EU665425 | TF | (Niu et al., 2012) |
| 121 | *TaERF3* | EF570122 | TF | (Rong et al., 2014) |
| 122 | *TaERF4* | JX014257 | TF | (Dong et al., 2012) |
| 123 | *TaSnRK2.8* | KR611569.1 | Signalling, Kinase | (Zhang et al., 2010) |
| 124 | *TaSnRK2.7* | KR736351.1 | Signalling, Kinase | (Zhang et al., 2011) |
| 125 | *TaSnRK2.4* | GQ384359.1 | Signalling, Kinase | (Mao et al., 2010) |
| 126 | *TaAQP8* | HQ650110 | Aquaporin | (Hu et al., 2012) |
| 127 | *TaCIPK29* | JX243013 | CBL-interacting protein kinase | (Deng et al., 2013a) |
| 128 | *HvHKT2;1* | DQ015706.1 | Na+ transporter | (Mian et al., 2011) |
| 129 | *TaWRKY10* | HQ700327 | TF | (Wang et al., 2013a) |
| 130 | *TaWRKY44* | KR827395 | TF | (Wang et al., 2015b) |
| 131 | *TaPP2C1* | HQ287800 | Protein phosphatases | (Hu et al., 2015) |
| 132 | *TaOPR1* | JQ409278 | Oxophytodienoatereductase | (Dong et al., 2013) |
| 133 | *TaCHP* | GQ379226 | TF | (Li et al., 2010a) |
| 134 | *TaDi19A* | FJ795369 | TF | (Li et al., 2010c) |
| 135 | *TdSHN1* | DQ334410 | TF | (Djemal and Khoudi, 2015) |
| 136 | *TaAIDFa* | AY781361 | TF | (Xu et al., 2008b) |
| 137 | *TaERF1* | AY781352 | TF | (Makhloufi et al., 2014) |
| 138 | *TaMAPK1* | AY881102 | TF | (Makhloufi et al., 2014) |
| 139 | *TaCIPK14* | JX879754.1 | Kinase | (Deng et al., 2013b) |
| 140 | *TaCRT1* | AY836753 | Ca^2+^-binding protein | (Xiang et al., 2015) |
| 141 | *TaEXPB23* | AY260547 | Expansins | (Han et al., 2012) |
| 142 | *TaEXPA2* | AY589584.1 | Expansins | (Chen et al., 2017) |
| 143 | *TaGly I* | AJ243528.1 | Glyoxalase | (Lin et al., 2010) |
| 144 | *TaNAS-D* | KC700308 | Nicotianamine synthase | (Han et al., 2016) |
| 145 | *TNHXS1* | AY461512.1 | Transporter | (Gouiaa et al., 2012) |
| 146 | *TVP1* | EU255237.1 | H^+^-pyrophosphatase | (Gouiaa et al., 2012) |
| 147 | *SR3 WRSI5* | AY549888 | protease inhibitor | (Shan et al., 2008) |
| 148 | *PI4K* | KC357689.1 | Kinase | (Liu et al., 2013b) |
| 149 | *TaGBF1* | KT225464.1 | TF | (Sun et al., 2015) |
| 150 | *TaMYB73* | JN969051 | TF | (He et al., 2012) |
| 151 | *W69* | KF031945 | glutathione peroxidase | (Zhai et al., 2013) |
| 152 | *W106* | KF031946 | glutathione peroxidase | (Zhai et al., 2013) |
| 153 | *TabZIP60* | KJ562868.1 | TF | (Zhang et al., 2015) |
| 154 | *TaNAC2* | AAU08786 | TF | (Mao et al., 2012) |
| 155 | *TaNHX3* | tplb0011k19 | Transporter | (Lu et al., 2014) |
| 156 | *TaPLDα* | FN377812 | phospholipase D | (Wang et al., 2014b) |
| 157 | *TaNHX2* | AY040246.2 | Transporter | (Zhang et al., 2014c) |
| 158 | *TaSOD2* | KP322572 | superoxide dismutase | (Wang et al., 2016) |
| 159 | *TMKP1* | EU502843 | MAP kinase phosphatase | (Zaidi et al., 2016) |
| 160 | *Ta-sro1* | JN202574 | Poly(ADP ribose) polymerase | (Liu et al., 2014b) |
| 161 | *TaCIPK25* | KJ561795 | CBL-interacting protein kinase | (Jin et al., 2016) |
| 162 | *TaWRKY93* | JX679079 | TF | (Qin et al., 2015) |
| 163 | *TaSP* | KF307326 | Unknown function | (Ma et al., 2015) |
| 164 | *TaST* | BQ169774 | Unknown function | (Huang et al., 2012b) |
| 165 | *Ta-UnP* | ADR63289 | Unknown function | (Liang et al., 2014) |
| 166 | *TaSRK2C1* | TC368696 | SNF1-Related Protein Kinase | (Du et al., 2013b) |
| 167 | *TaSK5* | AB281487 | kinase | (Christov et al., 2014) |
| 168 | *TaSIP* | HM205107 | Unknown function | (Du et al., 2013a) |
| 169 | *TaWRKY79* | JX047374 | TF | (Qin et al., 2013) |
| 170 | *TaSRG* | DQ672342 | TF | (He et al., 2011) |
| 171 | *TaSST* | ACH97119 | Unknown function | (Li et al., 2016) |
| 172 | *TabHLH1* | TC307165 | TF | (Yang et al., 2016) |
| 173 | *TaACO1* | AGT79999 | aminocyclopropane-1-carboxylate oxidase | (Chen et al., 2014a) |
| 174 | *TaMYB30-B* | JF951913 | TF | (Zhang et al., 2012a) |
| 175 | *TaMYB19* | JF951903 | TF | (Zhang et al., 2014b) |
| 176 | *TaMYB56-B* | JF951939 | TF | (Zhang et al., 2012b) |
| 177 | *TaMYB3R1* | HQ236494 | TF | (Cai et al., 2015) |
| 178 | *SbNADP-ME* |  | Malate Dehydrogenase | (Guo et al., 2018) |
| 179 | *SbVPPase* | GQ469975.1 | H(+)-pyrophosphatase | (Anjaneyulu et al., 2014) |
| 180 | *Sb06PPO1* | KJ425574 | polyphenol oxidase | (Yan et al., 2017) |
| 181 | *SbAP37* | JF714972 | TF | (Parveda et al., 2017) |
| 182 | *SbHKT1;4* | Sb06g027900 | Transporter | (Wang et al., 2014d) |
| 183 | *OsGR3* | AK108799 | Glutathione reductases | (Wu et al., 2013) |
| 184 | *ZmCHB101* |  | chromatin remodeler | (Yu et al., 2018) |
| 185 | *ZmPIP1;1* | X82633 | plasma membrane intrinsic protein | (Zhou et al., 2018) |
| 186 | *ZmWRKY17* | EU966905 | TF | (Cai et al., 2017) |
| 187 | *Zmhdz10* | JX514832 | TF | (Zhao et al., 2014) |
| 188 | *ZmNAC55* | AFW67212 | TF | (Mao et al., 2016) |
| 189 | *ZmbZIP72* | AK065873 | TF | (Ying et al., 2012) |
| 190 | *ABP9* | NM_001197011 | TF | (Wang et al., 2017) |
| 191 | *ZmCBL9* | GRMZM2G015324 | Regulatory, Ca2+-binding | (Zhang et al., 2016a) |
| 192 | *ZmVPP5* | NP_001140455 | Vacuole H+‐PPases | (Sun et al., 2016) |
| 193 | *ZmCBL4* | GRMZM2G001221 | Regulatory, Ca2+-binding | (Zhang et al., 2016a) |
| 194 | *ZmSKD1* | AY105155 | ATPase | (Xia et al., 2013) |
| 195 | *ZmMKK1* |  | kinase | (Cai et al., 2014) |
| 196 | *ZmCIPK21* | NM_00154244 | kinase | (Chen et al., 2014b) |
| 197 | *ZmMPK5* | EU965114 | kinase | (Zhang et al., 2014a) |
| 198 | *TSRF1* | At3g23240 | TF | (Wang et al., 2013b) |
| 199 | *PMP3* | EU364580 | Plasma membrane protein 3 | (Fu et al., 2012) |
| 200 | *ZmSIMK1* | AY433815 | kinase | (Gu et al., 2010) |

**Supplementary Table S3** List of salt stress-responsive *MIR* genes

| **Gene name** | **Crop** | **Reference** |
| --- | --- | --- |
| *miR156* | Arabidopsis | (Liu et al., 2008) |
| *miR158* | Arabidopsis | (Liu et al., 2008) |
| *miR159* | Wheat | (Wang et al., 2014a) |
| *miR160* | Wheat | (Lu et al., 2011) |
| *miR165* | Wheat | (Wang et al., 2014a) |
| *miR167* | Arabidopsis | (Liu et al., 2008) |
| *miR168* | Arabidopsis | (Liu et al., 2008) |
| *miR169* | Arabidopsis | (Kruszka et al., 2012) |
| *miR169* | Rice | (Zhao et al., 2009) |
| *miR171* | Arabidopsis | (Liu et al., 2008) |
| *miR172* | Wheat | (Gupta et al., 2014) |
| *miR319* | Arabidopsis | (Liu et al., 2008) |
| *miR393* | Rice | (Gao et al., 2011a) |
| *miR393* | Wheat | (Wang et al., 2014a) |
| *miR394* | Arabidopsis | (Song et al., 2013) |
| *miR395* | Arabidopsis | (Kim et al., 2010b) |
| *miR396* | Rice | (Gao et al., 2010a) |
| *miR396* | Arabidopsis | (Liu et al., 2008) |
| *miR402* | Arabidopsis | (Kim et al., 2010a) |
| *miR417* | Arabidopsis | (Jung and Kang, 2007) |

**References**

Agrawal, G. K., Agrawal, S. K., Shibato, J., Iwahashi, H., and Rakwal, R. (2003). Novel rice MAP kinases OsMSRMK3 and OsWJUMK1 involved in encountering diverse environmental stresses and developmental regulation. *Biochem. Biophys. Res. Commun.* 300, 775–83. Available at: http://www.ncbi.nlm.nih.gov/pubmed/12507518

Alam, M. M., Tanaka, T., Nakamura, H., Ichikawa, H., Kobayashi, K., Yaeno, T., Yamaoka, N., Shimomoto, K., Takayama, K., Nishina, H., et al. (2015). Overexpression of a rice heme activator protein gene (OsHAP2E) confers resistance to pathogens, salinity and drought, and increases photosynthesis and tiller number. *Plant Biotechnol. J.* 13, 85–96. doi:10.1111/pbi.12239.

Anjaneyulu, E., Reddy, P. S., Sunita, M. S., Kishor, P. B. K., and Meriga, B. (2014). Salt tolerance and activity of antioxidative enzymes of transgenic finger millet overexpressing a vacuolar H+-pyrophosphatase gene (SbVPPase) from Sorghum bicolor. *J. Plant Physiol.* 171, 789–798. doi:10.1016/j.jplph.2014.02.001.

Asano, T., Hakata, M., Nakamura, H., Aoki, N., Komatsu, S., Ichikawa, H., Hirochika, H., and Ohsugi, R. (2011). Functional characterisation of OsCPK21, a calcium-dependent protein kinase that confers salt tolerance in rice. *Plant Mol. Biol.* 75, 179–91. doi:10.1007/s11103-010-9717-1.

Cai, G., Wang, G., Wang, L., Liu, Y., Pan, J., and Li, D. (2014). A maize mitogen-activated protein kinase kinase, ZmMKK1, positively regulated the salt and drought tolerance in transgenic Arabidopsis. *J. Plant Physiol.* 171, 1003–1016. doi:10.1016/j.jplph.2014.02.012.

Cai, H., Tian, S., Dong, H., and Guo, C. (2015). Pleiotropic effects of TaMYB3R1 on plant development and response to osmotic stress in transgenic Arabidopsis. *Gene* 558, 227–234. doi:10.1016/j.gene.2014.12.066.

Cai, R., Dai, W., Zhang, C., Wang, Y., Wu, M., Zhao, Y., Ma, Q., Xiang, Y., and Cheng, B. (2017). The maize WRKY transcription factor ZmWRKY17 negatively regulates salt stress tolerance in transgenic Arabidopsis plants. *Planta* 246, 1215–1231. doi:10.1007/s00425-017-2766-9.

Cao, H., Guo, S., Xu, Y., Jiang, K., Jones, A. M., and Chong, K. (2011). Reduced expression of a gene encoding a Golgi localized monosaccharide transporter (OsGMST1) confers hypersensitivity to salt in rice (Oryza sativa). *J. Exp. Bot.* 62, 4595–604. doi:10.1093/jxb/err178.

Cao, Y., Wu, Y., Zheng, Z., and Song, F. (2005). Overexpression of the rice EREBP-like gene OsBIERF3 enhances disease resistance and salt tolerance in transgenic tobacco. *Physiol. Mol. Plant Pathol.* 67, 202–211. doi:10.1016/j.pmpp.2006.01.004.

Chen, D., Ma, X., Li, C., Zhang, W., Xia, G., and Wang, M. (2014a). A wheat aminocyclopropane-1-carboxylate oxidase gene, TaACO1, negatively regulates salinity stress in Arabidopsis thaliana. *Plant Cell Rep.* 33, 1815–1827. doi:10.1007/s00299-014-1659-7.

Chen, X., Huang, Q., Zhang, F., Wang, B., Wang, J., and Zheng, J. (2014b). ZmCIPK21, a Maize CBL-Interacting Kinase, Enhances Salt Stress Tolerance in Arabidopsis Thaliana. *Int. J. Mol. Sci.* 15, 14819–14834. doi:10.3390/ijms150814819.

Chen, Y., Han, Y., Kong, X., Kang, H., Ren, Y., and Wang, W. (2017). Ectopic expression of wheat expansin gene *TaEXPA2* improved the salt tolerance of transgenic tobacco by regulating Na ^+^ /K ^+^ and antioxidant competence. *Physiol. Plant.* 159, 161–177. doi:10.1111/ppl.12492.

Chinpongpanich, A., Limruengroj, K., Phean-O-Pas, S., Limpaseni, T., and Buaboocha, T. (2012). Expression analysis of calmodulin and calmodulin-like genes from rice, Oryza sativa L. *BMC Res. Notes* 5, 625. doi:10.1186/1756-0500-5-625.

Choe, Y.-H., Kim, Y.-S., Kim, I.-S., Bae, M.-J., Lee, E.-J., Kim, Y.-H., Park, H.-M., and Yoon, H.-S. (2013). Homologous expression of γ-glutamylcysteine synthetase increases grain yield and tolerance of transgenic rice plants to environmental stresses. *J. Plant Physiol.* 170, 610–8. doi:10.1016/j.jplph.2012.12.002.

Christov, N. K., Christova, P. K., Kato, H., Liu, Y., Sasaki, K., and Imai, R. (2014). TaSK5, an abiotic stress-inducible GSK3/shaggy-like kinase from wheat, confers salt and drought tolerance in transgenic Arabidopsis. *Plant Physiol. Biochem.* 84, 251–260. doi:10.1016/j.plaphy.2014.10.002.

Dai, X., Xu, Y., Ma, Q., Xu, W., Wang, T., Xue, Y., and Chong, K. (2007). Overexpression of an R1R2R3 MYB gene, OsMYB3R-2, increases tolerance to freezing, drought, and salt stress in transgenic Arabidopsis. *Plant Physiol.* 143, 1739–51. doi:10.1104/pp.106.094532.

Deng, X., Hu, W., Wei, S., Zhou, S., Zhang, F., Han, J., Chen, L., Li, Y., Feng, J., Fang, B., et al. (2013a). TaCIPK29, a CBL-interacting protein kinase gene from wheat, confers salt stress tolerance in transgenic tobacco. *PLoS One* 8, e69881. doi:10.1371/journal.pone.0069881\rPONE-D-13-13631 [pii].

Deng, X., Zhou, S., Hu, W., Feng, J., Zhang, F., Chen, L., Huang, C., Luo, Q., He, Y., Yang, G., et al. (2013b). Ectopic expression of wheat *TaCIPK14* , encoding a calcineurin B-like protein-interacting protein kinase, confers salinity and cold tolerance in tobacco. *Physiol. Plant.* 149, n/a-n/a. doi:10.1111/ppl.12046.

Djemal, R., and Khoudi, H. (2015). Isolation and molecular characterization of a novel WIN1/SHN1 ethylene-responsive transcription factor TdSHN1 from durum wheat (Triticum turgidum. L. subsp. durum). *Protoplasma* 252, 1461–1473. doi:10.1007/s00709-015-0775-8.

Dong, W., Ai, X., Xu, F., Quan, T., Liu, S., and Xia, G. (2012). Isolation and characterization of a bread wheat salinity responsive ERF transcription factor. *Gene* 511, 38–45. doi:10.1016/j.gene.2012.09.039.

Dong, W., Wang, M., Xu, F., Quan, T., Peng, K., Xiao, L., and Xia, G. (2013). Wheat oxophytodienoate reductase gene TaOPR1 confers salinity tolerance via enhancement of abscisic acid signaling and reactive oxygen species scavenging. *Plant Physiol.* 161, 1217–28. doi:10.1104/pp.112.211854.

Du, H. Y., Shen, Y. Z., and Huang, Z. J. (2013a). Function of the wheat TaSIP gene in enhancing drought and salt tolerance in transgenic Arabidopsis and rice. *Plant Mol. Biol.* 81, 417–429. doi:10.1007/s11103-013-0011-x.

Du, X., Zhao, X., Li, X., Guo, C., Lu, W., Gu, J., and Xiao, K. (2013b). Overexpression of TaSRK2C1, a Wheat SNF1-Related Protein Kinase 2 Gene, Increases Tolerance to Dehydration, Salt, and Low Temperature in Transgenic Tobacco. *Plant Mol. Biol. Report.* 31, 810–821. doi:10.1007/s11105-012-0548-x.

Duan, J., Zhang, M., Zhang, H., Xiong, H., Liu, P., Ali, J., Li, J., and Li, Z. (2012). OsMIOX, a myo-inositol oxygenase gene, improves drought tolerance through scavenging of reactive oxygen species in rice (Oryza sativa L.). *Plant Sci.* 196, 143–151. doi:10.1016/j.plantsci.2012.08.003.

Fu, J., Zhang, D.-F., Liu, Y.-H., Ying, S., Shi, Y.-S., Song, Y.-C., Li, Y., and Wang, T.-Y. (2012). Isolation and characterization of maize PMP3 genes involved in salt stress tolerance. *PLoS One* 7, e31101. doi:10.1371/journal.pone.0031101.

Fuchs, I., Stölzle, S., Ivashikina, N., and Hedrich, R. (2005). Rice K+ uptake channel OsAKT1 is sensitive to salt stress. *Planta* 221, 212–21. doi:10.1007/s00425-004-1437-9.

Gao, P., Bai, X., Yang, L., Lv, D., Li, Y., Cai, H., Ji, W., Guo, D., and Zhu, Y. (2010a). Over-expression of osa-MIR396c decreases salt and alkali stress tolerance. *Planta* 231, 991–1001. doi:10.1007/s00425-010-1104-2.

Gao, P., Bai, X., Yang, L., Lv, D., Pan, X., Li, Y., Cai, H., Ji, W., Chen, Q., and Zhu, Y. (2011a). Osa-MIR393: A salinity- and alkaline stress-related microRNA gene. *Mol. Biol. Rep.* 38, 237–242. doi:10.1007/s11033-010-0100-8.

Gao, T., Wu, Y., Zhang, Y., Liu, L., Ning, Y., Wang, D., Tong, H., Chen, S., Chu, C., and Xie, Q. (2011b). OsSDIR1 overexpression greatly improves drought tolerance in transgenic rice. *Plant Mol. Biol.* 76, 145–56. doi:10.1007/s11103-011-9775-z.

Gao, Z., He, X., Zhao, B., Zhou, C., Liang, Y., Ge, R., Shen, Y., and Huang, Z. (2010b). Overexpressing a putative aquaporin gene from wheat, TaNIP, enhances salt tolerance in transgenic arabidopsis. *Plant Cell Physiol.* 51, 767–775. doi:10.1093/pcp/pcq036.

Gouiaa, S., Khoudi, H., Leidi, E. O., Pardo, J. M., and Masmoudi, K. (2012). Expression of wheat Na+/H+ antiporter TNHXS1 and H+- pyrophosphatase TVP1 genes in tobacco from a bicistronic transcriptional unit improves salt tolerance. *Plant Mol. Biol.* 79, 137–155. doi:10.1007/s11103-012-9901-6.

Gu, L., Liu, Y., Zong, X., Liu, L., Li, D. P., and Li, D. Q. (2010). Overexpression of maize mitogen-activated protein kinase gene, ZmSIMK1 in Arabidopsis increases tolerance to salt stress. *Mol. Biol. Rep.* 37, 4067–4073. doi:10.1007/s11033-010-0066-6.

Guan, Q., Xia, D., and Liu, S. (2013). OsAPX4 gene response to several environmental stresses in rice ( Oryza sativa L.). *African J. Biotechnol.* 9. doi:10.4314/ajb.v9i36.

Guo, X., Wu, Y., Wang, Y., Chen, Y., and Chu, C. (2009). OsMSRA4.1 and OsMSRB1.1, two rice plastidial methionine sulfoxide reductases, are involved in abiotic stress responses. *Planta* 230, 227–38. doi:10.1007/s00425-009-0934-2.

Guo, Y., Song, Y., Zheng, H., Zhang, Y., Guo, J., and Sui, N. (2018). NADP-Malate Dehydrogenase of Sweet Sorghum Improves Salt Tolerance of Arabidopsis thaliana. *J. Agric. Food Chem.* 66, 5992–6002. doi:10.1021/acs.jafc.8b02159.

Gupta, O. P., Meena, N. L., Sharma, I., and Sharma, P. (2014). Differential regulation of microRNAs in response to osmotic, salt and cold stresses in wheat. *Mol. Biol. Rep.* 41, 4623–4629. doi:10.1007/s11033-014-3333-0.

Han, J., Zhang, W., Sun, L., Su, Q., Li, Z., Fan, X., Zhang, N., Pan, R., Cui, F., Ji, J., et al. (2016). A Novel Wheat Nicotianamine Synthase Gene, TaNAS-D, Confers High Salt Tolerance in Transgenic Arabidopsis. *Plant Mol. Biol. Report.*, 1–13. doi:10.1007/s11105-016-1018-7.

Han, Y. yang, Li, A. xiu, Li, F., Zhao, M. rong, and Wang, W. (2012). Characterization of a wheat (Triticum aestivum L.) expansin gene, TaEXPB23, involved in the abiotic stress response and phytohormone regulation. *Plant Physiol. Biochem.* 54, 49–58. doi:10.1016/j.plaphy.2012.02.007.

Hasthanasombut, S., Supaibulwatana, K., Mii, M., and Nakamura, I. (2010). Genetic manipulation of Japonica rice using the OsBADH1 gene from Indica rice to improve salinity tolerance. *Plant Cell, Tissue Organ Cult.* 104, 79–89. doi:10.1007/s11240-010-9807-4.

Hauser, F., and Horie, T. (2010). A conserved primary salt tolerance mechanism mediated by HKT transporters: a mechanism for sodium exclusion and maintenance of high K(+)/Na(+) ratio in leaves during salinity stress. *Plant. Cell Environ.* 33, 552–65. doi:10.1111/j.1365-3040.2009.02056.x.

He, X., Hou, X., Shen, Y., and Huang, Z. (2011). TaSRG, a wheat transcription factor, significantly affects salt tolerance in transgenic rice and Arabidopsis. *FEBS Lett.* 585, 1231–1237. doi:10.1016/j.febslet.2011.03.055.

He, Y., Li, W., Lv, J., Jia, Y., Wang, M., and Xia, G. (2012). Ectopic expression of a wheat MYB transcription factor gene, TaMYB73, improves salinity stress tolerance in Arabidopsis thaliana. *J. Exp. Bot.* 63, 1511–1522. doi:10.1093/jxb/err389.

Hou, X., Xie, K., Yao, J., Qi, Z., and Xiong, L. (2009). A homolog of human ski-interacting protein in rice positively regulates cell viability and stress tolerance. *Proc. Natl. Acad. Sci. U. S. A.* 106, 6410–5. doi:10.1073/pnas.0901940106.

Hu, H., You, J., Fang, Y., Zhu, X., Qi, Z., and Xiong, L. (2008). Characterization of transcription factor gene SNAC2 conferring cold and salt tolerance in rice. *Plant Mol. Biol.* 67, 169–81. doi:10.1007/s11103-008-9309-5.

Hu, W., Yan, Y., Hou, X., He, Y., Wei, Y., Yang, G., He, G., and Peng, M. (2015). TaPP2C1, a Group F2 Protein Phosphatase 2C Gene, Confers Resistance to Salt Stress in Transgenic Tobacco. *PLoS One* 10, e0129589. doi:10.1371/journal.pone.0129589.

Hu, W., Yuan, Q., Wang, Y., Cai, R., Deng, X., Wang, J., Zhou, S., Chen, M., Chen, L., Huang, C., et al. (2012). Overexpression of a wheat aquaporin gene, TaAQP8, enhances salt stress tolerance in transgenic tobacco. *Plant Cell Physiol.* 53, 2127–2141. doi:10.1093/pcp/pcs154.

Huang, J., Sun, S., Xu, D., Lan, H., Sun, H., Wang, Z., Bao, Y., Wang, J., Tang, H., and Zhang, H. (2012a). A TFIIIA-type zinc finger protein confers multiple abiotic stress tolerances in transgenic rice (Oryza sativa L.). *Plant Mol. Biol.* 80, 337–50. doi:10.1007/s11103-012-9955-5.

Huang, J., Wang, M.-M., Bao, Y.-M., Sun, S.-J., Pan, L.-J., and Zhang, H.-S. (2008a). SRWD: a novel WD40 protein subfamily regulated by salt stress in rice (OryzasativaL.). *Gene* 424, 71–9. doi:10.1016/j.gene.2008.07.027.

Huang, J., Wang, M.-M., Jiang, Y., Wang, Q.-H., Huang, X., and Zhang, H.-S. (2008b). Stress repressive expression of rice SRZ1 and characterization of plant SRZ gene family. *Plant Sci.* 174, 227–235. doi:10.1016/j.plantsci.2007.11.010.

Huang, Q., Wang, Y., Li, B., Chang, J., Chen, M., Li, K., Yang, G., He, G., and G(8). (2015). TaNAC29, a NAC transcription factor from wheat, enhances salt and drought tolerance in transgenic Arabidopsis. *BMC Plant Biol.* 15, 1–15. doi:10.1186/s12870-015-0644-9.

Huang, X., Wang, G., Shen, Y., and Huang, Z. (2012b). The wheat gene TaST can increase the salt tolerance of transgenic Arabidopsis. *Plant Cell Rep.* 31, 339–347. doi:10.1007/s00299-011-1169-9.

Huang, X., Zhang, Y., Jiao, B., Chen, G., Huang, S., Guo, F., Shen, Y., Huang, Z., and Zhao, B. (2012c). Overexpression of the wheat salt tolerance-related gene TaSC enhances salt tolerance in Arabidopsis. *J. Exp. Bot.* 63, 5463–5473. doi:10.1093/jxb/ers198.

Huang, X.-Y., Chao, D.-Y., Gao, J.-P., Zhu, M.-Z., Shi, M., and Lin, H.-X. (2009). A previously unknown zinc finger protein, DST, regulates drought and salt tolerance in rice via stomatal aperture control. *Genes Dev.* 23, 1805–17. doi:10.1101/gad.1812409.

Huang, Y.-W., Tsay, W.-S., Chen, C.-C., Lin, C.-W., and Huang, H.-J. (2008c). Increased expression of the rice C-type cyclin-dependent protein kinase gene, Orysa;CDKC;1, in response to salt stress. *Plant Physiol. Biochem. PPB / Société Fr. Physiol. végétale* 46, 71–81. doi:10.1016/j.plaphy.2007.10.013.

Huda, K. M. K., Banu, M. S. A., Garg, B., Tula, S., Tuteja, R., and Tuteja, N. (2013). OsACA6, a P-type IIB Ca^2+^ ATPase promotes salinity and drought stress tolerance in tobacco by ROS scavenging and enhancing the expression of stress-responsive genes. *Plant J.* 76, 997–1015. doi:10.1111/tpj.12352.

Jain, M., Tyagi, A. K., and Khurana, J. P. (2006). Overexpression of putative topoisomerase 6 genes from rice confers stress tolerance in transgenic Arabidopsis plants. *FEBS J.* 273, 5245–60. doi:10.1111/j.1742-4658.2006.05518.x.

James, R. A., Blake, C., Byrt, C. S., and Munns, R. (2011). Major genes for Na+ exclusion, Nax1 and Nax2 (wheat HKT1;4 and HKT1;5), decrease Na+ accumulation in bread wheat leaves under saline and waterlogged conditions. *J. Exp. Bot.* 62, 2939–2947. doi:10.1093/jxb/err003.

Jamil, M., Iqbal, W., Bangash, A., Rehman, S. U., Muhammad Imran, Q., and Rha, E. S. (2010). Constitutive expression of OSC3H33, OsC3H50 and OSC3H37 genes in rice under salt stress. *Pakistan J. Bot.* 42, 4003–4009.

Jan, A., Maruyama, K., Todaka, D., Kidokoro, S., Abo, M., Yoshimura, E., Shinozaki, K., Nakashima, K., and Yamaguchi-Shinozaki, K. (2013). OsTZF1, a CCCH-tandem zinc finger protein, confers delayed senescence and stress tolerance in rice by regulating stress-related genes. *Plant Physiol.* 161, 1202–16. doi:10.1104/pp.112.205385.

Jeong, M.-J., Lee, S.-K., Kim, B.-G., Kwon, T.-R., Cho, W.-S., Park, Y.-T., Lee, J.-O., Kwon, H.-B., Byun, M.-O., and Park, S.-C. (2006). A rice (Oryza sativa L.) MAP kinase gene, OsMAPK44, is involved in response to abiotic stresses. *Plant Cell. Tissue Organ Cult.* 85, 151–160. doi:10.1007/s11240-005-9064-0.

Jin, S., Sun, D., Wang, J., Li, Y., Wang, X., and Liu, S. (2014). Expression of the rgMT gene, encoding for a rice metallothionein-like protein in Saccharomyces cerevisiae and Arabidopsis thaliana. *J. Genet.* 93, 709–18. Available at: http://www.ncbi.nlm.nih.gov/pubmed/25572229 [Accessed March 15, 2016].

Jin, X., Sun, T., Wang, X., Su, P., Ma, J., He, G., and Yang, G. (2016). Wheat CBL-interacting protein kinase 25 negatively regulates salt tolerance in transgenic wheat. *Sci. Rep.* 6, 28884. doi:10.1038/srep28884.

Jin, X., Xue, Y., Wang, R., Xu, R., Bian, L., Zhu, B., Han, H., Peng, R., and Yao, Q. (2013). Transcription factor OsAP21 gene increases salt/drought tolerance in transgenic Arabidopsis thaliana. *Mol. Biol. Rep.* 40, 1743–52. doi:10.1007/s11033-012-2228-1.

Jung, H. J., and Kang, H. (2007). Expression and functional analyses of microRNA417 in Arabidopsis thaliana under stress conditions. *Plant Physiol. Biochem.* 45, 805–811. doi:10.1016/j.plaphy.2007.07.015.

Kanneganti, V., and Gupta, A. K. (2008). Overexpression of OsiSAP8, a member of stress associated protein (SAP) gene family of rice confers tolerance to salt, drought and cold stress in transgenic tobacco and rice. *Plant Mol. Biol.* 66, 445–62. doi:10.1007/s11103-007-9284-2.

Kim, J. Y., Kwak, K. J., Jung, H. J., Lee, H. J., and Kang, H. (2010a). MicroRNA402 affects seed germination of arabidopsis thaliana under stress conditions via targeting DEMETER-LIKE Protein3 mRNA. *Plant Cell Physiol.* 51, 1079–1083. doi:10.1093/pcp/pcq072.

Kim, J. Y., Lee, H. J., Jung, H. J., Maruyama, K., Suzuki, N., and Kang, H. (2010b). Overexpression of microRNA395c or 395e affects differently the seed germination of arabidopsis thaliana under stress conditions. *Planta* 232, 1447–1454. doi:10.1007/s00425-010-1267-x.

Kim, S.-K., You, Y. N., Park, J. C., Joung, Y., Kim, B.-G., Ahn, J. C., and Cho, H. S. (2012). The rice thylakoid lumenal cyclophilin OsCYP20-2 confers enhanced environmental stress tolerance in tobacco and Arabidopsis. *Plant Cell Rep.* 31, 417–26. doi:10.1007/s00299-011-1176-x.

Kruszka, K., Pieczynski, M., Windels, D., Bielewicz, D., Jarmolowski, A., Szweykowska-Kulinska, Z., and Vazquez, F. (2012). Role of microRNAs and other sRNAs of plants in their changing environments. *J. Plant Physiol.* 169, 1664–1672. doi:10.1016/j.jplph.2012.03.009.

Kumar, G., bullet, Kushwaha, H. R., @bullet, R. S., Purty, Kumari, bullet S., Sneh, bullet, Singla-Pareek, L., and Pareek, bullet A. (2009). Cloning, Structural and Expression Analysis of OsSOS2 in Contrasting Cultivars of Rice under Salinity Stress. in *6th International Rice Genetics Symposium* Available at: https://www.researchgate.net/publication/232769335_Cloning_Structural_and_Expression_Analysis_of_OsSOS2_in_Contrasting_Cultivars_of_Rice_under_Salinity_Stress [Accessed March 15, 2016].

Kumar, G., Kushwaha, H. R., Panjabi-Sabharwal, V., Kumari, S., Joshi, R., Karan, R., Mittal, S., Pareek, S. L. S., and Pareek, A. (2012). Clustered metallothionein genes are co-regulated in rice and ectopic expression of OsMT1e-P confers multiple abiotic stress tolerance in tobacco via ROS scavenging. *BMC Plant Biol.* 12, 107. doi:10.1186/1471-2229-12-107.

Kumar, K., Rao, K. P., Biswas, D. K., and Sinha, A. K. (2011). Rice WNK1 is regulated by abiotic stress and involved in internal circadian rhythm. *Plant Signal. Behav.* 6, 316–20. Available at: http://www.pubmedcentral.nih.gov/articlerender.fcgi?artid=3142407&tool=pmcentrez&rendertype=abstract [Accessed March 15, 2016].

Kusuda, H., Koga, W., Kusano, M., Oikawa, A., Saito, K., Hirai, M. Y., and Yoshida, K. T. (2015). Ectopic expression of myo-inositol 3-phosphate synthase induces a wide range of metabolic changes and confers salt tolerance in rice. *Plant Sci.* 232, 49–56. doi:10.1016/j.plantsci.2014.12.009.

Lakra, N., Nutan, K. K., Das, P., Anwar, K., Singla-Pareek, S. L., and Pareek, A. (2015). A nuclear-localized histone-gene binding protein from rice (OsHBP1b) functions in salinity and drought stress tolerance by maintaining chlorophyll content and improving the antioxidant machinery. *J. Plant Physiol.* 176, 36–46. doi:10.1016/j.jplph.2014.11.005.

Li, C., Lv, J., Zhao, X., Ai, X., Zhu, X., Wang, M., Zhao, S., and Xia, G. (2010a). TaCHP: a wheat zinc finger protein gene down-regulated by abscisic acid and salinity stress plays a positive role in stress tolerance. *Plant Physiol.* 154, 211–21. doi:10.1104/pp.110.161182.

Li, F., Guo, S., Zhao, Y., Chen, D., Chong, K., and Xu, Y. (2010b). Overexpression of a homopeptide repeat-containing bHLH protein gene (OrbHLH001) from Dongxiang Wild Rice confers freezing and salt tolerance in transgenic Arabidopsis. *Plant Cell Rep.* 29, 977–86. doi:10.1007/s00299-010-0883-z.

Li, H.-W., Zang, B.-S., Deng, X.-W., and Wang, X.-P. (2011). Overexpression of the trehalose-6-phosphate synthase gene OsTPS1 enhances abiotic stress tolerance in rice. *Planta* 234, 1007–1018. doi:10.1007/s00425-011-1458-0.

Li, S., Xu, C., Yang, Y., and Xia, G. (2010c). Functional analysis of TaDi19A, a salt-responsive gene in wheat. *Plant, Cell Environ.* 33, 117–129. doi:10.1111/j.1365-3040.2009.02063.x.

Li, Y., Liang, W., Han, J., and Huang, Z. (2016). A novel TaSST gene from wheat contributes to enhanced resistance to salt stress in Arabidopsis thaliana and Oryza sativa. *Acta Physiol. Plant.* 38, 113. doi:10.1007/s11738-016-2130-x.

Liang, W., Cui, W., Ma, X., Wang, G., and Huang, Z. (2014). Function of wheat Ta-UnP gene in enhancing salt tolerance in transgenic Arabidopsis and rice. *Biochem. Biophys. Res. Commun.* 450, 794–801. doi:10.1016/j.bbrc.2014.06.055.

Lin, F., Xu, J., Shi, J., Li, H., and Li, B. (2010). Molecular cloning and characterization of a novel glyoxalase I gene TaGly I in wheat (Triticum aestivum L.). *Mol. Biol. Rep.* 37, 729–735. doi:10.1007/s11033-009-9578-3.

Liu, A.-L., Zou, J., Liu, C.-F., Zhou, X.-Y., Zhang, X.-W., Luo, G.-Y., and Chen, X.-B. (2013a). Over-expression of OsHsfA7 enhanced salt and drought tolerance in transgenic rice. *BMB Rep.* 46, 31–6. Available at: http://www.pubmedcentral.nih.gov/articlerender.fcgi?artid=4133825&tool=pmcentrez&rendertype=abstract [Accessed February 22, 2016].

Liu, C., Mao, B., Ou, S., Wang, W., Liu, L., Wu, Y., Chu, C., and Wang, X. (2014a). OsbZIP71, a bZIP transcription factor, confers salinity and drought tolerance in rice. *Plant Mol. Biol.* 84, 19–36. doi:10.1007/s11103-013-0115-3.

Liu, D., Chen, X., Liu, J., Ye, J., and Guo, Z. (2012). The rice ERF transcription factor OsERF922 negatively regulates resistance to Magnaporthe oryzae and salt tolerance. *J. Exp. Bot.* 63, 3899–3912. doi:10.1093/jxb/ers079.

Liu, H., Zhou, X., Dong, N., Liu, X., Zhang, H., and Zhang, Z. (2011). Expression of a wheat MYB gene in transgenic tobacco enhances resistance to Ralstonia solanacearum, and to drought and salt stresses. *Funct. Integr. Genomics* 11, 431–443. doi:10.1007/s10142-011-0228-1.

Liu, H.-H., Tian, X., Li, Y.-J., Wu, C.-A., and Zheng, C.-C. (2008). Microarray-based analysis of stress-regulated microRNAs in Arabidopsis thaliana. *RNA* 14, 836–843. doi:10.1261/rna.895308.

Liu, H.-L., Dai, X.-Y., Xu, Y.-Y., and Chong, K. (2007a). Over-expression of OsUGE-1 altered raffinose level and tolerance to abiotic stress but not morphology in Arabidopsis. *J. Plant Physiol.* 164, 1384–90. doi:10.1016/j.jplph.2007.03.005.

Liu, K., Wang, L., Xu, Y., Chen, N., Ma, Q., Li, F., and Chong, K. (2007b). Overexpression of OsCOIN, a putative cold inducible zinc finger protein, increased tolerance to chilling, salt and drought, and enhanced proline level in rice. *Planta* 226, 1007–16. doi:10.1007/s00425-007-0548-5.

Liu, P., Xu, Z. S., Pan-Pan, L., Hu, D., Chen, M., Li, L. C., and Ma, Y. Z. (2013b). A wheat PI4K gene whose product possesses threonine autophophorylation activity confers tolerance to drought and salt in Arabidopsis. *J. Exp. Bot.* 64, 2915–2927. doi:10.1093/jxb/ert133.

Liu, S., Liu, S., Wang, M., Wei, T., Meng, C., Wang, M., and Xia, G. (2014b). A wheat SIMILAR TO RCD-ONE gene enhances seedling growth and abiotic stress resistance by modulating redox homeostasis and maintaining genomic integrity. *Plant Cell* 26, 164–80. doi:10.1105/tpc.113.118687.

Lu, W., Guo, C., Li, X., Duan, W., Ma, C., Zhao, M., Gu, J., Du, X., Liu, Z., and Xiao, K. (2014). Overexpression of TaNHX3, a vacuolar Na+/H+ antiporter gene inwheat, enhances salt stress tolerance in tobacco by improving related physiological processes. *Plant Physiol. Biochem.* 76, 17–28. doi:10.1016/j.plaphy.2013.12.013.

Lu, W., Li, J., Liu, F., Gu, J., Guo, C., Xu, L., Zhang, H., and Xiao, K. (2011). Expression pattern of wheat miRNAs under salinity stress and prediction of salt-inducible miRNAs targets. *Front. Agric. China* 5, 413–422. doi:10.1007/s11703-011-1133-z.

Lu, Z., Liu, D., and Liu, S. (2007). Two rice cytosolic ascorbate peroxidases differentially improve salt tolerance in transgenic Arabidopsis. *Plant Cell Rep.* 26, 1909–17. doi:10.1007/s00299-007-0395-7.

Luo, H., Song, F., Goodman, R. M., and Zheng, Z. (2005). Up-regulation of OsBIHD1, a rice gene encoding BELL homeodomain transcriptional factor, in disease resistance responses. *Plant Biol. (Stuttg).* 7, 459–68. doi:10.1055/s-2005-865851.

Luo, M., Gu, S.-H., Zhao, S.-H., Zhang, F., and Wu, N.-H. (2006). Rice GTPase OsRacB: potential accessory factor in plant salt-stress signaling. *Acta Biochim. Biophys. Sin. (Shanghai).* 38, 393–402. Available at: http://www.ncbi.nlm.nih.gov/pubmed/16761097 [Accessed March 15, 2016].

Ma, X., Cui, W., Liang, W., and Huang, Z. (2015). Wheat TaSP gene improves salt tolerance in transgenic Arabidopsis thaliana. *Plant Physiol. Biochem.* 97, 187–195. doi:10.1016/j.plaphy.2015.10.010.

Macovei, A., and Tuteja, N. (2012). microRNAs targeting DEAD-box helicases are involved in salinity stress response in rice (Oryza sativa L.). *BMC Plant Biol.* 12, 183. doi:10.1186/1471-2229-12-183.

Macovei, A., Vaid, N., Tula, S., and Tuteja, N. (2012). A new DEAD-box helicase ATP-binding protein (OsABP) from rice is responsive to abiotic stress. *Plant Signal. Behav.* 7, 1138–43. doi:10.4161/psb.21343.

Makhloufi, E., Yousfi, F. E., Marande, W., Mila, I., Hanana, M., Berg s, H., Mzid, R., and Bouzayen, M. (2014). Isolation and molecular characterization of ERF1, an ethylene response factor gene from durum wheat (Triticum turgidum L. subsp. durum), potentially involved in salt-stress responses. *J. Exp. Bot.* 65, 6359–6371. doi:10.1093/jxb/eru352.

Mao, H., Yu, L., Han, R., Li, Z., and Liu, H. (2016). ZmNAC55, a maize stress-responsive NAC transcription factor, confers drought resistance in transgenic Arabidopsis. *Plant Physiol. Biochem.* 105, 55–66. doi:10.1016/j.plaphy.2016.04.018.

Mao, X., Chen, S., Li, A., Zhai, C., and Jing, R. (2014). Novel NAC transcription factor TaNAC67 confers enhanced multi-abiotic stress tolerances in Arabidopsis. *PLoS One* 9. doi:10.1371/journal.pone.0084359.

Mao, X., Zhang, H., Qian, X., Li, A., Zhao, G., and Jing, R. (2012). TaNAC2, a NAC-type wheat transcription factor conferring enhanced multiple abiotic stress tolerances in Arabidopsis. *J. Exp. Bot.* 63, 2933–2946. doi:10.1093/jxb/err462.

Mao, X., Zhang, H., Tian, S., Chang, X., and Jing, R. (2010). TaSnRK2.4, an SNF1-type serine/threonine protein kinase of wheat (Triticum aestivum L.), confers enhanced multistress tolerance in Arabidopsis. *J. Exp. Bot.* 61, 683–96. doi:10.1093/jxb/erp331.

Matsuda, S., Nagasawa, H., Yamashiro, N., Yasuno, N., Watanabe, T., Kitazawa, H., Takano, S., Tokuji, Y., Tani, M., Takamure, I., et al. (2014). Rice RCN1/OsABCG5 mutation alters accumulation of essential and nonessential minerals and causes a high Na/K ratio, resulting in a salt-sensitive phenotype. *Plant Sci.* 224, 103–11. doi:10.1016/j.plantsci.2014.04.011.

Mian, A., Oomen, R. J. F. J., Isayenkov, S., Sentenac, H., Maathuis, F. J. M., and Véry, A.-A. (2011). Over-expression of an Na+- and K+-permeable HKT transporter in barley improves salt tolerance. *Plant J.* 68, 468–479. doi:10.1111/j.1365-313X.2011.04701.x.

Moons, A., Gielen, J., Vandekerckhove, J., Van der Straeten, D., Gheysen, G., and Van Montagu, M. (1997). An abscisic-acid- and salt-stress-responsive rice cDNA from a novel plant gene family. *Planta* 202, 443–54. Available at: http://www.ncbi.nlm.nih.gov/pubmed/9265787 [Accessed March 15, 2016].

Nahm, M. Y., Kim, S. W., Yun, D., Lee, S. Y., Cho, M. J., and Bahk, J. D. (2003). Molecular and biochemical analyses of OsRab7, a rice Rab7 homolog. *Plant Cell Physiol.* 44, 1341–9. Available at: http://www.ncbi.nlm.nih.gov/pubmed/14701929 [Accessed March 15, 2016].

Nakamura, A., Fukuda, A., Sakai, S., and Tanaka, Y. (2006). Molecular cloning, functional expression and subcellular localization of two putative vacuolar voltage-gated chloride channels in rice (Oryza sativa L.). *Plant Cell Physiol.* 47, 32–42. doi:10.1093/pcp/pci220.

Nakashima, K., Tran, L.-S. P., Van Nguyen, D., Fujita, M., Maruyama, K., Todaka, D., Ito, Y., Hayashi, N., Shinozaki, K., and Yamaguchi-Shinozaki, K. (2007). Functional analysis of a NAC-type transcription factor OsNAC6 involved in abiotic and biotic stress-responsive gene expression in rice. *Plant J.* 51, 617–30. doi:10.1111/j.1365-313X.2007.03168.x.

Nayidu, N. K., Wang, L., Xie, W., Zhang, C., Fan, C., Lian, X., Zhang, Q., and Xiong, L. (2008). Comprehensive sequence and expression profile analysis of PEX11 gene family in rice. *Gene* 412, 59–70. doi:10.1016/j.gene.2008.01.006.

Ning, J., Li, X., Hicks, L. M., and Xiong, L. (2010). A Raf-like MAPKKK gene DSM1 mediates drought resistance through reactive oxygen species scavenging in rice. *Plant Physiol.* 152, 876–90. doi:10.1104/pp.109.149856.

Niu, C. F., Wei, W., Zhou, Q. Y., Tian, A. G., Hao, Y. J., Zhang, W. K., Ma, B., Lin, Q., Zhang, Z. B., Zhang, J. S., et al. (2012). Wheat WRKY genes TaWRKY2 and TaWRKY19 regulate abiotic stress tolerance in transgenic Arabidopsis plants. *Plant, Cell Environ.* 35, 1156–1170. doi:10.1111/j.1365-3040.2012.02480.x.

OHTSU, K., ITO, Y., SAIKA, H., NAKAZONO, M., TSUTSUMI, N., and HIRAI, A. (2002). ABA-Independent Expression of Rice Alternative Oxidase Genes under Environmental Stresses. *Plant Biotechnol.* 19, 187–190. doi:10.5511/plantbiotechnology.19.187.

Ouyang, S.-Q., Liu, Y.-F., Liu, P., Lei, G., He, S.-J., Ma, B., Zhang, W.-K., Zhang, J.-S., and Chen, S.-Y. (2010). Receptor-like kinase OsSIK1 improves drought and salt stress tolerance in rice (Oryza sativa) plants. *Plant J.* 62, 316–29. doi:10.1111/j.1365-313X.2010.04146.x.

Park, J.-J., Yi, J., Yoon, J., Cho, L.-H., Ping, J., Jeong, H. J., Cho, S. K., Kim, W. T., and An, G. (2011). OsPUB15, an E3 ubiquitin ligase, functions to reduce cellular oxidative stress during seedling establishment. *Plant J.* 65, 194–205. doi:10.1111/j.1365-313X.2010.04416.x.

Parveda, M., Kiran, B., Punita, D. L., and Kavi Kishor, P. B. (2017). Overexpression of SbAP37 in rice alleviates concurrent imposition of combination stresses and modulates different sets of leaf protein profiles. *Plant Cell Rep.* 36, 773–786. doi:10.1007/s00299-017-2134-z.

Qi, Y., Kawano, N., Yamauchi, Y., Ling, J., Li, D., and Tanaka, K. (2005). Identification and cloning of a submergence-induced gene OsGGT (glycogenin glucosyltransferase) from rice (Oryza sativa L.) by suppression subtractive hybridization. *Planta* 221, 437–45. doi:10.1007/s00425-004-1453-9.

QIAO, W., XIAO, S., YU, L., and FAN, L. (2009). Expression of a rice gene OsNOA1 re-establishes nitric oxide synthesis and stress-related gene expression for salt tolerance in Arabidopsis nitric oxide-associated 1 mutant Atnoa1. *Environ. Exp. Bot.* 65, 90–98. doi:10.1016/j.envexpbot.2008.06.002.

Qin, Y., Tian, Y., Han, L., and Yang, X. (2013). Constitutive expression of a salinity-induced wheat WRKY transcription factor enhances salinity and ionic stress tolerance in transgenic Arabidopsis thaliana. *Biochem. Biophys. Res. Commun.* 441, 476–81. Available at: http://www.ncbi.nlm.nih.gov/pubmed/24383079 [Accessed February 7, 2017].

Qin, Y., Tian, Y., and Liu, X. (2015). A wheat salinity-induced WRKY transcription factor TaWRKY93 confers multiple abiotic stress tolerance in Arabidopsis thaliana. *Biochem. Biophys. Res. Commun.* 464, 428–433. doi:10.1016/j.bbrc.2015.06.128.

Qin, Y., Wang, M., Tian, Y., He, W., Han, L., and Xia, G. (2012). Over-expression of TaMYB33 encoding a novel wheat MYB transcription factor increases salt and drought tolerance in Arabidopsis. *Mol. Biol. Rep.* 39, 7183–7192. doi:10.1007/s11033-012-1550-y.

Qin, Y., Ye, H., Tang, N., and Xiong, L. (2009). Systematic identification of X1-homologous genes reveals a family involved in stress responses in rice. *Plant Mol. Biol.* 71, 483–96. doi:10.1007/s11103-009-9535-5.

Qiu, D., Xiao, J., Ding, X., Xiong, M., Cai, M., Cao, Y., Li, X., Xu, C., and Wang, S. (2007). OsWRKY13 mediates rice disease resistance by regulating defense-related genes in salicylate- and jasmonate-dependent signaling. *Mol. Plant. Microbe. Interact.* 20, 492–9. doi:10.1094/MPMI-20-5-0492.

Rahaie, M., Xue, G.-P., Naghavi, M. R., Alizadeh, H., and Schenk, P. M. (2010). A MYB gene from wheat (Triticum aestivum L.) is up-regulated during salt and drought stresses and differentially regulated between salt-tolerant and sensitive genotypes. *Plant Cell Rep.* 29, 835–844. doi:10.1007/s00299-010-0868-y.

Ren, Z.-H., Gao, J.-P., Li, L.-G., Cai, X.-L., Huang, W., Chao, D.-Y., Zhu, M.-Z., Wang, Z.-Y., Luan, S., and Lin, H.-X. (2005). A rice quantitative trait locus for salt tolerance encodes a sodium transporter. *Nat. Genet.* 37, 1141–6. doi:10.1038/ng1643.

Rong, W., Qi, L., Wang, A., Ye, X., Du, L., Liang, H., Xin, Z., and Zhang, Z. (2014). The ERF transcription factor TaERF3 promotes tolerance to salt and drought stresses in wheat. *Plant Biotechnol. J.* 12, 468–479. doi:10.1111/pbi.12153.

Roy, M., and Wu, R. (2002). Overexpression of S-adenosylmethionine decarboxylase gene in rice increases polyamine level and enhances sodium chloride-stress tolerance. *Plant Sci.* 163, 987–992. doi:10.1016/S0168-9452(02)00272-8.

Rus, A. M., Bressan, R. A., and Hasegawa, P. M. (2005). Unraveling salt tolerance in crops. *Nat. Genet.* 37, 1029–30. doi:10.1038/ng1005-1029.

Saad, A. S. I., Li, X., Li, H.-P., Huang, T., Gao, C.-S., Guo, M.-W., Cheng, W., Zhao, G.-Y., and Liao, Y.-C. (2013). A rice stress-responsive NAC gene enhances tolerance of transgenic wheat to drought and salt stresses. *Plant Sci.* 203–204, 33–40. doi:10.1016/j.plantsci.2012.12.016.

Saeng-ngam, S., Takpirom, W., Buaboocha, T., and Chadchawan, S. (2012). The role of the OsCam1-1 salt stress sensor in ABA accumulation and salt tolerance in rice. *J. Plant Biol.* 55, 198–208. doi:10.1007/s12374-011-0154-8.

Schmidt, R., Mieulet, D., Hubberten, H.-M., Obata, T., Hoefgen, R., Fernie, A. R., Fisahn, J., San Segundo, B., Guiderdoni, E., Schippers, J. H. M., et al. (2013). Salt-responsive ERF1 regulates reactive oxygen species-dependent signaling during the initial response to salt stress in rice. *Plant Cell* 25, 2115–31. doi:10.1105/tpc.113.113068.

Schmidt, R., Schippers, J. H. M., Welker, A., Mieulet, D., Guiderdoni, E., and Mueller-Roeber, B. (2012). Transcription factor OsHsfC1b regulates salt tolerance and development in Oryza sativa ssp. japonica. *AoB Plants* 2012, pls011. doi:10.1093/aobpla/pls011.

Senadheera, P., Singh, R. K., and Maathuis, F. J. M. (2009). Differentially expressed membrane transporters in rice roots may contribute to cultivar dependent salt tolerance. *J. Exp. Bot.* 60, 2553–63. doi:10.1093/jxb/erp099.

Shan, L., Li, C., Chen, F., Zhao, S., and Xia, G. (2008). A Bowman-Birk type protease inhibitor is involved in the tolerance to salt stress in wheat. *Plant, Cell Environ.* 31, 1128–1137. doi:10.1111/j.1365-3040.2008.01825.x.

Singh, A. K., Kumar, R., Pareek, A., Sopory, S. K., and Singla-Pareek, S. L. (2012). Overexpression of rice CBS domain containing protein improves salinity, oxidative, and heavy metal tolerance in transgenic tobacco. *Mol. Biotechnol.* 52, 205–16. doi:10.1007/s12033-011-9487-2.

Song, J. B., Gao, S., Sun, D., Li, H., Shu, X. X., and Yang, Z. M. (2013). miR394 and LCR are involved in Arabidopsis salt and drought stress responses in an abscisic acid-dependent manner. *BMC Plant Biol.* 13, 210. doi:10.1186/1471-2229-13-210.

Sripinyowanich, S., Klomsakul, P., Boonburapong, B., Bangyeekhun, T., Asami, T., Gu, H., Buaboocha, T., and Chadchawan, S. (2013). Exogenous ABA induces salt tolerance in indica rice (Oryza sativa L.): The role of OsP5CS1 and OsP5CR gene expression during salt stress. *Environ. Exp. Bot.* 86, 94–105. doi:10.1016/j.envexpbot.2010.01.009.

Sun, X., Qi, W., Yue, Y., Ling, H., Wang, G., and Song, R. (2016). Maize ZmVPP5 is a truncated Vacuole H+-PPase that confers hypersensitivity to salt stress. *J. Integr. Plant Biol.* 58, 518–528. doi:10.1111/jipb.12462.

Sun, Y., Xu, W., Jia, Y., Wang, M., and Xia, G. (2015). The wheat *TaGBF1* gene is involved in the blue-light response and salt tolerance. *Plant J.* 84, 1219–1230. doi:10.1111/tpj.13082.

Takasaki, H., Maruyama, K., Kidokoro, S., Ito, Y., Fujita, Y., Shinozaki, K., Yamaguchi-Shinozaki, K., and Nakashima, K. (2010). The abiotic stress-responsive NAC-type transcription factor OsNAC5 regulates stress-inducible genes and stress tolerance in rice. *Mol. Genet. Genomics* 284, 173–83. doi:10.1007/s00438-010-0557-0.

Tan, C.-M., Chen, R.-J., Zhang, J.-H., Gao, X.-L., Li, L.-H., Wang, P.-R., Deng, X.-J., and Xu, Z.-J. (2013). OsPOP5, a prolyl oligopeptidase family gene from rice confers abiotic stress tolerance in Escherichia coli. *Int. J. Mol. Sci.* 14, 20204–19. doi:10.3390/ijms141020204.

Tao, Z., Kou, Y., Liu, H., Li, X., Xiao, J., and Wang, S. (2011). OsWRKY45 alleles play different roles in abscisic acid signalling and salt stress tolerance but similar roles in drought and cold tolerance in rice. *J. Exp. Bot.* 62, 4863–4874. doi:10.1093/jxb/err144.

Teixeira, F. K., Menezes-Benavente, L., Galvão, V. C., Margis, R., and Margis-Pinheiro, M. (2006). Rice ascorbate peroxidase gene family encodes functionally diverse isoforms localized in different subcellular compartments. *Planta* 224, 300–14. doi:10.1007/s00425-005-0214-8.

Wan, B., Lin, Y., and Mou, T. (2007). Expression of rice Ca(2+)-dependent protein kinases (CDPKs) genes under different environmental stresses. *FEBS Lett.* 581, 1179–89. doi:10.1016/j.febslet.2007.02.030.

Wang, B., Sun, Y. fei, Song, N., Wei, J. ping, Wang, X. jie, Feng, H., Yin, Z. yuan, and Kang, Z. sheng (2014a). MicroRNAs involving in cold, wounding and salt stresses in Triticum aestivum L. *Plant Physiol. Biochem.* 80, 90–96. doi:10.1016/j.plaphy.2014.03.020.

Wang, C., Deng, P., Chen, L., Wang, X., Ma, H., Hu, W., Yao, N., Feng, Y., Chai, R., Yang, G., et al. (2013a). A Wheat WRKY Transcription Factor TaWRKY10 Confers Tolerance to Multiple Abiotic Stresses in Transgenic Tobacco. *PLoS One* 8. doi:10.1371/journal.pone.0065120.

Wang, C., Lu, G., Hao, Y., Guo, H., Guo, Y., Zhao, J., and Cheng, H. (2017). ABP9, a maize bZIP transcription factor, enhances tolerance to salt and drought in transgenic cotton. *Planta* 246, 453–469. doi:10.1007/s00425-017-2704-x.

Wang, J., Ding, B., Guo, Y., Li, M., Chen, S., Huang, G., and Xie, X. (2014b). Overexpression of a wheat phospholipase D gene, TaPLDα, enhances tolerance to drought and osmotic stress in Arabidopsis thaliana. *Planta* 240, 103–115. doi:10.1007/s00425-014-2066-6.

Wang, M., Liu, C., Li, S., Zhu, D., Zhao, Q., and Yu, J. (2013b). Improved nutritive quality and salt resistance in transgenic maize by simultaneously overexpression of a natural lysine-rich protein gene, SBgLR, and an ERF transcription factor gene, TSRF1. *Int. J. Mol. Sci.* 14, 9459–9474. doi:10.3390/ijms14059459.

Wang, M., Zhao, X., Xiao, Z., Yin, X., Xing, T., and Xia, G. (2016). A wheat superoxide dismutase gene TaSOD2 enhances salt resistance through modulating redox homeostasis by promoting NADPH oxidase activity. *Plant Mol. Biol.* 91, 115–130. doi:10.1007/s11103-016-0446-y.

Wang, Q., Guan, Y., Wu, Y., Chen, H., Chen, F., and Chu, C. (2008). Overexpression of a rice OsDREB1F gene increases salt, drought, and low temperature tolerance in both Arabidopsis and rice. *Plant Mol. Biol.* 67, 589–602. doi:10.1007/s11103-008-9340-6.

Wang, R., Jing, W., Xiao, L., Jin, Y., Shen, L., and Zhang, W. (2015a). The Rice High-Affinity Potassium Transporter1;1 Is Involved in Salt Tolerance and Regulated by an MYB-Type Transcription Factor. *Plant Physiol.* 168, 1076–90. doi:10.1104/pp.15.00298.

Wang, S., Uddin, M. I., Tanaka, K., Yin, L., Shi, Z., Qi, Y., Mano, J., Matsui, K., Shimomura, N., Sakaki, T., et al. (2014c). Maintenance of Chloroplast Structure and Function by Overexpression of the Rice MONOGALACTOSYLDIACYLGLYCEROL SYNTHASE Gene Leads to Enhanced Salt Tolerance in Tobacco. *Plant Physiol.* 165, 1144–1155. doi:10.1104/pp.114.238899.

Wang, T. T., Ren, Z. J., Liu, Z. Q., Feng, X., Guo, R. Q., Li, B. G., Li, L. G., and Jing, H. C. (2014d). SbHKT1;4, a member of the high-affinity potassium transporter gene family from Sorghum bicolor, functions to maintain optimal Na+/K+ balance under Na+ stress. *J. Integr. Plant Biol.* 56, 315–332. doi:10.1111/jipb.12144.

Wang, X., Zeng, J., Li, Y., Rong, X., Sun, J., Sun, T., Li, M., Wang, L., Feng, Y., Chai, R., et al. (2015b). Expression of TaWRKY44, a wheat WRKY gene, in transgenic tobacco confers multiple abiotic stress tolerances. *Front. Plant Sci.* 6, 615. doi:10.3389/fpls.2015.00615.

Wani, S. H., and Gosal, S. S. (2011). Introduction of OsglyII gene into Oryza sativa for increasing salinity tolerance. *Biol. Plant.* doi:10.1007/s10535-011-0082-y.

Wu, H., Ye, H., Yao, R., Zhang, T., and Xiong, L. (2015). OsJAZ9 acts as a transcriptional regulator in jasmonate signaling and modulates salt stress tolerance in rice. *Plant Sci.* 232, 1–12. doi:10.1016/j.plantsci.2014.12.010.

Wu, T.-M., Lin, W.-R., Kao, Y.-T., Hsu, Y.-T., Yeh, C.-H., Hong, C.-Y., and Kao, C. H. (2013). Identification and characterization of a novel chloroplast/mitochondria co-localized glutathione reductase 3 involved in salt stress response in rice. *Plant Mol. Biol.* 83, 379–90. doi:10.1007/s11103-013-0095-3.

Xia, Z., Wei, Y., Sun, K., Wu, J., Wang, Y., and Wu, K. (2013). The Maize AAA-Type Protein SKD1 Confers Enhanced Salt and Drought Stress Tolerance in Transgenic Tobacco by Interacting with Lyst-Interacting Protein 5. *PLoS One* 8. doi:10.1371/journal.pone.0069787.

Xiang, Y., Huang, Y., and Xiong, L. (2007). Characterization of stress-responsive CIPK genes in rice for stress tolerance improvement. *Plant Physiol.* 144, 1416–28. doi:10.1104/pp.107.101295.

Xiang, Y., Lu, Y. H., Song, M., Wang, Y., Xu, W., Wu, L., Wang, H., Ma, Z., Ostwald, T., MacLennan, D., et al. (2015). Overexpression of a Triticum aestivum Calreticulin gene (TaCRT1) Improves Salinity Tolerance in Tobacco. *PLoS One* 10, e0140591. doi:10.1371/journal.pone.0140591.

Xiong, H., Li, J., Liu, P., Duan, J., Zhao, Y., Guo, X., Li, Y., Zhang, H., Ali, J., and Li, Z. (2014). Overexpression of OsMYB48-1, a novel MYB-related transcription factor, enhances drought and salinity tolerance in rice. *PLoS One* 9, e92913. doi:10.1371/journal.pone.0092913.

Xu, D.-B., Gao, S.-Q., Ma, Y.-Z., Xu, Z.-S., Zhao, C.-P., Tang, Y.-M., Li, X.-Y., Li, L.-C., Chen, Y.-F., and Chen, M. (2014). ABI-like transcription factor gene TaABL1 from wheat improves multiple abiotic stress tolerances in transgenic plants. *Funct. Integr. Genomics* 14, 717–730. doi:10.1007/s10142-014-0394-z.

Xu, D.-Q., Huang, J., Guo, S.-Q., Yang, X., Bao, Y.-M., Tang, H.-J., and Zhang, H.-S. (2008a). Overexpression of a TFIIIA-type zinc finger protein gene ZFP252 enhances drought and salt tolerance in rice (Oryza sativa L.). *FEBS Lett.* 582, 1037–43. doi:10.1016/j.febslet.2008.02.052.

Xu, G.-Y., Rocha, P. S. C. F., Wang, M.-L., Xu, M.-L., Cui, Y.-C., Li, L.-Y., Zhu, Y.-X., and Xia, X. (2011). A novel rice calmodulin-like gene, OsMSR2, enhances drought and salt tolerance and increases ABA sensitivity in Arabidopsis. *Planta* 234, 47–59. doi:10.1007/s00425-011-1386-z.

Xu, Z.-S., Ni, Z.-Y., Liu, L., Nie, L.-N., Li, L.-C., Chen, M., and Ma, Y.-Z. (2008b). Characterization of the TaAIDFa gene encoding a CRT/DRE-binding factor responsive to drought, high-salt, and cold stress in wheat. *Mol. Genet. Genomics* 280, 497–508. doi:10.1007/s00438-008-0382-x.

Yan, S., Li, S., Zhai, G., Lu, P., Deng, H., Zhu, S., Huang, R., Shao, J., Tao, Y., and Zou, G. (2017). Molecular cloning and expression analysis of duplicated polyphenol oxidase genes reveal their functional differentiations in sorghum. *Plant Sci.* 263, 23–30. doi:10.1016/j.plantsci.2017.07.002.

Yang, A., Dai, X., and Zhang, W.-H. (2012). A R2R3-type MYB gene, OsMYB2, is involved in salt, cold, and dehydration tolerance in rice. *J. Exp. Bot.* 63, 2541–56. doi:10.1093/jxb/err431.

Yang, T., Yao, S., Hao, L., Zhao, Y., Lu, W., and Xiao, K. (2016). Wheat bHLH-type transcription factor gene TabHLH1 is crucial in mediating osmotic stresses tolerance through modulating largely the ABA-associated pathway. *Plant Cell Rep.* 35, 2309–2323. doi:10.1007/s00299-016-2036-5.

Ye, H., Du, H., Tang, N., Li, X., and Xiong, L. (2009). Identification and expression profiling analysis of TIFY family genes involved in stress and phytohormone responses in rice. *Plant Mol. Biol.* 71, 291–305. doi:10.1007/s11103-009-9524-8.

Ying, S., Zhang, D.-F., Fu, J., Shi, Y.-S., Song, Y.-C., Wang, T.-Y., and Li, Y. (2012). Cloning and characterization of a maize bZIP transcription factor, ZmbZIP72, confers drought and salt tolerance in transgenic Arabidopsis. *Planta* 235, 253–66. doi:10.1007/s00425-011-1496-7.

Yu, S., Zhang, X., Guan, Q., Takano, T., and Liu, S. (2007). Expression of a carbonic anhydrase gene is induced by environmental stresses in rice (Oryza sativa L.). *Biotechnol. Lett.* 29, 89–94. doi:10.1007/s10529-006-9199-z.

Yu, X., Meng, X., Liu, Y., Li, N., Zhang, A., Wang, T. J., Jiang, L., Pang, J., Zhao, X., Qi, X., et al. (2018). The chromatin remodeler ZmCHB101 impacts expression of osmotic stress-responsive genes in maize. *Plant Mol. Biol.* 97, 451–465. doi:10.1007/s11103-018-0751-8.

Zaidi, I., Ebel, C., Belgaroui, N., Ghorbel, M., Amara, I., and Hanin, M. (2016). The wheat MAP kinase phosphatase 1 alleviates salt stress and increases antioxidant activities in Arabidopsis. *J. Plant Physiol.* 193, 12–21. doi:10.1016/j.jplph.2016.01.011.

Zhai, C. Z., Zhao, L., Yin, L. J., Chen, M., Wang, Q. Y., Li, L. C., Xu, Z. S., and Ma, Y. Z. (2013). Two Wheat Glutathione Peroxidase Genes Whose Products Are Located in Chloroplasts Improve Salt and H2O2 Tolerances in Arabidopsis. *PLoS One* 8. doi:10.1371/journal.pone.0073989.

Zhang, D., Jiang, S., Pan, J., Kong, X., Zhou, Y., Liu, Y., and Li, D. (2014a). The overexpression of a maize mitogen-activated protein kinase gene (ZmMPK5) confers salt stress tolerance and induces defence responses in tobacco. *Plant Biol.* 16, 558–570. doi:10.1111/plb.12084.

Zhang, F., Li, L., Jiao, Z., Chen, Y., Liu, H., Chen, X., Fu, J., Wang, G., and Zheng, J. (2016a). Characterization of the calcineurin B-Like (CBL) gene family in maize and functional analysis of ZmCBL9 under abscisic acid and abiotic stress treatments. *Plant Sci.* 253, 118–129. doi:10.1016/j.plantsci.2016.09.011.

Zhang, H., Mao, X., Jing, R., Chang, X., and Xie, H. (2011). Characterization of a common wheat (Triticum aestivum L.) TaSnRK2.7 gene involved in abiotic stress responses. *J. Exp. Bot.* 62, 975–88. doi:10.1093/jxb/erq328.

Zhang, H., Mao, X., Wang, C., and Jing, R. (2010). Overexpression of a common wheat gene Tasnrk2.8 enhances tolerance to drought, salt and low temperature in Arabidopsis. *PLoS One* 5, e16041. doi:10.1371/journal.pone.0016041.

Zhang, J.-S., Xie, C., Li, Z.-Y., and Chen, S.-Y. (1999). Expression of the plasma membrane H + -ATPase gene in response to salt stress in a rice salt-tolerant mutant and its original variety. *TAG Theor. Appl. Genet.* 99, 1006–1011. doi:10.1007/s001220051408.

Zhang, L., Liu, G., Zhao, G., Xia, C., Jia, J., Liu, X., and Kong, X. (2014b). Characterization of a Wheat R2R3-MYB Transcription Factor Gene, TaMYB19, Involved in Enhanced Abiotic Stresses in Arabidopsis. *Plant Cell Physiol.* 55, 1802–1812. doi:10.1093/pcp/pcu109.

Zhang, L., Zhang, L., Xia, C., Zhao, G., Jia, J., and Kong, X. (2016b). The Novel Wheat Transcription Factor TaNAC47 Enhances Multiple Abiotic Stress Tolerances in Transgenic Plants. *Front. Plant Sci.* 6, 1174. doi:10.3389/fpls.2015.01174.

Zhang, L., Zhang, L., Xia, C., Zhao, G., Liu, J., Jia, J., and Kong, X. (2015). A novel wheat bZIP transcription factor, TabZIP60, confers multiple abiotic stress tolerances in transgenic Arabidopsis. *Physiol. Plant.* 153, 538–554. doi:10.1111/ppl.12261.

Zhang, L., Zhao, G., Xia, C., Jia, J., Liu, X., and Kong, X. (2012a). A wheat R2R3-MYB gene, TaMYB30-B, improves drought stress tolerance in transgenic Arabidopsis. *J. Exp. Bot.* 63, 5873–5885. doi:10.1093/jxb/ers237.

Zhang, L., Zhao, G., Xia, C., Jia, J., Liu, X., and Kong, X. (2012b). Overexpression of a wheat MYB transcription factor gene, TaMYB56-B, enhances tolerances to freezing and salt stresses in transgenic Arabidopsis. *Gene* 505, 100–107. doi:10.1016/j.gene.2012.05.033.

Zhang, Y. M., Zhang, H. M., Liu, Z. H., Li, H. C., Guo, X. L., and Li, G. L. (2014c). The wheat NHX antiporter gene TaNHX2 confers salt tolerance in transgenic alfalfa by increasing the retention capacity of intracellular potassium. *Plant Mol. Biol.* 87, 317–327. doi:10.1007/s11103-014-0278-6.

Zhao, B., Ge, L., Liang, R., Li, W., Ruan, K., Lin, H., and Jin, Y. (2009). Members of miR-169 family are induced by high salinity and transiently inhibit the NF-YA transcription factor. *BMC Mol. Biol.* 10, 29. doi:10.1186/1471-2199-10-29.

Zhao, Y., Ma, Q., Jin, X., Peng, X., Liu, J., Deng, L., Yan, H., Sheng, L., Jiang, H., and Cheng, B. (2014). A novel maize homeodomain-leucine zipper (HD-Zip) i gene, Zmhdz10, positively regulates drought and salt tolerance in both rice and arabidopsis. *Plant Cell Physiol.* 55, 1142–1156. doi:10.1093/pcp/pcu054.

Zheng, X., Chen, B., Lu, G., and Han, B. (2009). Overexpression of a NAC transcription factor enhances rice drought and salt tolerance. *Biochem. Biophys. Res. Commun.* 379, 985–9. doi:10.1016/j.bbrc.2008.12.163.

Zhou, J., Li, F., Wang, J.-L., Ma, Y., Chong, K., and Xu, Y. (2009). Basic helix-loop-helix transcription factor from wild rice (OrbHLH2) improves tolerance to salt- and osmotic stress in Arabidopsis. *J. Plant Physiol.* 166, 1296–306. doi:10.1016/j.jplph.2009.02.007.

Zhou, L., Zhou, J., Xiong, Y., Liu, C., Wang, J., Wang, G., and Cai, Y. (2018). Overexpression of a maize plasma membrane intrinsic protein ZmPIP1;1 confers drought and salt tolerance in Arabidopsis. *PLoS One* 13. doi:10.1371/journal.pone.0198639.
