# Supplementary material for: Identification and development of novel salt-responsive candidate gene based SSRs (cg-SSRs) and MIR gene based SSRs (mir-SSRs) in bread wheat (Triticum aestivum)
Source: Sci Rep. 2021 Jan 26;11:2210. doi: 10.1038/s41598-021-81698-3 (PMC7838269; doi:10.1038/s41598-021-81698-3)
Supplement: Supplementary file 3 — Supplementary Table S4. [file 41598_2021_81698_MOESM3_ESM.docx]

**Identification and development of novel salt-responsive candidate gene based SSRs (cg-SSRs) and *MIR* gene based SSRs (mir-SSRs) in bread wheat (*Triticum aestivum*)**

Geetika Mehta^1#^, Senthilkumar K Muthusamy^1, 2 #^, G. P. Singh^1^, Pradeep Sharma^1,^ *

^1^Division of Crop Improvement, ICAR-Indian Institute of Wheat and Barley Research, Karnal, India

^2^Division of Crop Improvement, ICAR-Central Tuber Crops Research Institute, Thiruvananthapuram, India

^#^Equal contribution

**Supplementary Table S4** Salt stress responsive genes in Wheat. aa- amino acids; pI- isoelectric point. Mw-Molecular weight; the subcellular localization of proteins were predicted through WoLF PSORT and TargetP 1.1 server.

| **Gene** | **Gene Description** | **Ensembl Plants gene ID** | **Chromosomal location** | **Gene Start** | **Gene end** | **Gene orientation** | **Sub-Cellular localization** |
| --- | --- | --- | --- | --- | --- | --- | --- |
| *TaMKP1* | Kinase | TraesCS1A02G045300.1 | 1A | 26958795 | 26962687 | -1 | Endoplasmic reticulum |
| *TaECS* | antioxidation | TraesCS1A02G072000.1 | 1A | 54349175 | 54355250 | 1 | Chloroplast |
| *TaGGT* | Glycosylation | TraesCS1A02G211500.1 | 1A | 374214039 | 374217445 | 1 | Cytoplasm |
| *TaYqgF* | Regulatory, RNA binding | TraesCS1A02G211600.1 | 1A | 374217713 | 374219255 | -1 | cytoplasm |
| *TaDREB1F* | TF | TraesCS1A02G220000.1 | 1A | 388849047 | 388850291 | 1 | Nucleus |
| *TaGBF1* | G-box binding factor 1 | TraesCS1A02G409800.2 | 1A | 571286613 | 571291700 | 1 | Nucleus |
| *TaCML11* | Regulatory, Ca2+-binding | TraesCS1B02G208700.1 | 1B | 378462268 | 378463337 | 1 | Nucleus |
| *TaWRKY-13* | TF | TraesCS1B02G212000.1 | 1B | 385295925 | 385297471 | 1 | Nucleus |
| *TaEXPB23* | Expansin | TraesCS1B02G226000.2 | 1B | 405904908 | 405906612 | -1 | Extra-cellular |
| *TaMT1e-P* | Cysteine-rich, metal binding | TraesCS1B02G239900.1 | 1B | 427776745 | 427777360 | 1 | Mitochondria |
| *TaWRKY2* | TF | TraesCS1D02G072900.1 | 1D | 53379717 | 53381850 | -1 | Nucleus |
| *TaPIMP1* | TF | TraesCS1D02G078600.1 | 1D | 60316834 | 60318526 | 1 | Nucleus |
| *SR3WRSI5* | protease inhibitor | TraesCS1D02G266000.1 | 1D | 361907486 | 361908378 | 1 | Extra-cellular |
| *TaMYB19* | TF | TraesCS1D02G268900.1 | 1D | 364624035 | 364625440 | -1 | Nucleus |
| *TdSHN1* | TF | TraesCS1D02G314800.1 | 1D | 410071174 | 410072405 | -1 | Nucleus |
| *TaTPS1* | Trehalose biosynthesis | TraesCS1D02G341100.2 | 1D | 430976701 | 430983376 | -1 | Cytoplasm |
| *TaSOS3* | Transporter | TraesCS1D02G358200.1 | 1D | 441749850 | 441751968 | 1 | Plasma membrane |
| *TaNHX2* | Transporter | TraesCS2A02G121000.1 | 2A | 70876938 | 70881306 | -1 | Plasma membrane |
| *TaRSL4A* | TF | TraesCS2A02G194200.1 | 2A | 162291365 | 162292945 | 1 | Nucleus |
| *TaWNK1* | Signalling, Kinase | TraesCS2A02G195900.2 | 2A | 165847896 | 165850719 | -1 | Nucleus |
| *TaRSL4 A-homeolog* | TF | TraesCS2A02G235400.1 | 2A | 293151798 | 293154179 | -1 | Nucleus |
| *TaAPX7* | antioxidation | TraesCS2A02G305600.2 | 2A | 525616102 | 525627082 | -1 | Chloroplast |
| *TaDBH1* | DEAD- box, ATP dependent RNA helicase | TraesCS2A02G345300.1 | 2A | 583827561 | 583836236 | 1 | Chloroplast |
| *TaAQP8* | aquaporin | TraesCS2A02G407700.1 | 2A | 663327624 | 663330251 | -1 | Plasma membrane |
| *TaBIERF3* | TF | TraesCS2A02G417300.1 | 2A | 673326836 | 673328368 | 1 | Nucleus |
| *TaAOX1a* | electron transport | TraesCS2A02G438200.1 | 2A | 689630218 | 689631640 | -1 | Chloroplast |
| *TaPP2C1* | protein phosphatase | TraesCS2A02G500200.1 | 2A | 729283043 | 729287271 | 1 | Nucleus / Cytoplasm |
| *PI4K* | Phosphatidylinositol (PI) 4-kinases | TraesCS2A02G540100.1 | 2A | 752276283 | 752278181 | -1 | Peroxisome |
| *W106* | Glutathione Peroxidase | TraesCS2A02G582100.1 | 2A | 774571911 | 774573949 | 1 | Cytoplasm |
| *TaOPR1* | Functional, Oxophytodienoate Reductase | TraesCS2B02G040000.1 | 2B | 18177103 | 18178722 | -1 | Cytoplasm |
| *TaAPXb* | Antioxidation | TraesCS2B02G096200.1 | 2B | 56517744 | 56521691 | 1 | Mitochondria |
| *TaHsfA7* | TF, Chaperon | TraesCS2B02G105100.1 | 2B | 65370326 | 65375768 | 1 | Nucleus |
| *TaMyb2* | TF | TraesCS2B02G112600.1 | 2B | 75847314 | 75848794 | 1 | Nucleus |
| *Tar40c1* | ABA responsive protein | TraesCS2B02G118700.1 | 2B | 84816250 | 84818045 | 1 | Cytoplasm |
| *TaCIPK29* | Kinase | TraesCS2B02G124100.1 | 2B | 92489631 | 92490941 | -1 | Chloroplast |
| *TaMYB30-B* | TF | TraesCS2B02G184500.2 | 2B | 159362947 | 159368040 | 1 | Nucleus |
| *TaWRKY45* | TF | TraesCS2B02G187500.1 | 2B | 162872445 | 162873846 | -1 | Nucleus |
| *TaHAP2E* | TF | TraesCS2B02G198700.1 | 2B | 176406136 | 176409334 | 1 | Nucleus |
| *TaWRKY19* | TF | TraesCS2B02G209200.1 | 2B | 190968612 | 190972154 | 1 | Nucleus |
| *TaRSL4B* | TF | TraesCS2B02G212700.1 | 2B | 197210852 | 197212507 | -1 | Nucleus |
| *TaC3H50* | Regulatory, RNA processing | TraesCS2B02G227500.1 | 2B | 221086597 | 221089766 | 1 | Nucleus/cytoplasm |
| *TaCLC-1* | Ion transport | TraesCS2B02G326900.1 | 2B | 468238404 | 468243493 | -1 | Chloroplast |
| *TaSAMDC* | polyamine biosynthesis | TraesCS2B02G372900.1 | 2B | 531466716 | 531467891 | 1 | Cytoplasm |
| *TaSKC1* | ion-transporter | TraesCS2B02G451800.1 | 2B | 645470305 | 645474469 | 1 | Vacuole |
| *TaHsp90* | Heat shock | TraesCS2D02G033200.2 | 2D | 12882405 | 12885584 | -1 | Cytoplasm |
| *TaCam1-1* | Signalling | TraesCS2D02G097600.1 | 2D | 49878305 | 49880246 | 1 | Cytoplasm |
| *TaRSL4D* | TF | TraesCS2D02G193700.1 | 2D | 138754346 | 138756038 | -1 | Nucleus |
| *TaSRG* | TF | TraesCS2D02G290400.1 | 2D | 372695288 | 372696521 | -1 | Nucleus |
| *TaSnRK2.7* | Kinase | TraesCS2D02G302500.1 | 2D | 386248395 | 386253037 | 1 | Cytoplasm |
| *TaERF4* | TF | TraesCS2D02G543900.1 | 2D | 621404177 | 621405692 | -1 | Nucleus |
| *TaCRT1* | Calreticulin family protein | TraesCS2D02G546500.2 | 2D | 622922900 | 622926885 | -1 | Endoplasmic reticulum |
| *TaSOS1* | Transporter | TraesCS3A02G023200.2 | 3A | 12969505 | 12980909 | 1 | Plasma membrane |
| *TaHBP1b* | TF | TraesCS3A02G190700.2 | 3A | 238676584 | 238691290 | -1 | Nucleus |
| *TaCA1* | carbonic anahydrase | TraesCS3A02G230000.2 | 3A | 430330494 | 430337814 | 1 | Cytoplasm |
| *TaTPC1* | Ion-channel | TraesCS3A02G268700.3 | 3A | 494996019 | 495015975 | 1 | Vacuole |
| *TaSOS2* | Signalling, Kinase | TraesCS3A02G346100.1 | 3A | 595433942 | 595436796 | 1 | Cytoplasm |
| *TaST* | Unknown function | TraesCS3A02G415300.1 | 3A | 658700863 | 658703196 | -1 | Vacuolar |
| *OrbHLH001* | TF | TraesCS3A02G442200.1 | 3A | 684289775 | 684293025 | -1 | Chloroplast |
| *TaCML31* | Regulatory, Ca2+-binding | TraesCS3A02G492700.1 | 3A | 719387115 | 719387561 | 1 | Cytoplasm |
| *TaPUB15* | ubiquitination | TraesCS3A02G521300.1 | 3A | 737968002 | 737975248 | -1 | Chloroplast |
| *TaAIDFa* | TF | TraesCS3B02G115400.1 | 3B | 82039696 | 82053894 | -1 | Nucleus |
| *TaSIP* | Unknown function | TraesCS3B02G123100.1 | 3B | 94855973 | 94857595 | 1 | chloroplast |
| *TaTZF1* | TF,ZF | TraesCS3B02G125600.1 | 3B | 101799238 | 101802140 | 1 | Nucleus |
| *TaAP21* | TF | TraesCS3B02G158800.1 | 3B | 154195711 | 154197010 | 1 | Nucleus |
| *TaSST* | Unknown function | TraesCS3B02G209100.1 | 3B | 245357884 | 245359927 | 1 | Endoplasmic reticulum |
| *TaDi19A* | Unknown function | TraesCS3B02G274000.2 | 3B | 441280224 | 441283537 | 1 | Cytoplasm |
| *TaRab7* | Signalling, GTP binding | TraesCS3B02G324900.1 | 3B | 525829746 | 525832899 | -1 | Chloroplast |
| *TaCML8* | Regulatory, Ca2+-binding | TraesCS3B02G365900.2 | 3B | 578399102 | 578403477 | 1 | Nucleus |
| *TaEXPA2* | Functional, cell wall | TraesCS3B02G376800.1 | 3B | 593163474 | 593165215 | 1 | Chloroplast |
| *TaMYB3R1* | TF | TraesCS3B02G391900.1 | 3B | 617428914 | 617434813 | 1 | Nucleus |
| *TaSERF1* | TF, Inhibition of MAPK cascade | TraesCS3B02G437900.1 | 3B | 676943838 | 676944586 | -1 | Nucleus |
| *TaSNAC2* | TF | TraesCS3B02G439600.1 | 3B | 678548127 | 678550377 | 1 | Nucleus |
| *TaBADH1* | Functional, Osmoprotection | TraesCS3B02G452100.1 | 3B | 693072552 | 693077229 | 1 | Cytoplasm |
| *TaWRKY10* | TF | TraesCS3D02G113200.1 | 3D | 67350958 | 67351930 | 1 | Nucleus |
| *TaCyP20–2* | Functional, Protein folding | TraesCS3D02G159000.1 | 3D | 128439351 | 128445708 | 1 | Cytoplasm |
| *TaMyb3R-2* | TF | TraesCS3D02G353500.1 | 3D | 464161767 | 464168144 | 1 | Mitochondria |
| *TaSnRK2.4* | Kinase | TraesCS3D02G374300.1 | 3D | 487807055 | 487811579 | 1 | Cytoplasm |
| *TaP5CR* | Osmoprotection | TraesCS3D02G483300.2 | 3D | 580718491 | 580720926 | 1 | Nucleus |
| *TaMYB56-B* | TF | TraesCS3D02G540600.1 | 3D | 611918053 | 611919686 | -1 | Nucleus |
| *TaTIFY11a* | Regulatory | TraesCS4A02G008000.1 | 4A | 4847654 | 4848933 | 1 | Nucleus |
| *TaGR3* | Reduces GSSG | TraesCS4A02G025200.1 | 4A | 17263279 | 17267968 | -1 | Chloroplast |
| *TaSRWD5* | Regulatory, Chromatin modification,Transcription | TraesCS4A02G093000.1 | 4A | 100559355 | 100564319 | -1 | Nucleus |
| *TaABCG5* | Transporter | TraesCS4A02G103400.1 | 4A | 116850006 | 116852902 | -1 | Chloroplast |
| *TaTOP6A3* | Topoisomerase | TraesCS4A02G105600.1 | 4A | 119781613 | 119783780 | 1 | Chloroplast |
| *TaMYB* | TF | TraesCS4A02G189700.1 | 4A | 468603185 | 468604647 | 1 | Nucleus |
| *TaACO1* | aminocyclopropane-1-carboxylate oxidase | TraesCS4A02G221300.1 | 4A | 527001442 | 527003224 | 1 | Cytoplasm |
| *TaCBSX4* | Regulatory, Adenosine binding | TraesCS4A02G247300.1 | 4A | 558414421 | 558418589 | 1 | Chloroplast |
| *TaBIHD1* | TF (HD) | TraesCS4B02G025500.1 | 4B | 18162017 | 18167541 | -1 | Nucleus |
| *TaOXHS2* | Regulatory, protein-protein interaction | TraesCS4B02G118300.1 | 4B | 138299948 | 138306480 | -1 | Cytoplasm |
| *TaCIPK14* | Kinase | TraesCS4B02G120400.1 | 4B | 141427582 | 141428916 | -1 | Chloroplast |
| *W69* | Glutathione Peroxidase | TraesCS4B02G152800.1 | 4B | 256324840 | 256340340 | 1 | Chloroplast |
| *TaWRKY44* | TF | TraesCS4B02G165900.2 | 4B | 352135873 | 352170119 | -1 | Nucleus |
| *TaMAPK44* | Signalling, Kinase | TraesCS4B02G197800.1 | 4B | 426767061 | 426769719 | 1 | Cytoplasm |
| *TaSDIR1* | ubiquitination | TraesCS4B02G208600.2 | 4B | 445842921 | 445845979 | -1 | Chloroplast |
| *TaSOD2* | superoxide dismutase | TraesCS4B02G243200.1 | 4B | 504157374 | 504161203 | 1 | Cytoplasm |
| *TaCPK17* | Signalling, Kinase | TraesCS4D02G125500.1 | 4D | 109909795 | 109912155 | -1 | Cytoplasm |
| *TNHXS1* | Transporter | TraesCS4D02G147600.1 | 4D | 139042047 | 139047298 | 1 | Vacuolar |
| *TaNAC* | TF | TraesCS4D02G175700.1 | 4D | 304689911 | 304692359 | 1 | Nucleus |
| *TaRINO1* | Myoinositol synthesis | TraesCS4D02G266700.2 | 4D | 437514756 | 437519444 | -1 | Cytoplasm |
| *TaERF922* | TF | TraesCS4D02G315000.1 | 4D | 480920143 | 480920769 | -1 | Nucleus |
| *TaPEX11-1* | Functional, peroxisomal biogenesis | TraesCS4D02G348600.1 | 4D | 502072761 | 502074897 | 1 | Chloroplast |
| *TmHKT8* | Transporter | TraesCS4D02G361300.1 | 4D | 507965542 | 507967822 | 1 | Plasma membrane |
| *TaCIPK15* | Signalling | TraesCS5A02G148000.1 | 5A | 325930589 | 325933350 | 1 | Cytoplasm |
| *TaSnRK2.8* | Kinase | TraesCS5A02G401700.1 | 5A | 594570844 | 594577495 | 1 | Cytoplasm |
| *TaGly-I* | glyoxalase | TraesCS5A02G454900.1 | 5A | 635593690 | 635596159 | -1 | Chloroplast |
| *TaNAC2* | TF | TraesCS5A02G468300.1 | 5A | 645726140 | 645727775 | 1 | Nucleus |
| *TaSP* | Unknown function | TraesCS5B02G010400.2 | 5B | 10261716 | 10265365 | -1 | chloroplast |
| *TaZFP252* | TF | TraesCS5B02G054400.1 | 5B | 59645667 | 59647455 | 1 | Nucleus |
| *TaCML5* | Signalling | TraesCS5B02G059900.1 | 5B | 66053051 | 66053557 | -1 | Mitochondria |
| *TaPOP5* | Serine peptidases | TraesCS5B02G104300.1 | 5B | 139166342 | 139173361 | -1 | Cytoplasm |
| *TaC3H33* | Regulatory, RNA processing | TraesCS5B02G105500.1 | 5B | 141572501 | 141575005 | -1 | Nucleus/cytoplasm |
| *TaSC* | Unknown function | TraesCS5B02G233800.1 | 5B | 411807809 | 411811143 | -1 | Vacuolar |
| *TaglyII* | Functional, glyoxalate-pathway | TraesCS5B02G303100.1 | 5B | 487597910 | 487603206 | -1 | Chloroplast |
| *TaDREB1A* | TF | TraesCS5B02G313000.1 | 5B | 494315159 | 494316116 | -1 | Nucleus |
| *TaUGE-1* | nucleotide sugar interconversion | TraesCS5B02G315700.1 | 5B | 497903499 | 497905717 | -1 | Cytoplasm |
| *TaCPK-21* | Signalling, Kinase | TraesCS5B02G364000.1 | 5B | 542934945 | 542936732 | 1 | Nucleus |
| *TaNAC5* | TF | TraesCS5B02G480900.2 | 5B | 653432387 | 653434033 | 1 | Nucleus |
| *Ta-sro1* | poly(ADP ribose) polymerase like activity | TraesCS5B02G557500.2 | 5B | 705276580 | 705282390 | -1 | Nucleus |
| *TaACA6* | Ca2+ Atpase | TraesCS5D02G080700.1 | 5D | 80699076 | 80704304 | -1 | Chloroplast |
| *TabZIP71* | TF | TraesCS5D02G183500.1 | 5D | 285247655 | 285248540 | 1 | Chloroplast |
| *TaDST* | TF, H2O2-homeostasis | TraesCS5D02G433400.1 | 5D | 489188483 | 489189301 | 1 | Mitochondria |
| *TaZFP182* | TF | TraesCS5D02G490300.1 | 5D | 525118825 | 525119459 | -1 | Nucleus |
| *TaSK5* | GSK3/shaggy-like kinase | TraesCS5D02G512300.1 | 5D | 536536574 | 536541626 | -1 | Cytoplasm |
| *TaNAC47* | TF | TraesCS6A02G057400.1 | 6A | 30190380 | 30191252 | -1 | Nucleus |
| *TaCAX* | Ion-channel | TraesCS6A02G108700.1 | 6A | 77532734 | 77535973 | 1 | Endoplasmic reticulum |
| *TaWRKY93* | TF | TraesCS6A02G146900.1 | 6A | 126344720 | 126346757 | -1 | Nucleus |
| *TaMYB33* | TF | TraesCS6A02G224400.1 | 6A | 421463673 | 421464869 | 1 | Nucleus |
| *TaABL1* | TF | TraesCS6A02G333600.2 | 6A | 564715783 | 564720341 | 1 | Nucleus |
| *TaNAC67* | TF | TraesCS6B02G075200.1 | 6B | 51579298 | 51580659 | -1 | Nucleus |
| *TaSRZ1* | Regulatory, Splicing | TraesCS6B02G209100.2 | 6B | 274984749 | 274992066 | -1 | Chloroplast |
| *Tamyb4* | TF | TraesCS6B02G257000.1 | 6B | 461393685 | 461394831 | -1 | Nucleus |
| *TaMGD* | Lipid Biosynthesis | TraesCS6B02G404700.1 | 6B | 680306482 | 680311300 | 1 | Cytoplasm |
| *TaNOA1* | Functional, NO synthesis | TraesCS6D02G017000.2 | 6D | 7094960 | 7098896 | 1 | Cytoplasm |
| *TaRacB* | Signalling, GTPase | TraesCS6D02G068500.2 | 6D | 34394637 | 34398291 | 1 | Chloroplast |
| *TaMSRMK3* | Signalling, Kinase | TraesCS6D02G108100.1 | 6D | 72229215 | 72231632 | -1 | Cytoplasm |
| *TaiSAP8* | Signalling, protein binding | TraesCS6D02G154100.1 | 6D | 129151102 | 129152165 | 1 | Chloroplast |
| *TaCDKC-1* | Regulatory, Kinase | TraesCS6D02G184000.1 | 6D | 226780042 | 226788676 | -1 | Cytoplasm |
| *TaMYB73* | TF | TraesCS6D02G211400.1 | 6D | 298443642 | 298444944 | -1 | Nucleus |
| *TaDSM1* | Signalling, Kinase | TraesCS6D02G287700.2 | 6D | 396917329 | 396928967 | -1 | Cytoplasm |
| *TaSKIPa* | Regulatory, Spliceosome component | TraesCS6D02G294500.1 | 6D | 405472025 | 405474365 | 1 | Nucleus |
| *TaAOC1* | allene oxide cyclase | TraesCS6D02G314300.1 | 6D | 422610993 | 422612185 | 1 | Chloroplast |
| *H+- ATPase/OSA3* | Ion-transporter | TraesCS6D02G344000.1 | 6D | 443846675 | 443850562 | -1 | Chloroplast |
| *TaSRWD3* | Regulatory, Chromatin modification,Transcription | TraesCS7A02G132700.1 | 7A | 85336219 | 85345447 | 1 | Nucleus |
| *TaMYBsdu1* | TF | TraesCS7A02G205100.1 | 7A | 167179571 | 167180917 | 1 | Nucleus |
| *TaCIPK25* | CBL-interacting protein kinase | TraesCS7A02G352200.1 | 7A | 515879021 | 515880777 | -1 | Cytoplasm |
| *TaMIOX* | Balances the concentration of myo-inositol | TraesCS7A02G357800.1 | 7A | 526784744 | 526788380 | 1 | Cytoplasm |
| *TVP1* | Functional, H+-pyrophosphatase | TraesCS7A02G517700.2 | 7A | 701638088 | 701642904 | -1 | Vacuolar |
| *ONAC045* | TF | TraesCS7B02G056300.1 | 7B | 59179003 | 59180628 | -1 | Nucleus |
| *TaERF1* | TF | TraesCS7B02G062200.1 | 7B | 65660420 | 65663385 | 1 | Nucleus |
| *Ta-UnP* | Unknown function | TraesCS7B02G067400.1 | 7B | 72763900 | 72764955 | -1 | Nucleus |
| *TaSRWD2* | Regulatory, Chromatin modification,Transcription | TraesCS7B02G127900.1 | 7B | 153323473 | 153329253 | 1 | Nucleus |
| *TaSRWD4* | Regulatory, Chromatin modification,Transcription | TraesCS7B02G178000.1 | 7B | 255999437 | 256017099 | -1 | Cytoplasm |
| *TaABP* | Regulatory, Helicase | TraesCS7B02G232200.1 | 7B | 435121892 | 435130105 | -1 | Nucleus |
| *TaMSRB* | antioxidation | TraesCS7B02G247500.2 | 7B | 457633813 | 457636085 | 1 | Chloroplast |
| *TaHKT2;1* | Transporter | TraesCS7B02G318400.1 | 7B | 568488183 | 568490598 | 1 | Plasma membrane |
| *TaWRKY79* | TF | TraesCS7B02G418500.2 | 7B | 686886913 | 686888542 | -1 | Nucleus |
| *TaNIP* | aquaporin | TraesCS7D02G188800.2 | 7D | 141185115 | 141188913 | -1 | Endoplasmic reticulum |
| *OrbHLH2* | TF | TraesCS7D02G308300.1 | 7D | 390689894 | 390693353 | -1 | Nucleus |
| *TaMAPK1* | Kinase | TraesCS7D02G403700.2 | 7D | 520827434 | 520832219 | -1 | Cytoplasm |
| *TaCHP* | TF | TraesCS7D02G545700.1 | 7D | 632781536 | 632782649 | 1 | Nucleus |
| *TaAKT1* | Ion transport | TraesCSU02G182900.1 | Un | 276526691 | 276530298 | 1 | Chloroplast |
