# Supplementary material for: Identification and development of novel salt-responsive candidate gene based SSRs (cg-SSRs) and MIR gene based SSRs (mir-SSRs) in bread wheat (Triticum aestivum)
Source: Sci Rep. 2021 Jan 26;11:2210. doi: 10.1038/s41598-021-81698-3 (PMC7838269; doi:10.1038/s41598-021-81698-3)
Supplement: Supplementary file 5 — Supplementary Table S6. [file 41598_2021_81698_MOESM5_ESM.docx]

**Identification and development of novel salt-responsive candidate gene based SSRs (cg-SSRs) and *MIR* gene based SSRs (mir-SSRs) in bread wheat (*Triticum aestivum*)**

Geetika Mehta^1#^, Senthilkumar K Muthusamy^1, 2 #^, G. P. Singh^1^, Pradeep Sharma^1,^ *

^1^Division of Crop Improvement, ICAR-Indian Institute of Wheat and Barley Research, Karnal, India

^2^Division of Crop Improvement, ICAR-Central Tuber Crops Research Institute, Thiruvananthapuram, India

^#^Equal contribution

**Supplementary Table S6 Details of the primers used in qPCR expression study**

| **Genes/Transcripts** | **Forward Primer** | **Reverse Primer** |
| --- | --- | --- |
| TaDSM1 | GATGATAGGAAGCCACGTTAC | CGCCGCAACTATGCTTAT |
| TabZIP71 | GATGATAGAACCGCAGTAGC | GGTTAGGGAGAAGACGTAGA |
| TaSRZ1 | TGAGCAGAAGCAGCAAAG | GGTCCCAAATGGGAAATGA |
| TaMyb2 | ATAATCCCGTCTCCTCTTCC | CCAATCACTCCGTATTTACCC |
| TaTPC1 | CCGGTCTTATTGGGACATAC | GTGGTGAAGAGGACAAACA |
| TaDBH1 | AGGAGCTGTTCTGCTCTAA | CACGTCCAGTTTCCTGTATG |
| TaCML11 | AGATGAGCTGTTGTCTGTTATG | GACCATCTTGCGAGATTTAGG |
| TaSAMDC | TTCTCCCACCAATCTCTCT | CTTCTCGTAGCCCTCAAATC |
| Actin | CAAATCATGTTTGAGACCTTCAATG | ACCAGAATCCAACACGATACCTG |
